# Comparative analysis of the fecal microbiota from different species of domesticated and wild suids

**DOI:** 10.1038/s41598-019-49897-1

**Published:** 2019-09-20

**Authors:** Florencia Correa-Fiz, Miguel Blanco-Fuertes, Maria J. Navas, Anna Lacasta, Richard P. Bishop, Naftaly Githaka, Cynthia Onzere, Marie-Frédérique Le Potier, Vanessa Almagro-Delgado, Jorge Martinez, Virginia Aragon, Fernando Rodriguez

**Affiliations:** 1 IRTA, Centre de Recerca en Sanitat Animal (CReSA, IRTA-UAB), Campus de la Universitat Autònoma de Barcelona, 08193 Bellaterra, Spain; 2 International Livestock Research Institute (ILRI), P.O. Box 30709, 00100 Nairobi, Kenya; 3 Washington State University, Department of Veterinary Microbiology and Pathology, Pullman, WA USA; 4 ANSES, laboratory of Ploufragan-Plouzané-Niort, swine virology and immunology unit, 22440 Ploufragran, France; 5 Veterinary service Zoo Barcelona, Parc Ciudadella s/n 08003, Barcelona, Spain; 6 Departament de Sanitat i Anatomia Animals, Universitat Autònoma de Barcelona, 08193 Bellaterra, Spain

**Keywords:** Computational biology and bioinformatics, Microbial communities

## Abstract

Most of the microorganisms living in a symbiotic relationship in different animal body sites (microbiota) reside in the gastrointestinal tract (GIT). Several studies have shown that the microbiota is involved in host susceptibilities to pathogens. The fecal microbiota of domestic and wild suids was analyzed. Bacterial communities were determined from feces obtained from domestic pigs (*Sus scrofa*) raised under different conditions: specific-pathogen-free (SPF) pigs and domestic pigs from the same bred, and indigenous domestic pigs from a backyard farm in Kenya. Secondly, the fecal microbiota composition of the African swine fever (ASF) resistant warthogs (*Phacochoerus africanus*) from Africa and a European zoo was determined. African swine fever (ASF) is a devastating disease for domestic pigs. African animals showed the highest microbial diversity while the SPF pigs the lowest. Analysis of the core microbiota from warthogs (resistant to ASF) and pigs (susceptible to ASF) showed 45 shared OTUs, while 6 OTUs were exclusively present in resistant animals. These six OTUs were members of the *Moraxellaceae* family, *Pseudomonadales* order and *Paludibacter*, *Anaeroplasma*, *Petrimonas*, and *Moraxella* genera. Further characterization of these microbial communities should be performed to determine the potential involvement in ASF resistance.

## Introduction

The characterization of the microbiota diversity inhabiting particular mucosal surfaces or other body sites has been an active topic of research in recent years, due to the involvement in many vital processes. In the gut, the high microbial diversity has the potential to provide metabolic activities that the host lacks. Specifically, the gut microbiota of mammals has been shown to confer health benefits to the host through the production of digestible food components, inhibition and prevention of colonization by pathogens, and development and maintenance of the host immune system^1–3^.

The microbiota composition from the pigs’ gastrointestinal tract (GIT) has been subject of many investigations. Piglets are first exposed to microbes at birth and they will eventually be colonized with different microbial populations by constant exposure to microbes^4^. Early gut colonization is critically for both morphological and immunological development of the GIT^5^. During growth, the microbiome changes exhibiting increased diversity, a relevant indicator of GIT health^4^. Once established, the microbiota ultimately reaches an equilibrated community composition^6^ that is essential in maintaining the health of the animal. However, under several pathological conditions or antimicrobial treatments, disruption of the microbiota may occur leading to a dysbiosis state. Moreover, there are other important factors that may impact the GIT microbiota composition such as diet^6,7^, age^8,9^ and the environment where animals are raised^10^. Intensive farming has led to pigs being mostly raised indoors, where they are exposed to a limited diversity of microorganisms. The extreme cases are germ-free laboratory animals, grown under conditions that totally restrict microbial exposure. These animals show physiological and behavioral abnormalities with impaired development of the immune system^11^. Given the complexity of breeding germ-free pigs, the vast majority of animal experiments are performed with specific pathogen-free (SPF) pigs, raised under a highly controlled environment free of certain potentially pathogenic microorganisms (bacteria, viruses and parasites). SPF animals have a different degree of maturation of the innate immune system when compared with the domestic counterparts^12^. This impaired immune system has been also associated with increased disease susceptibility, including African swine fever^13^.

African swine fever (ASF) is a devastating hemorrhagic disease of pigs caused by a large DNA virus with mortality rates near 100%^14^. ASF has been mainly confined to sub-Saharan Africa in a sylvatic cycle including wild suids and arthropod vectors together with domestic pigs. Recently, it has spread out of Africa and into the Russian Federation, China, and Europe, heightening awareness of threat to the global pig industry^15^. With no vaccines or treatment available for this disease, it is of urgent need to develop alternative means of control. With the exception of African wild pigs^16,17^ (warthogs and bushpigs), all pigs are susceptible to the infection with virulent strains of ASFV. It has been hypothesized that African domestic pigs may have genetic characteristics related to ASFV tolerance, but these genomic signatures have not been clearly detected^18^. SPF pigs, conventional domestic pigs and wild boars, succumb after experimental challenge with a highly virulent strain of ASFV, independently of their sex and age^19^. Additional evidences from our lab demonstrated that SPF pigs infected with intermediate doses of attenuated strains of the African swine fever virus, although safe for domestic pigs, resulted in a lethal outcome for 100% of the SPF animals^13^. The reasons behind this enhanced susceptibility of SPF pigs may be partially explained by a different and less diverse gut microbiota composition, since both SPF and domestic pigs are both from the same breed. The association between a more diverse and rich microbiota and a healthier state has been demonstrated not only for livestock^20^, but also for laboratory animals^21^ and humans^22,23^. African warthogs are known to be natural reservoir hosts of ASFV in the wild. Under experimental conditions, captive warthogs also demonstrated resistance to ASFV infection, showing no clinical signs of disease when infected with the same highly virulent isolates of ASFV that induce rapid, hemorrhagic death in domestic pigs^24–26^. Being aware that pigs (*Sus scrofa*) and warthogs (*Phacochoerus africanus*) belong to different species, and that genetic differences will most probably play key roles in differential susceptibility, we hypothesized that the warthog GIT microbiota could contribute to the ASFV resistance phenotype. The fecal microbial populations of these animal species have not been thoroughly studied^27^, hence the knowledge of the microorganisms inhabiting the gut of wild animals can provide insight into their potential role on resistance to diseases.

In this study we aimed to firstly, determine the microbial composition of specific-pathogen-free (SPF) pigs, which exhibit higher susceptibility to ASF^28^ in comparison with domestic pigs from a commercial farm. Additionally, the microbial communities from indigenous pigs from Africa were compared with both SPF and commercial pigs to analyze the environmental effect on this animal species (*Sus scrofa*). Secondly, our goal was to study the microbiota composition of ASF-resistant warthogs (*Phacochoerus africanus*). The fecal microbiota composition of both wild warthogs and animals raised in a confined space, from the Barcelona Zoo, were analyzed to shed light on the differences acquired within this species when raised under different environment conditions. Furthermore, the core microbiota of ASF-resistant warthogs was determined and compared with the microbiota of susceptible animals in order to investigate the microorganisms that might be involved in disease resistance.

## Results

### The fecal bacterial community in SPF and indigenous pigs from Africa

The microbiota composition was analyzed from fecal samples obtained from domestic adult pigs (*Sus scrofa*) raised under different conditions. Fecal samples from SPF pigs (n = 11) were obtained from pure Large-White pigs raised in ANSES biocontainment facility (French Agency for Food, Environmental and Occupational Health and Safety, Ploufragan, France), while samples from domestic (pure Large-White) commercial pigs (COM pigs, n = 9) were obtained from a PRRSV-free conventional farm from France with a high health status. The feed for both SPF and COM pigs was composed of cereals with addition of a mix of minerals and vitamins, free of anti-parasites or antibiotics. Fecal samples from African pigs (AFR pig) were obtained from a backyard farm in Kenya (n = 15).

All the fecal samples were subjected to DNA extraction and 16S rRNA gene sequencing. A total of 8,172,478 sequences were obtained from fecal samples from different animals sequenced individually. A median number of reads per sample obtained after processing was 208,098 (range 21,134–523,022) with a mean sequence length of 451 nt (Supplementary Table S1), corresponding to 88.5% of the total reads. Operational taxonomic units (OTUs) were identified by clustering sequences at 97% sequence homology.

**Figure 1:**
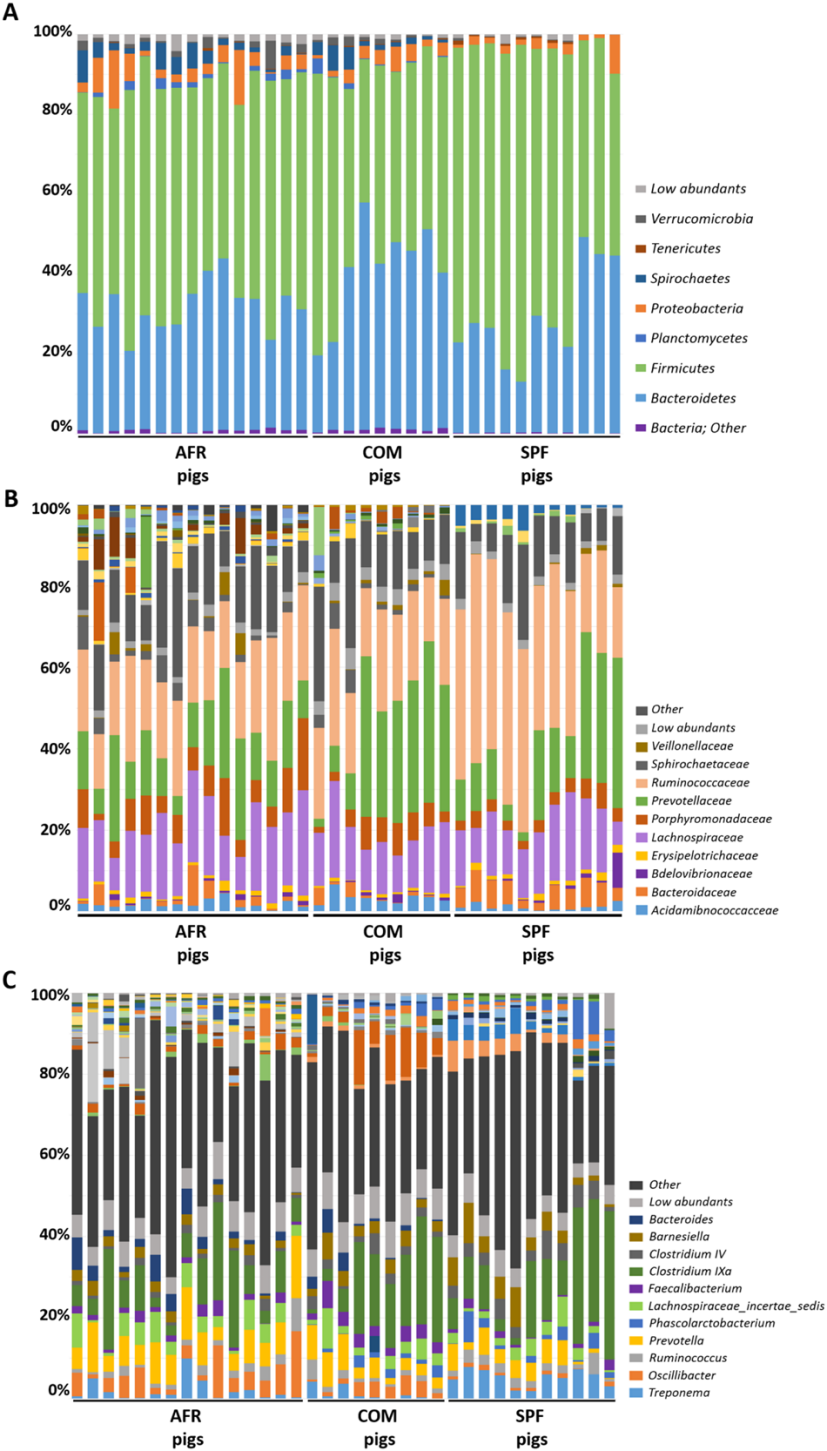
GIT microbiota composition of domestic pigs (*sus scrofa*) obtained from Africa (AFR pig), from a conventional commercial farm (COM pig) and from SPF facilities (SPF pig). The mean relative abundance (%) of OTUs found in feces is presented. Each graph represents the OTUs at different taxonomical levels: phylum (**A**), family (**B**), genus (**C**). Only the ten most relatively abundant OTUs are shown in the legend.

SPF pig’s microbiota was composed of three main Phyla (>1% relative abundance), where *Firmicutes* (66.93%), *Bacteroidetes* (29.10%) and *Proteobacteria* (2.41%), represented 98.44% of the total composition (Fig. 1A; Table 1). Interestingly, *Ruminococcaceae* within the phylum *Firmicutes*, was the most relatively abundant family in SPF pig feces, representing 36.89% of all the sequences found at the family level, which was higher than in both AFR (20.41%) and COM pigs (21.42%). Within *Firmicutes*, *Lachnospiraceae*, represented 14.10% of the assigned families (Fig. 1B, Table 1). From *Bacteroidetes*, *Prevotellaceae* was the most relatively abundant (17.59%), followed by *Bacteroidaceae* (5.18%), and *Porphyromonadaceae* (3.45%). Notably, *Rikenellaceae* (2.73%), from *Bacteroidetes*, represented a unique family that was specific for SPF pigs. Remarkably, many families found in AFR and COM pigs, were completely absent in SPF pigs, these included *Flavobacteriaceae*, *Lactobacillaceae*, *Planococcaceae*, *Sphingobacteriaceae*, *Spirocaetaceae*, among others. The unassigned sequences represented only 12.40% of sequences at the family level.

**Table 1:** Relative abundance of OTUs found the fecal microbiota of the different animals analyzed throughout this study.

*Genera*	Relative abundance (%)					P value*
	SPA Warthog	AFR Warthog	AFR pig	COM pig	SPF pig	
Family	Relative abundance (%)					P value*
	SPA Warthog	AFR Warthog	AFR pig	COM pig	SPF pig	
Genera	Relative abundance (%)					P value*
	SPA Warthog	AFR Warthog	AFR pig	COM pig	SPF pig	
*Bacteroidetes*	23.96	36.85	31.77	40.11	29.10	<0.05
*Firmicutes*	60.06	51.95	54.71	50.65	66.93	<0.01
*Proteobacteria*	6.94	3.18	5.44	3.12	2.41	<0.05
*Spirochaetes*	4.16	2.64	3.56	2.47	0.00	<0.001
*Tenericutes*	2.20	0.26	0.09	0.13	0.36	<0.001
*Verrucomicrobia*	0.22	2.04	1.08	0.90	0.18	<0.001
*Acidaminococcaceae*	0	5.06	1.79	3.28	1.07	<0.001
*Anaeroplasmataceae*	1.18	0	0	0	0	<0.05
*Bacillaceae 1*	0	0.24	1.41	0	0	<0.001
*Bacteroidaceae*	2.84	0	2.03	1.2	5.18	<0.001
*Bdellovibrionaceae*	1.92	0.58	0.49	0.57	1.15	<0.01
*Enterobacteriaceae*	0	0	2.83	0	0	<0.001
*Erysipelotrichaceae*	1.58	2.11	1.11	0.61	1.24	<0.001
*Flavobacteriaceae*	0.72	1.64	1.16	0.91	0	<0.001
*Lachnospiraceae*	16.56	19.93	16.73	14.13	14.1	<0.05
*Lactobacillaceae*	0	0	1.21	0.5	0	<0.001
*Marinilabiaceae*	0	1.61	0.39	0.7	0	<0.001
*Peptostreptococcaceae*	1.84	0.56	0.26	0	0.32	<0.05
*Planctomycetaceae*	0	0.95	1.09	0.73	0	<0.001
*Planococcaceae*	0	0.41	0.51	1.36	0	<0.005
*Porphyromonadaceae*	4.78	2.63	7.63	5.07	3.45	0.54
*Prevotellaceae*	9.68	14.61	14.25	24.04	17.59	<0.001
*Rhodospirillaceae*	1.8	0	0	0	0	<0.001
*Rikenellaceae*	0	0	0	0	2.74	<0.001
*Ruminococcaceae*	21.72	13.88	20.41	21.42	36.89	<0.001
*Sphingobacteriaceae*	0	0	0.45	1.51	0	<0.001
*Spirochaetaceae*	4.16	2.64	3.06	2.44	0	<0.001
*Subdivision5_genera_incertae_sedis*	0	2.02	0.45	0.6	0	<0.001
*Veillonellaceae*	0	0.79	1.37	1.01	0.43	0.63
*Verrucomicrobiaceae*	0	0	1.03	0	0	<0.001
*Acetanaerobacterium*	0.42	0	0	0.97	2.68	<0.001
*Akkermansia*	0	0	1.02	0.23	0	<0.001
*Alistipes*	0	0	0	0	2.73	<0.001
*Alloprevotella*	0	0.81	1.51	6.72	0	<0.001
*Anaeroplasma*	1.18	0	0	0	0	<0.001
*Bacillus*	0	0.24	1.41	0	0	<0.001
*Bacteroides*	2.84	0	2	1.2	5.18	<0.001
*Barnesiella*	1.18	1.28	5.31	2.6	0.78	<0.001
*Clostridium IV*	2.06	0	2.08	1.62	2.22	<0.001
*Clostridium XI*	1.84	0.56	0.26	0	0.32	<0.05
*Clostridium XlVa*	6.42	7.01	7.81	4.89	3.79	0.1
*Escherichia/Shigella*	0	0	2.71	0	0	<0.001
*Faecalibacterium*	0	1.31	0	1.4	2.07	<0.05
*Lachnospiracea_incertae_sedis*	2.24	1.93	4.3	3.71	2.72	<0.1
*Lactobacillus*	0	0	1.2	0.49	0	<0.001
*Oscillibacter*	0	0.96	2.17	3.29	4.64	<0.001
*Paludibacter*	1.56	0	0.67	0.35	0	<0.001
*Paraprevotella*	1.68	3.03	0.71	1.12	1.2	<0.001
*Phascolarctobacterium*	0	4.92	1.66	3.25	1.05	<0.001
*Prevotella*	7.34	10.08	9.89	15.12	16.03	0.58
*Roseburia*	1.4	3.54	0.53	1.12	2.54	<0.01
*Ruminococcus*	4.34	1.9	1.33	2.32	3.84	<0.001
*Rummeliibacillus*	0	0	0.11	1.32	0	0.57
*Saccharofermentans*	0	1.25	0	0	0	<0.001
*Sphaerochaeta*	1.08	0	0	0.56	0	<0.001
*Subdivision5_genera_incertae_sedis*	0	2.02	0.45	0.6	0	<0.001
*Treponema*	3.08	2.36	2.9	1.89	0	<0.001
*Vampirovibrio*	1.92	0.59	0.49	0.57	1.15	<0.05

*P values were obtained perfoming Kruskal-Wallis test*, P value* < 0.05 means at least one of the mean abundances between the groups is different.

Six main phyla were found in AFR pigs feces, where *Firmicutes* (55.64%) and *Bacteroidetes* (31.14%) were the most relatively abundant representing 86.78% of the total composition (Fig. 1A, Table 1). *Proteobacteria* (4.87%) was the third most abundant phylum followed by *Spirochaetes* (3.06%), *Verrucomicrobia* (1.53%) and *Planctomycetes* (1.09%). At the phylum level, unassigned sequences (designated as ‘other’) represented less than 1% of the total sequences. Among the families identified, *Ruminococaceae* family (from *Firmicutes*) was the most relatively abundant, as in SPF pigs, representing 20.41% of all the assigned families (Fig. 1B). Also within the phylum *Firmicutes*, *Lachnospiraceae* was highly abundant representing 14.13%*. Prevotellaceae* was the most abundant within the *Bacteroidetes* phylum for AFR pigs, with 14.25% of relative abundance. Remarkably, within this phylum, the *Porphyromonadaceae* family was found in high abundance in AFR pigs (7.62%). Curiously, the family *Verrucomicrobiaceae*, was only found in these pigs. The percentage of unassigned sequences at family level was 14.76%.

The most relatively prevalent genus found in AFR pigs was *Prevotella* from *Prevotellaceae* (*Bacteroidetes*) comprising 9.89% of all the assigned genera (Fig. 1C, Table 1), although in lower abundance than in SPF (16.03%) or COM pigs (15.12%). Within the *Ruminococcaceae* family, *Oscillibacter* was the most relatively abundant in all groups (2.17%, 3.29% and 4.64% in AFR, COM and SPF pigs, respectively), however many differences in composition were found depending on the group. For instance, the feces from SPF animals showed a higher relative abundance of *Ruminococcus* (3.84%), when compared to COM (2.32%) or AFR (1.33%) pigs. Interestingly, some genera found in SPF pig microbiota, were completely absent or undetected in AFR pigs, such as *Faecalibacterium* and *Acetanaerobacterium*. Among the genera specific for each group, we also identified *Alistipes* in SPF pigs, while *Bacillus* and *Escherichia/Shigella* were specific for AFR pigs. No genus, with relative abundance higher than 1%, was specific of COM pigs. Moreover, several genera were found more relatively abundant in SPF pigs than in AFR pigs, including *Bacteroides* (5.18%) and *Roseburia* (2.54%). Conversely, other genera were found less abundant than in AFR pigs such as *Clostridium XIVa* (3.79%), *Lachnospiracea incertae sedis* (2.72%) and *Barnesiella* (0.78%) or were even undetected, such as the potentially pathogenic *Treponema* and *Alloprevotella*. Noteworthy, many genera present in AFR pigs were completely absent or in low abundance in SPF pig feces (Table 1, Fig. 1C). Unfortunately, a significant percentage of the sequences could not be confidently assigned to specific genus (36.59%, 37.15% and 34.26% for AFR, SPF and COM pigs, respectively) and additional analyses should be done to better characterize them.

### The fecal bacterial community in warthogs (*Phacochoerus africanus)*

African warthog (AFR warthog) feces were obtained by Kenyan wildlife service (KWS) through visual identification of the animals and collected just after deposition (n = 14). Moreover, samples from warthogs (n = 5) born and raised in the Barcelona zoo from Spain (SPA warthogs) were also included in the analysis. The raw reads obtained were 13,295,340 for this group.

**Figure 2:**
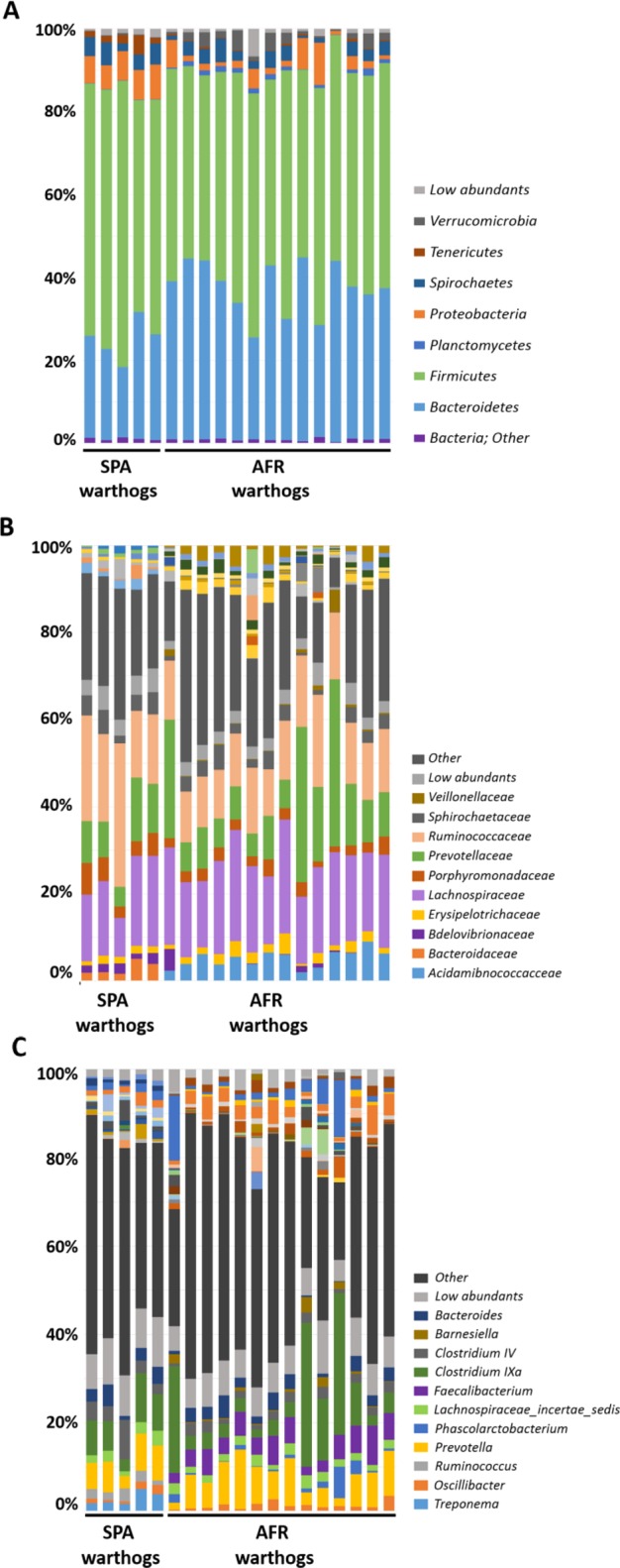
GIT microbiota composition of warthogs *(Phacochoerus africanus)* coming from Africa (AFR warthog) and from captive animals from Barcelona (Spain) zoo (SPA warthog). The mean relative abundance (%) of OTUs found in feces is presented. Each graph represents the OTUs at different taxonomical levels: phylum (**A**), family (**B**), genus (**C**). Only the ten most relatively abundant OTUs are shown in the legend.

The warthog fecal microbiota was comprised of five main phyla (>1% relative abundance) both in wild (AFR warthogs) and captive animals (SPA warthogs). *Firmicutes* and *Bacteroidetes* were the most abundant phyla, representing 88.80% of the total microbiota composition (Fig. 2A, Table 1) in AFR warthogs, and 84.02% in SPA warthogs. *Proteobacteria* was the third most abundant phylum in both groups, although with significant differences in its relative abundance (6.94% SPA and 3.18% AFR warthogs). The relative abundance of the *Spirochaetes* phylum was almost doubled in the SPA warthogs (4.16%) in comparison with African animals (2.64%). The fifth phylum was different depending on the group, being *Tenericutes* (2.20%) for SPA warthogs and *Verrucomicrobia* (2.04%) for AFR warthogs.

Within *Firmicutes*, the *Lachnospiraceae* family was the most abundant in AFR warthogs, representing 19.93% of all the families found, while it represented 16.56% for SPA warthogs (Fig. 2B). The most abundant family found in SPA warthogs was the *Ruminococcaceae* family from *Firmicutes* with 21.72%, which represented only 13.88% of all the OTUs assigned at family level in AFR warthogs. *Prevotellaceae* was the most abundant family within the *Bacteroidetes* phylum for AFR warthogs, with 14.61% of relative abundance, while it represented 9.68% in SPA warthogs. Within this phylum, the *Porphyromonadaceae* family was more abundant in captive warthogs than in wild animals from Africa (7.62% and 2.63% for SPA and AFR warthogs, respectively). Many families present in AFR warthogs were absent or in low abundance in SPA warthogs, such as *Acidaminococcaceae* (5.06%, AFR warthogs), *Marinilabiaceae* (1.61%, AFR warthogs), and *Subdivision5 genera incertae sedis* (2.02%, AFR warthogs); while others showed the opposite tendency, such as *Anaeroplasmataceae* (1.18%, SPA warthogs), *Bacteroidaceae* (2.84%, SPA warthogs) *Peptostreptococcaceae* (1.84%, SPA warthogs) and *Rhodospirillaceae* (1.8%, SPA warthogs). The percentage of unassigned sequences at family level was similar for both groups, being 24.22% for AFR warthogs and 24.62% for SPA warthogs.

The genera (Fig. 2C, Table 1) exhibiting the highest relative abundance were the same in both groups, with *Prevotella* as the most relatively abundant (10.08% in AFR and 7.34% in SPA warthogs) and *Clostridium IVa*, as the second most relatively abundant in both cases (7.01% for AFR and 6.42% in SPA warthogs). Interestingly, *Phascolarctobacterium*, the third most abundant genus in AFR warthogs (4.92%), was not detected in captive animals (SPA). Some abundant genera within the AFR warthog fecal population were in lower abundance in SPA warthog feces, such as *Faecalibacterium* (1.31%) and *Saccharofermentans* (1.25%). By contrast, several genera found in SPA warthogs were absent in their African counterparts, e.g. *Anaeroplasma* (1.18%), *Bacteroides* (2.84%), *Clostridium IV* (2.06%), *Paludibacter* (1.56%), *Ruminococcus* (4.34%) and *Sphaerochaeta* (1.08%).

Unfortunately, a significant percentage of the sequences could not be confidently assigned to a specific genus (43.04% and 45.5% for AFR and SPA warthogs respectively) and require further characterization.

### Diversity analysis

The diversity in the fecal microbiota of each group was estimated and compared. Rarefaction at maximum depth was done to measure evenly the richness within each group of animals. The mean observed species at this depth was of 2,396 for AFR warthogs, 2,218 for AFR pigs, 1,667 for SPA warthogs 2,183 for COM pigs and 1,764 for SPF pigs. In all samples, the plateau was reached at the maximum depth, meaning that the sampling procedure was adequate.

**Figure 3:**
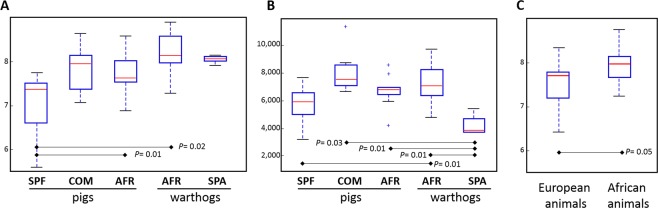
Alpha diversity on rarefied fecal samples from pigs (AFR, COM and SPF) and warthogs (AFR and SPA). Alpha diversity was compared between groups by measuring the Shannon-Wiener’s metrics (**A**). Species richness was computed through Chao1 index (**B**). Shannon-Wiener’s metrics were also calculated for African animals grouped together (AFR pigs and AFR warthogs) against European animals (SPA warthogs, COM and SPF pigs (**C**). Dotted lines represent the standard deviation Outliers are indicated with plus signs on the plot.

Analysis of alpha diversity metrics, using Shannon’s index showed the lowest diversity in the fecal microbiota of SPF pigs, which was statistically significant when compared with AFR warthogs and AFR pigs (non-parametric test 999 permutations, *P* < 0.05, Fig. 3A). Remarkably, the microbiota of AFR warthogs resembled the microbial diversity and the richness estimated through the Chao index (Fig. 3B), found in the domestic pigs (AFR pigs). Worth to mention, is the observation that SPA warthogs showed the same diversity in their feces as the wild counterparts (Fig. 3A), but the least richness together with SPF pigs when compared with the rest (Fig. 3B, Supplementary Table S2). The feces from African wild and domestic animals grouped together were the most diverse (Shannon *P value* = 0.05) when compared against all other groups (Fig. 3C).

**Figure 4:**
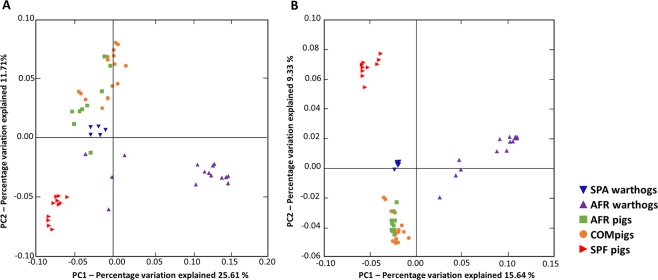
Principal Coordinate Analysis plots (jackknifed) representing beta diversity on rarefied samples. Beta diversity of fecal samples from pigs (AFR, COM and SPF) and warthogs (AFR and SPA) was computed through weighted (A) and unweighted (B) UniFrac analyses.

To understand the differences across the fecal microbiota of different samples, the beta diversity was estimated. Weighted and unweighted UniFrac phylogenetic distances were used to generate the beta diversity distance matrices and calculate the degree of differentiation among the samples. Samples were grouped to test the factors leading to clustering and principal coordinate analysis was done on each group. Resampling was performed repeatedly on a subset of the available data for each sample evenly (jackknifing) to measure the robustness of individual clusters (Fig. 4). Analysis of similarities was performed with a nonparametric statistical test (ANOSIM) and R was estimated based on the difference mean ranks between groups, where R = 0 indicates completely random grouping. The mean distances between all the groups were calculated and showed to be statistically different in both weighted (Fig. 4A; R = 0.6785, *P* = 0.001) and unweighted analyses (Fig. 4B; R = 0.9034, *P* = 0.001), showing both a quantitatively and qualitatively different composition of the microbiota according to the groups. Distance-based redundancy analysis (db-RDA) was performed to explore the clustering of the samples in an ordination plot when they were divided in two groups: resistance warthogs (SPA and AFR) or susceptible pigs (SPF, COM and AFR) animals (Supplementary Fig. S3). The db-RDA diagram depicting the community structure revealed a cluster of warthogs apart from pigs, where the greatest amount of variation was explained by the explanatory variable ‘resistance-to-ASF’ (*P* < 0.05).

### Description of the ASF resistant and susceptible core microbiota

Many differences were detected in the fecal microbiota composition of the different groups analyzed throughout this study. However, one of the main objectives of this study was to identify particular OTUs differentially present in feces of ASF-resistant animals in comparison with susceptible ones. Since some bacteria found in feces could be in transit through the animal, but not representative of a stable bacterial community, a core analysis was made. The core was defined as those OTUs present in all the samples from either warthogs (AFR and SPA warthogs, resistant core) or pigs (COM and SPF pigs, susceptible core) with the aim of uncovering the main differences of permanent inhabitants of the gut from animals with different susceptibilities to ASF.

**Figure 5:**
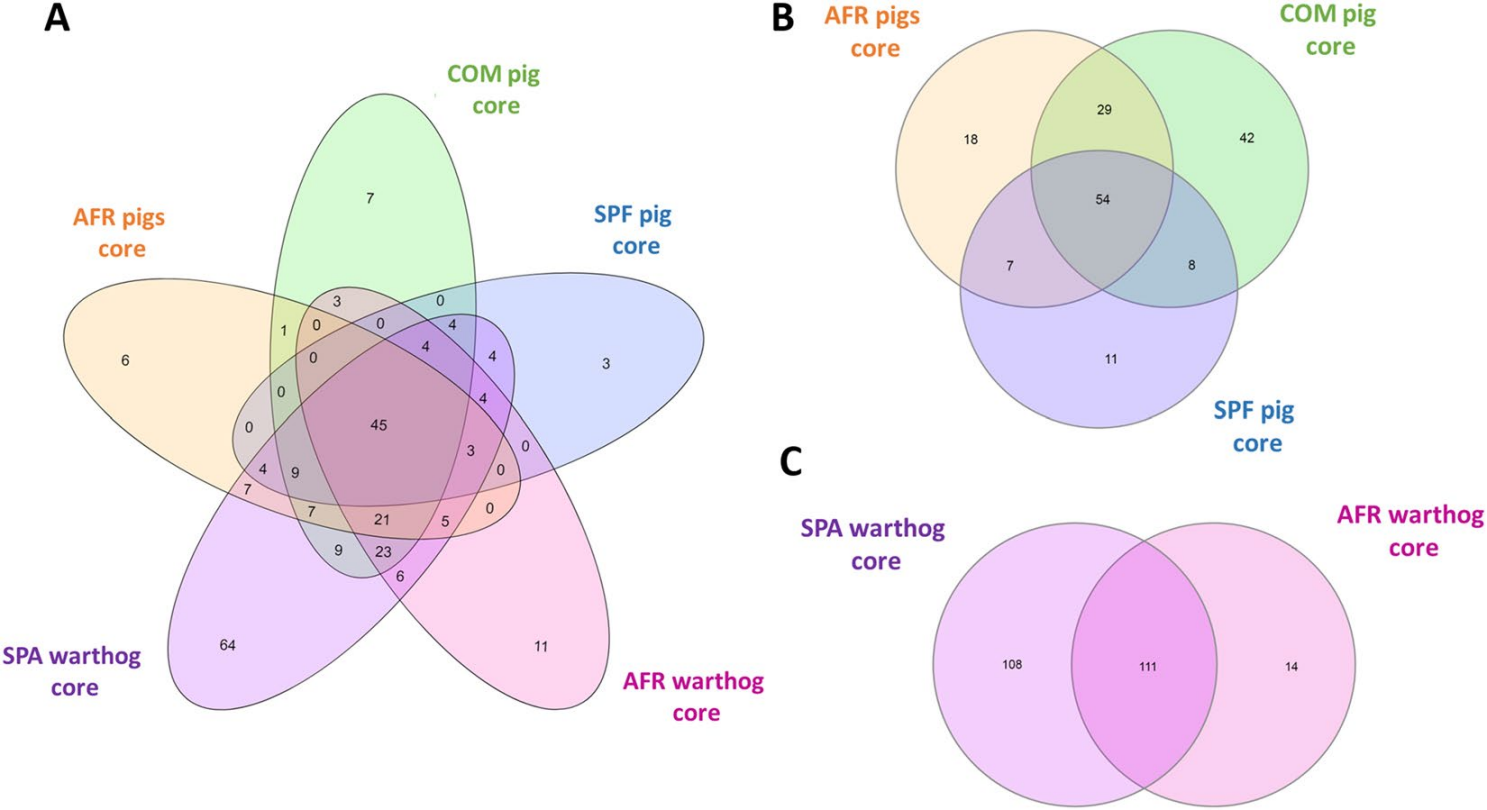
Venn diagram showing the number of shared OTUs among the core communities of all groups of wild and domestic pigs.

The Venn diagrams showing the number of OTUs found for each core are depicted together in Fig. 5A. Additionally, subgrouping comparisons are shown in Fig. 5B,C. The SPF pig core exhibited the lowest number with 81 OTUs, while the AFR pig core was composed of 109 OTUs and the COM pig core comprised 136 OTUs, as depicted in Fig. 5B. The susceptible core, composed of 54 OTUs, was considered as the intersection between the OTUs present in COM, SPF and AFR pig cores. The resistant core, defined as the OTUs present in all the samples from both AFR and SPA warthogs, consisted of 111 OTUs, as shown in Fig. 5C. The resistant and susceptible cores shared 45 OTUs. From the 111 OTUs present in the resistant core, 93 OTUs are shared with COM pigs, 56 OTUs are present in the SPF core and 74 OTUs in the AFR pig core, leaving 6 OTUs exclusively within the resistant core (Table 2). Likewise, from the 54 OTUs of the susceptible core, 9 OTUs are shared with the SPA warthog core, while absent in the AFR warthog core. Moreover, there are 7 OTUs exclusively present in the COM pig core, and only 3 OTUs were exclusively present in the ASFV highly-susceptible core from SPF animals. The full list of OTUs from each of the five cores is presented in Supplementary Tables S4 and S5. The mean relative abundances of the 6 OTUs exclusively from the resistant core, are shown in Table 2.

**Table 2:** Mean relative abundance (%) of the OTUs exclusively found in the core microbial composition of feces from AFSV resistant animals.

Out						Mean relative abundance (%)					Mean relative abundance Group (%)		*P value*
Domain	Phyla	Order	Class	Family	Genus	SPA warthog	AFR warthog	SPF pig	COM pig	AFR pig	Resistant warthogs	Susceptible pigs	
*Bacteria*	*Bacteroidetes*	*Bacteroidia*	*Bacteroidales*	*Porphyromonadaceae*	*Paludibacter*	1.555	0.287	0.000	0.362	0.658	0.921	0.340	<0.05
*Bacteria*	*Tenericutes*	*Mollicutes*	*Anaeroplasmatales*	*Anaeroplasmataceae*	*Anaeroplasma*	1.191	0.194	0.241	0.095	0.089	0.692	0.141	<0.05
*Bacteria*	*Bacteroidetes*	*Bacteroidia*	*Bacteroidales*	*Porphyromonadaceae*	*Petrimonas*	0.382	0.090	0.000	0.000	0.002	0.236	0.001	<0.05
*Bacteria*	*Proteobacteria*	*Gammaproteobacteria*	*Pseudomonadales*			0.023	0.580	0.003	0.002	0.022	0.301	0.009	<0.05
*Bacteria*	*Proteobacteria*	*Gammaproteobacteria*	*Pseudomonadales*	*Moraxellaceae*		0.020	0.578	0.002	0.001	0.021	0.299	0.008	<0.05
*Bacteria*	*Proteobacteria*	*Gammaproteobacteria*	*Pseudomonadales*	*Moraxellaceae*	*Moraxella*	0.006	0.294	0.000	0.000	0.001	0.150	0.000	<0.05

### Functional prediction

**Figure 6:**
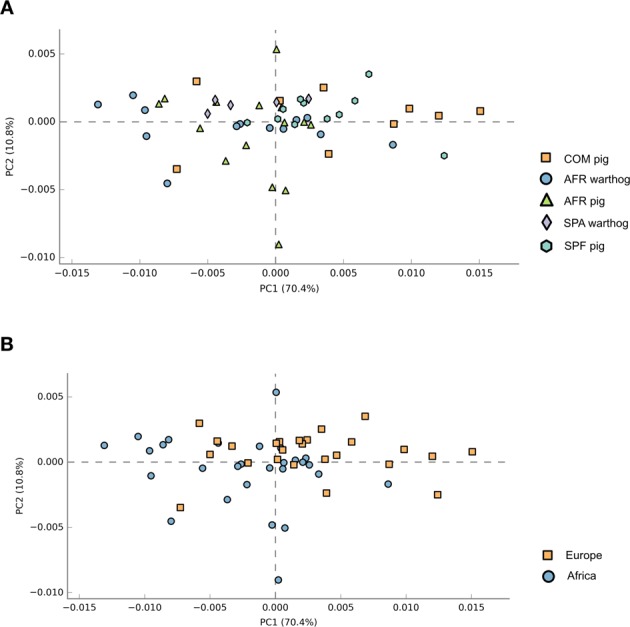
Principal component analysis illustrating distances between predicted sample pathway profiles. PC1 accounts for 70.4% and PC2 10.8% of the variation in the PICRUSt predicted pathways. Samples are colored by either the pig group they belonged to (**A**) or by continent of origin (**B**).

**Figure 7:**
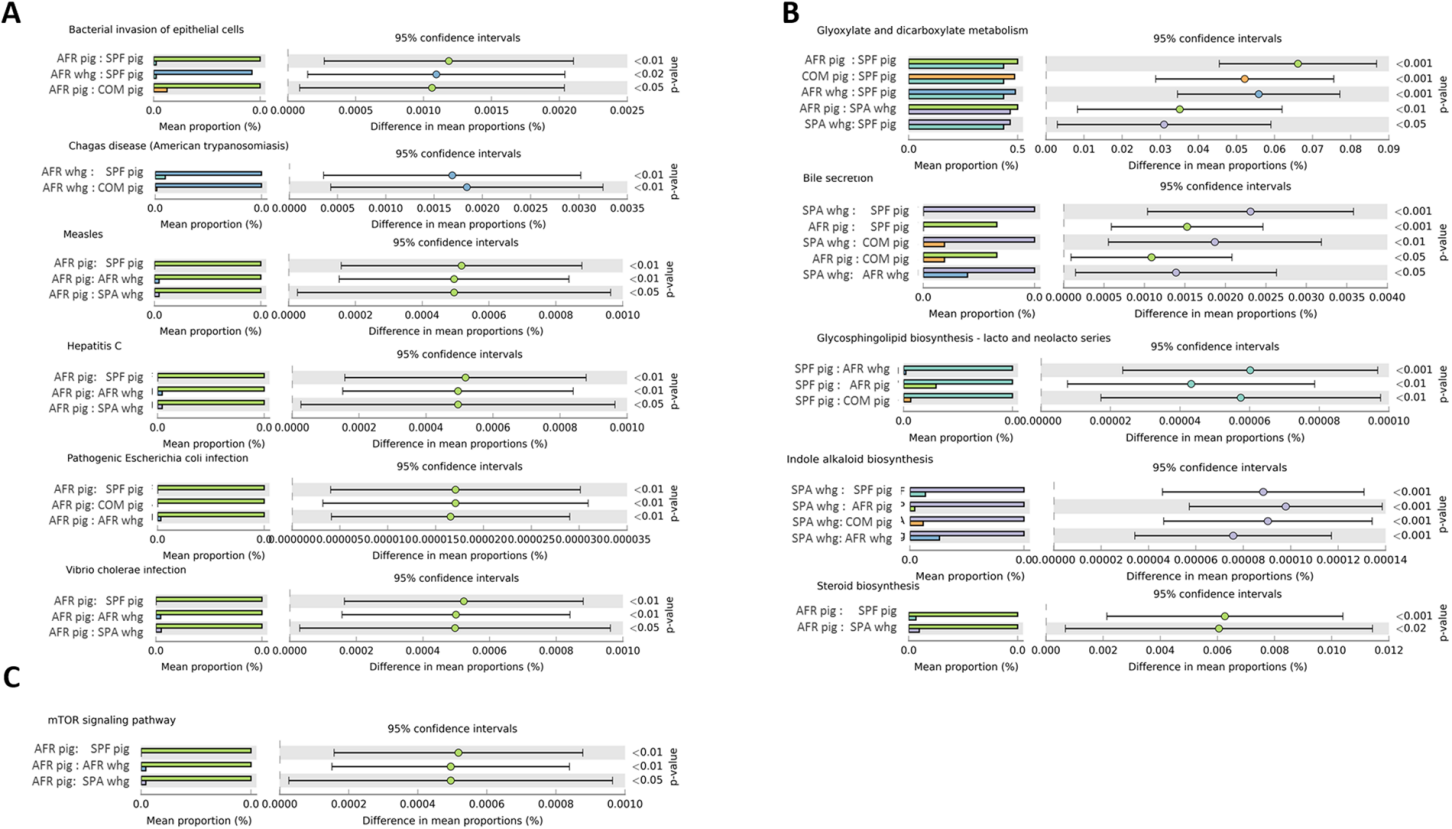
Prediction of changed KEGG pathways using PICRUSt analysis. Selected pathways found to be significantly different among the different animal groups are depicted based on their relation to infection diseases (**A**), metabolic pathways (**B**) and immune response (**C**). Dot plots on the right show the differences in mean proportions between the two indicated groups using *P* values. All KEGG pathways that were significantly changed are detailed in Supplementary Table S6.

The community’s functional capabilities were predicted using PICRUSt (based on the 16rRNA gene data) to explore the function of gut bacteria and better characterize the potential role exerted by the microbiota of the different groups under study. Principal component analysis (PCA) was performed in order to evaluate the taxonomic profile differences among the microbial communities of each group (Fig. 6). When samples were analyzed according to the animal group they belonged (COM, SPF, AFR pigs or SPA, AFR warthogs) showed good clustering (Fig. 6A, PERMANOVA R^2^ = 0.2347, *P* = 0.001). However, the highest percentage of the variability was explained by grouping samples by the continent of origin, as depicted in Fig. 6B (PERMANOVA R^2^ = 0.4242, *P* = 0.001). Pathway analysis was conducted using Kyoto Encyclopedia of Genes and Genomes (KEGG) pathways at three different levels. The level 1 KEGG pathways showed a predominant proportion of significant predicted functions linked to Metabolism pathways (48.5%) followed by Organismal Systems (13.2%) together with Human diseases (13.2%), and Environmental Processes (9.3%). Within these four most abundant pathways, we selected those pathways related to either infectious diseases, nutrition or immune response showing statistically significant differences. These active features at the level 2 KEGG pathway categories corresponded to Infectious Diseases, representing the 5.6% from the total of the active features, Signal Transduction (4.0%), Biosynthesis of Other Secondary Metabolites (5.3%), and Digestive System (2.6%). We focused on representative active pathways at the level 3 KEGG pathway categories that showed statistically significant differences within these active functions (Fig. 7). KEGG functional predictions related to infectious diseases showed significant differences between the AFR pigs or AFR warthogs when compared with the rest of the groups (Fig. 7A). Five metabolic pathways related to nutrition were found to be statistically different (Fig. 7B). KEGG gene families associated to glyoxylate and decarboxylate were significantly diminished in SPF pigs compared to all the other groups. Regarding the families associated with bile secretion, both samples from warthogs and AFR pigs were more highly represented than the rest. Moreover, those families related to glycosphingolipid biosynthesis had marked differences between SPF pigs compared with all other groups. The indole alkaloid biosynthesis pathway was higher in SPA warthogs, while the steroids biosynthesis was higher in AFR pigs when compared with SPF pig and SPA warthogs (Fig. 7B). Furthermore, the mTOR signaling pathway related to immune response (Fig. 7C) was higher in AFR pigs compared with both AFR and SPA warthogs, and SPF pigs.

## Discussion

Once a healthy gut microbial community has developed, it will be quite stable through life. The GIT microbiota is shaped as the diet becomes more complex and the immune-system matures leading to different digestibility capacities^29^. Many studies have demonstrated that factors, such as locality and habitat, are important in the microbial community structure, because variables like local flora and fauna, photoperiod, available food and climatic conditions may affect host microbiota^30,31^. The definition of a healthy microbiome is important since its maintenance is recognized as a major factor in animal health^32^, development^33^ and productivity^34,35^.

In this work, the microbiota of wild African warthogs and SPF pigs was deeply studied for the first time, unravelling many interesting differences when compared with both African domestic pigs and European warthogs raised in confined conditions in the zoo. Although there are many reports describing the GIT microbiota of domestic pigs, we decided to include this group in the analysis under the same experimental and *in silico* procedures for comparison. The variations found can be explained by genetic differences between animal species and/or the environmental conditions under which the animals were raised, including diet, antibiotic treatments or pathogen exposure. Therefore, when comparing groups where genetic differences are minimized, the environmental conditions, including diet, management and antibiotic treatments, become the main responsible of the differences in microbial diversity observed. AFR warthogs showed high diversity and richness, while their counterparts subjected to a completely different environment, SPA warthogs, showed lower richness but similar diversity. With many taxa missing from SPA warthogs, such as *Acidaminococcaceae*, *Marinilabiaceae*, *Planctomycetaceae*, *Veillonaceae*, some others were increased to relative abundances near to the values seen in COM pigs, probably related to similarity in the diets of these groups (e.g. *Ruminococcaceae*, *Porphyromonadaceae*, *Bacteroidaceae*). Importantly, the relative abundance of *Prevotella* was higher in wild warthogs when compared with the captives from the zoo, in agreement with a previous report^27^. Hence, the increase detected in other microbial families could be to counteract the loss of this beneficial genus, a hypothesis that need further confirmation.

Other important difference was revealed when analyzing SPF pig microbial composition. As expected, the microbiota diversity in SPF pigs exhibited a different scenario than in COM pigs. *Firmicutes* had the highest abundance in the microbiota within all the groups tested, suggesting that the reduction of pathogenic bacteria from other phyla, is probably fulfilled, at least in part, by this phylum. The same tendency was also observed in AFR warthogs, probably indicating that some bacteria within *Firmicutes* might be markers of a healthy gut microbiota. However, this hypothesis needs confirmation. Noteworthy, the family *Porphyromonadaceae* was observed in low amount in the SPF microbiota. This observation is potentially important given that the SPF microbiota was generally less diverse and rich. The lower relative abundance of this family therefore translates to drastic reduction in absolute numbers. This family should be investigated further, since the loss of members of the microbiota in SPF animals might be involved in the impaired functions or increased disease susceptibilities seen in this animal group^13^. *Allistipes* was found within the distinct genera found in SPF pigs. Interestingly, high abundances of this genus have been associated with autoimmune disorders^36,37^. SPF pigs lacked many other genera present in their commercial (non-SPF) counterparts, such as *Treponema* considered as the main genus driver of the enterotype-like previously defined for pigs^38^. Moreover, the lack of *Treponema* in this group might be associated with the high abundance of *Ruminococcus* in SPF pigs, since it is accepted that both *Ruminococcus* and *Treponema* are involved in the same cellulose and lignin degradation pathways^39^. The loss of *Lactobacillus* in the SPF pigs may be significant, as it is considered as a beneficial genus for pig gut health^40^. Moreover, the loss in richness and diversity is in agreement with the impaired immune system development^41^. Other interesting findings showed that potential pathogenic bacteria (for domestic pigs) are more often present in pigs in contrast to warthogs. For instance, the family *Enterobacteriaceae*, present only in pigs from Africa, are considered a major source of foodborne diseases in human^42^ and also cause different diseases in pigs^43^. The presence of this family is particular to the individual^44^ and it has proven to be absent from the core microbiota from the swine gut^45^.

When comparing AFR and COM pigs many aspects may be important. All the samples analyzed clustered together when analyzed using PCoA, meaning that many members of the fecal microbiota are common to these pigs, irrespective of the habitat they live (i.e. *Lactobacillus*). COM pigs showed higher richness, but this might represent an artifact of having higher numbers of unidentified OTUs in the AFR animals. This idea is supported by the fact that no frequently observed OTU (>1% relative abundance) was present in COM animals and absent in AFR pigs, while the opposite was observed in several cases; that is the case of *Bacillaceae*, *Enterobaceriaceae* and *Peptostreptococcaceae*. These three OTUs should be further studied as they might be involved in the increased resistance to ASFV infection of AFR pigs reported elsewhere^46^.

We also found many differences between the two different animal species analyzed from Africa, AFR warthogs and AFR domestic pigs, which formed two distinct clusters according to PCoA (Fig. 4). Since both species share similar environment (African domestic pigs are free-ranging animals), the differences observed could probably reflect their genetic differences. Further studies should be needed to confirm this hypothesis. Although at phylum level there are no major differences between these two groups, when analyzing families many differences were present. For instance, *Bacteroidaceae*, a family that contains both commensal and pathogenic bacteria, was absent in AFR warthogs although present in African pigs (as in COM pigs). Among the bacteria belonging to this family, *Bacteroides fragilis* is a pathogen that has been associated with diarrheal disease in young pigs^47^, and seems to be colonizing specifically colonizing domestic *Sus scrofa*. The *Prevotellaceae* family, known to contain the most important primary fermenters^48^, is present in the feces from African warthogs and pigs at the same percentage, probably related to the digestion of complex carbohydrates derived from plants that this microbial network is involved in processing. The *Ruminococcaceae* family was present in lower abundance in AFR warthogs. The lower abundance of this family representing anoxic fermentative digestive tracts like rumen and large intestine of animals^42^ is, at least partially, due to the diminished abundance of the genus *Ruminococcus*. Another important genus that has been associated with many beneficial roles^40^, *Lactobacillus*, was detected only in domestic pigs, although in higher abundance in the African animals (AFR pigs) analyzed. Diet may also play a role in this finding, since African pigs are not cereal-fed to the same extent as commercial pigs, and many times are fed with household organic waste^48^. Unfortunately, the diet on this group was not available so the conclusions regarding the microbiota composition should be taken carefully due to potential impact of the different diet^29^ received in this group. Many of the similarities found when comparing AFR warthogs and AFR pigs are important features that unveil the complexity of community structure related to the environment. However, the similarities found between AFR and COM pigs that led to the clustering of these two groups together is perhaps surprising given the differences in environment. The absence of particular families in COM pigs should also be analyzed further. This might be related to the loss of a beneficial role in protection against pathogens in wild species.

Many different clades were identified throughout this study, however only a few predicted categories were found to be statistically different between the groups. Interestingly, when all the samples from Africa were grouped together and compared against all the European ones, two different clusters were created. This suggests that, although the microbial communities might be dissimilar between African animals, the overall functional pathways seem to be conserved. This is in agreement with what was reported in the Human Microbiome Project demonstrating stability of metabolic pathways despite variability of the underlying microbial taxa^49^. Based on large number of reports, it is now widely accepted that the commensal microbiota has a huge impact on the metabolism of the host^11^. Dysbiosis in the GIT has been linked to many alterations in immune responses and in disease development^50^. The fact that we found the mTOR signaling pathway more highly represented in AFR pigs deserves further study as this pathway is important in adaptive immunity and plays a crucial role in keeping the balance between T cell quiescence and activation^51^ which is essential for immune protection^14^. The link between the intestinal microbiome and hepatitis B virus infection^52^, HIV infection^53^ and severity of Malaria in *Plasmodium* infections^54^, has also been demonstrated. The differences among the infectious diseases pathways found in AFR pigs and warthogs when compared to the rest of the groups, suggest these gene families should be further analyzed in order to fully understand the differential disease susceptibility seen in these animals. Moreover, the differences in nutrition-related pathways might reflect the differences in the digestive system and can be useful for understanding the full structure of microbial community composition in these species. All these predictions about the microbiome function should be treated as hypotheses rather than conclusions and need validation through functional assays^55^.

The core analysis of the OTUs present in all the samples from ASFV resistant animals compared with the core elements present in susceptible animals, provided some remarkable findings. Bacterial commensal microbiota might be involved in the resistance to ASF disease, as it has been demonstrated for other viral infections in swine^56,57^. The OTUs found exclusively in resistant animals represent good candidates for testing experimentally. The fact that all the OTUs in the resistant core were occasionally present and in low abundance in animal feces, points out to the importance of characterizing the complete communities inhabiting the GIT. It cannot be ruled out that some more abundant species pertain to the resistant core, as the analysis does not include the unassigned OTUs, which, unfortunately, represent a high number in the warthog microbiota. Several core OTUs present in resistant animals appeared as potential candidates involved in the resistance towards ASF viral disease, and therefore require further investigation. For instance, probiotics from the *Anaeroplasma* genus have shown to improve both weight gain and feed intake, while reducing diarrhea in early-weaned piglets^58^. On the other hand, some of these exclusive genera have been associated with pathology. Within the vast diverse genus *Moraxella*, at least some species have been shown to be pathogenic, such as *M. bovis*^59,60^. Also, *M. porci* and *M. plurianimalium* that have been isolated from systemic lesions^61,62^. For other OTUs, there is controversial data indicating either beneficial or detrimental roles in the pig GIT, which may depend on the specific strain.

In this study, we described many relevant members from the microbiota composition of ASF-resistant animals that deserve further characterization. Nevertheless, it should be stated that, due to both the limitations of the technology used and the scarcity of data related to wild species available in the databases, the complete structure of the GIT microbiota has not been defined, since many taxa remained unidentified especially in warthogs. Moreover, the microbiome is the collection of all host-associated microorganisms including not only the bacterial and archaeal microbiome, but also the mycobiome (fungi), the virome (bacteriophages, eukaryotic viruses) and the meiofauna (unicellular protozoa and helminths)^63,64^, highlighting the importance of expanding future studies to uncover the entire microbiome. Further studies using metagenomics approaches will assist in the characterization of the full structure of the wild and domestic porcine GIT microbiota and unveil the potential novel functional pathways present in wild animals. It would potentially be of interest in the future to analyze the microbiota of African bush pigs (*Potamochoerus larvatus*) which also undergo asymptomatic infections with ASFV, to see if there are similarities with the warthogs, which are phylogenetically more distantly related to domestic pigs *(Sus scrofa)*. To better define the optimal mixtures carrying out precise functions, we are currently characterizing bacterial isolates from warthog feces in greater depth. Meanwhile, we hypothesize, based on the success of fecal transplantation to treat infections^65^ in other species, that an exploration of this therapy as potential treatment for a wide variety of domestic pigs diseases may result in improved pathogen resistance.

## Methods

### Animals and sample collection

Fecal samples from 15 wild African warthogs (*Phacochoerus africanus*) were kindly collected by the Kenyan Wildlife Service (KWS) just after the observation of deposition and visual confirmation of the species of interest in Kenya (GPS coordinates −1.336498, 36.777597). Samples from twelve different African indigenous pigs (*Sus scrofa*) were collected from a specific backyard farm in Kenya (GPS coordinates −1.268028, 36.722050). SPF pig (*Sus scrofa domesticus*) feces were collected from the French Agency for Food, Environmental and Occupational Health and Safety, ANSES in Ploufragan, France. Fecal samples from 12 pure Large-White domestic pigs (*Sus scrofa domesticus*) from the same pen, were obtained from a high-health status commercial farm from France. Both groups of pigs were fed with same composition with the addition of a mix of minerals and vitamins and in the absence of antibiotics and anti-parasites. The food pellets of SPF pigs was additionally treated by heat at 140 °C and kept at 80 °C for 2 minutes, then the temperature was decreased to 15–20 °C. At the end of the process, the humidity was of 12–13%. Aiming to mimic the random collection of feces in the African fields, samples from males and females of different ages (post-weaned) were collected from both conventional and domestic pigs, taking into account that these are factors that do not influence in ASFV susceptibility. Feces from the five adult African warthogs (*Phacochoerus africanus*) born (same litter) and raised in the Barcelona zoo (Spain) were also included in the analysis. Captive warthogs from the zoo were fed with commercial cereal-based feed, but complemented with apples, potatoes and carrots. No antibiotic treatment was used at least three months prior to the collection of feces for any of the captive or domestic animals included in this study.

Samples from all the animals included in this study followed the sample collection procedure follow described. Feces were freshly obtained just after. A portion from the interior area of the fecal sample was collected with a sterile spoon and placed in a sterile tube until they were transported to the laboratory on ice. Upon arrival, 300 mg of fresh feces were stored at −80 °C for further analysis. According to European (Directive 2010/63/EU of the European Parliament and of the Council of 22 September 2010 on the protection of animals used for scientific purposes) and Spanish (Real Decreto 53/2013) normative, feces collection procedure did not require specific approval by an Ethical Committee (Chapter I, Article 1, 5 (f) of 2010/63/EU).

### DNA extraction and 16S rRNA sequencing

Total DNA was extracted from 300 mg of resuspended feces in 900 µl of PBS using the Nucleospin Blood kit (Machinery Nagel). Purified DNA was eluted in a final volume of 50 μl of elution buffer (5 mM Tris, pH 8.5). The quality and quantity of genomic DNA was evaluated on a BioDrop DUO (BioDrop Ltd). The library preparation for sequencing was performed within 24 h after the DNA extraction, at Servei de Genomica, Autonomous University of Barcelona.

### Sequencing and taxonomic analysis

Sequencing of the hypervariable region V3-V4 of 16S rRNA gene was preformed using Illumina pair-end 2 × 250 bp MiSeq platform following the manufacturer instructions (MS-102–2003 MiSeq® Reagent Kit v2, 500 cycle) under the same conditions reported previously^66^. Five different runs with individual barcoding and a maximum of 20 samples/run were used for this study. Each sequence was assigned to its original sample according to its barcode, which was removed from sequences before further processing.

Raw sequences were initially filtered to keep only the high-quality-reads (Q > 25). Paired-end joining was done by using fastq join^67,68^ under default values. Classification of taxonomic abundances was done as previously reported^66^ using the Quantitative insights into microbial ecology^69^ (QIIME) software package (version 1.9). Sequences were clustered into OTUs at 97% similarity using UCLUST algorithm^70^ and the Greengenes database (version 13.8)^71^. Chimeric detection and removal was done with USEARCH 6.1^70,72^ against the ChimeraSlayer reference database^73^. Taxonomic assignment was done with Naïve Bayes classification against RDP database^74^ (Release 11, 2016). For each taxon, the Kruskal Wallis test was perform to compare OTU frequencies in sample groups and to ascertain whether or not there are statistically significant differences between the OTU abundance in the different sample groups, *P* values were FDR-corrected for multiple hypotheses testing. Single rarefaction was done at 21,134 sequences/sample for alpha-diversity analysis. Alpha diversity and richness estimation through Shannon-Wiener and Chao1 indices respectively, were calculated on rarefied data using *alpha_diversity.py* script from QIIME. Alpha diversity between groups was compared through two-sample non-parametric t-tests (Monte Carlo method) at maximum depth in rarefied samples (with 999 permutations). UniFrac weighted and unweigthed distances were calculated to assess significant differences across samples^75,76^. Testing the significant differences between grouped samples was done with *compare_categories.py* script from QIIME^75^ using ANOSIM as the method^77^. The R statistic is calculated by ANOSIM, where an R value near +1 means that there is dissimilarity between the groups, while an R value near 0 indicates no significant dissimilarity between the groups. Samples were considered to be significantly different when the accompanying *P* value was < 0.05. Distance-based redundancy analysis was done to estimate the strength and statistical significance of sample groupings using Euclidean distance matrices.

Functional prediction of the microbiome were done using 16S rRNA gene sequence data using the platform NEPHELE (vs 2.2.2)^78^ which integrates the Phylogenetic Investigation of Communities by Reconstruction of Unobserved States PICRUSt (vs 1.1.3)^24^. The closed reference OTU picking approach was done against Greengenes database (vs 13.8). Then, metagenome predicted functions were inferred using the Kyoto encyclopedia of Genes and Genomes (KEGG)^79^ database. Finally, the Statistical Analysis of Taxonomic and Functional Profiles software^25^ (STAMP vs 2.1.3) was used to both visualize functional prediction and test statistical hypothesis to analyze taxonomic and functional profiles. The analysis was done at level 3 using the unclassified data only for calculating sequencing profiles. Statistical tests to infer the biological relevance of features in the predicted metagenomic profiles between multiple groups were done with Kruskal Wallis^80^ followed by the Tukey-Kramer *post hoc* test. Effect sizes (eta-squared) and confidence intervals were also estimated to provide critical assessment of the biological relevance of test results incorporating the Bonferroni correction (p < 0.05 was considered statistically significant).

## Supplementary information

Supplementary Info

## Data Availability

All the sequences included in this study are available at the NCBI database. SRA Accession number SUB4621742, BioProject PRJNA496331 and BioSamples SAMN10238771-824.
