# Supplementary material for: Comparative analysis of the fecal microbiota from different species of domesticated and wild suids
Source: Sci Rep. 2019 Sep 20;9:13616. doi: 10.1038/s41598-019-49897-1 (PMC6754420; doi:10.1038/s41598-019-49897-1)
Supplement: Supplementary file 1 — Supplementary Info [file 41598_2019_49897_MOESM1_ESM.pdf]

Comparative analysis of the fecal microbiota from different species of domesticated and wild suids.

Florencia Correa-Fiz[1], Miguel Blanco-Fuertes[1], Maria Jesus Navas[1], Anna Lacasta[2], Richard Bishop[2,3], Naftaly Githaka[2], Cynthia Onzere[2,3], Marie Frédérique Le Potier[4], Vanessa Almagro-Delgado[5], Jorge Martinez[1,6], Virginia Aragon[1], Fernando Rodriguez[1].

[1]IRTA, Centre de Recerca en Sanitat Animal (CReSA, IRTA-UAB), Campus de la Universitat Autònoma de Barcelona, 08193 Bellaterra, Spain.

[2]International Livestock Research Institute (ILRI), P.O. Box 30709, 00100 Nairobi, Kenya

[3]Washington State University, Department of Veterinary Microbiology and Pathology, Pullman, WA, USA

[4]ANSES, laboratory of Ploufragan-Plouzané-Niort, swine virology and immunology unit, 22440 Ploufragran, France

[5]Veterinary service Zoo Barcelona, Parc Ciudadella s/n 08003 Barcelona, Spain

[6]Departament de Sanitat i Anatomia Animals, Universitat Autònoma de Barcelona, 08193 Bellaterra, Spain

Supplementary Table S1. Number of sequences per sample obtained (input), after filtering (written), and the percentage representing the sequenced used for analysis.

Sample	total input seqs	seqs written	After filter %
AFRpig10.feces	418907	369928	88,31
AFRpig11.feces	500309	443674	88,68
AFRpig14.feces	435881	386843	88,75
AFRpig15.feces	142378	114343	80,31
AFRpig16.feces	310460	267392	86,13
AFRpig18.feces	493528	399358	80,92
AFRpig20.feces	489863	431064	88,00
AFRpig27.feces	474893	426578	89,83
AFRpig3.feces	406070	347170	85,50
AFRpig4.feces	470961	377729	80,20
AFRpig5.feces	82270	71023	86,33
AFRpig6.feces	538117	473535	88,00
AFRpig7.feces	588768	523022	88,83
AFRpig8.feces	395475	345918	87,47
AFRpig9.feces	419777	361560	86,13
AFRwarthog10.feces	268721	195885	72,90
AFRwarthog12.feces	303199	201770	66,55
AFRwarthog14.feces	292064	220736	75,58
AFRwarthog18.feces	338626	246500	72,79
AFRwarthog2.feces	217943	170973	78,45
AFRwarthog20.feces	282155	205415	72,80
AFRwarthog21.feces	417050	329852	79,09
AFRwarthog22.feces	254574	177244	69,62
AFRwarthog23.feces	208594	158619	76,04
AFRwarthog24.feces	172415	128391	74,47
AFRwarthog25.feces	176178	123559	70,13
AFRwarthog4.feces	155081	115185	74,27
AFRwarthog6.feces	143699	87849	61,13
AFRwarthog6.feces	81471	63927	78,47
AFRwarthog8.feces	36033	27348	75,90
COMpig.4feces	114703	102935	89,74
COMpig1.feces	89944	81661	90,79
COMpig2.feces	93801	85298	90,94
COMpig3.feces	102263	90913	88,90
COMpig5.feces	108482	97029	89,44
COMpig6.feces	110390	94215	85,35
COMpig7.feces	133835	115523	86,32
COMpig8.feces	49620	37087	74,74
COMpig9.feces	132296	115423	87,25
SPAwarthog1.feces	308901	273712	88,61
SPAwarthog2.feces	277310	243208	87,70
SPAwarthog3.feces	536462	477227	88,96
SPAwarthog4.feces	468062	399964	85,45
SPAwarthog5.feces	519725	463231	89,13
SPFpig1.feces	82067	75612	92,13
SPFpig10.feces	94754	85549	90,29
SPFpig11.feces	25334	21134	83,42
SPFpig2.feces	140975	128977	91,49
SPFpig3.feces	60673	51759	85,31

SPFpig4.feces	85008	78632	92,50
SPFpig5.feces	141002	132778	94,17
SPFpig6.feces	92840	85630	92,23
SPFpig7.feces	133499	123084	92,20
SPFpig8.feces	120182	108862	90,58
SPFpig9.feces	93153	83576	89,72

Supplementary Table S2. Alpha diversity and richness estimation through Shannon-Wiener and Chao indices respectively, among groups. Two-sample nonparametric t-test was used with Monte Carlo permutations to calculate the *P* values, using QIIME (vs 1.9)

Group1	Group2	Group1 mean	Group2 mean	t stat	p-value	Index
AFR warthog	SPF PIG	8.15958638316	7.00622552607	4.68015786575	0.01	shannon
AFR warthog	SPF PIG	7877.65596517	5669.45986177	4.22298680982	0.01	chao
AFR warthog	SPA warthog	7877.65596517	4307.2038146	5.92538258374	0.01	chao
AFR pig	SPA warthog	7273.3982936	4307.2038146	4.45959563909	0.01	chao
AFR pig	SPF PIG	7.77129596318	7.00622552607	3.2365382346	0.02	shannon
COM pig	SPA warthog	6755.55860122	4307.2038146	4.04425287565	0.03	chao
AFR pig	SPF PIG	7273.3982936	5669.45986177	2.92304082869	0.1	chao
COM pig	SPF PIG	7.84256613603	7.00622552607	2.7892995537	0.14	shannon
SPF PIG	SPA warthog	7.00622552607	8.05978885638	-2.9956550889	0.15	shannon
AFR pig	AFR warthog	7.77129596318	8.15958638316	-2.36019736483	0.2	shannon
COM pig	AFR warthog	6755.55860122	7877.65596517	-2.12441704608	0.46	chao
SPF PIG	SPA warthog	5669.45986177	4307.2038146	2.07312750473	0.61	chao
COM pig	SPF PIG	6755.55860122	5669.45986177	1.86757799539	0.9	chao
AFR warthog	SPA warthog	8.15958638316	8.05978885638	0.477680500336	1.0	shannon
COM pig	AFR pig	7.84256613603	7.77129596318	0.365790188425	1.0	shannon
COM pig	AFR warthog	7.84256613603	8.15958638316	-1.55339422393	1.0	shannon
COM pig	SPA warthog	7.84256613603	8.05978885638	-0.922258589567	1.0	shannon
AFR pig	SPA warthog	7.77129596318	8.05978885638	-1.45874866459	1.0	shannon
AFR pig	AFR warthog	7273.3982936	7877.65596517	-1.22181967554	1.0	chao
COM pig	AFR pig	6755.55860122	7273.3982936	-0.91988944818	1.0	chao

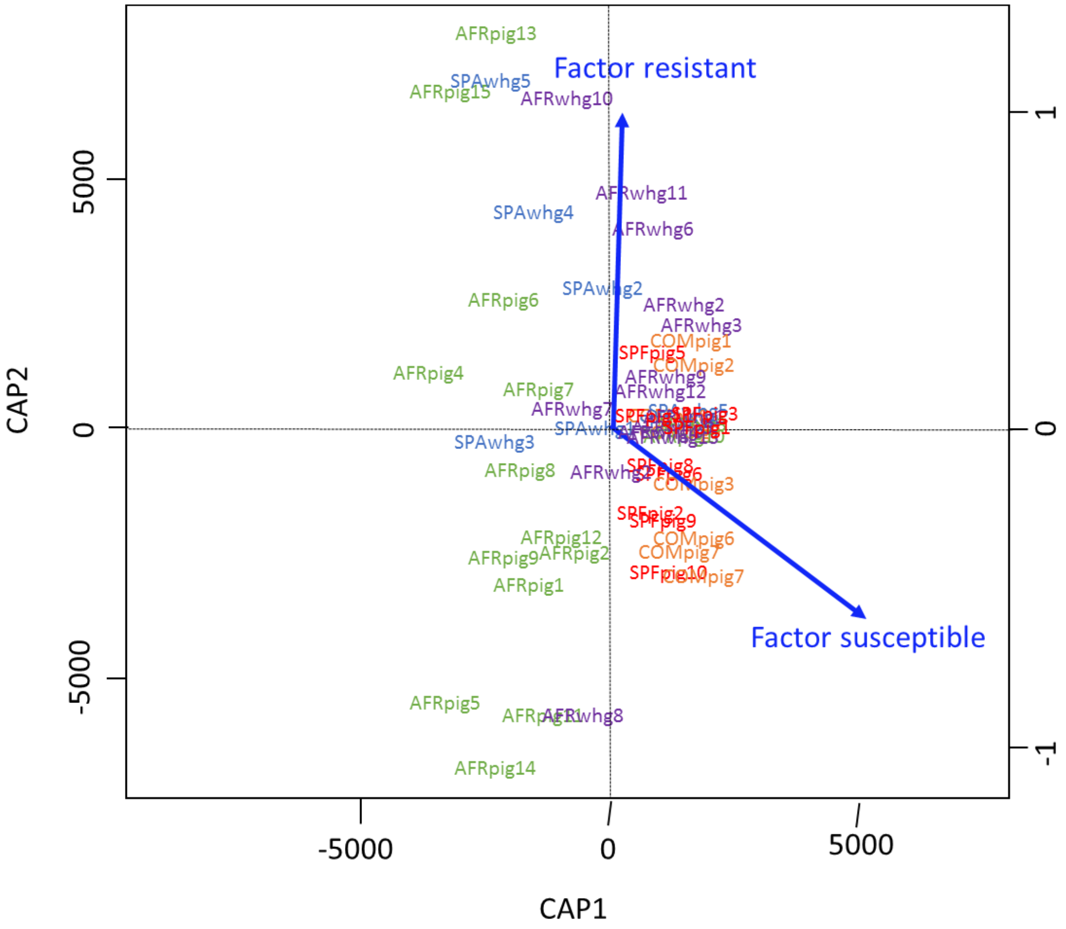

Supplementary Figure S3. Distance-based redundancy constrained analysis. The samples were divided in two groups: resistance warthogs (SPA and AFR) or susceptible pigs (SPF, COM and AFR) to explore the clustering.

Supplementary Table S4. Core OTUs found in each group analyzed (presence=1, absence=0)

OTU	COM pig CORE	SPF pig CORE	AFR warthog CORE	SPA warthog CORE
Acetanaerobacterium	1	0	1	1
Acetivibrio	1	1	0	1
Acholeplasma	0	0	0	1
Acholeplasmataceae	0	0	0	1
Acholeplasmatales	0	0	0	1
Acidaminobacter	1	0	0	1
Acidaminococcaceae	1	1	1	1
Acinetobacter	0	0	0	1
Actinobacillus	0	0	0	1
Actinobacteria	1	1	1	1
Actinomycetales	0	0	0	1
Aeromonadales	1	0	1	1
Alcaligenaceae	0	0	0	1
Alistipes	1	1	0	1
Alloprevotella	1	0	1	1
Alphaproteobacteria	0	0	0	1
Anaerofustis	0	0	1	0
Anaerolineaceae	0	0	1	0
Anaerolineae	0	0	1	0
Anaerolineales	0	0	1	0
Anaerophaga	0	0	0	1
Anaeroplasma	0	0	1	1
Anaeroplasmataceae	1	0	1	1
Anaeroplasmatales	1	0	1	1
Anaerosporobacter	0	1	0	1
Anaerostipes	0	0	0	1
Anaerotruncus	1	0	0	0
Anaerovibrio	1	0	1	1
Anaerovorax	1	0	1	1
Asteroleplasma	1	0	0	0

Bacillaceae 1	0	0	0	1
Bacillales	0	0	0	1
Bacilli	1	0	1	1
Bacillus	0	0	0	1
Bacteroidaceae	0	1	1	1
Bacteroidales	1	1	1	1
Bacteroides	0	1	1	1
Bacteroidetes	1	1	1	1
Bacteroidia	1	1	1	1
Barnesiella	1	1	1	1
Bdellovibrionaceae	1	1	0	1
Bdellovibrionales	1	1	0	1
Betaproteobacteria	1	1	1	1
Bifidobacteriaceae	0	0	0	1
Bifidobacteriales	0	0	0	1
Bifidobacterium	0	0	0	1
Bilophila	0	1	1	1
Blautia	1	1	1	1
Bulleidia	1	0	1	1
Burkholderiales	1	1	1	1
Butyricicoccus	0	1	0	1
Butyricimonas	0	1	0	0
Candidatus Saccharibacteria	0	0	0	1
Catonella	0	0	1	0
Cellulosibacter	1	0	0	0
Cellulosilyticum	0	0	1	1
Chitinophagaceae	0	0	1	0
Chloroflexi	0	0	1	0
Clostridia	1	1	1	1
Clostridiaceae 1	1	1	1	1
Clostridiales	1	1	1	1
Clostridiales_Incertae Sedis XII	1	0	0	1
Clostridiales_Incertae Sedis XIII	1	1	1	1
Clostridium III	1	0	0	0

Clostridium IV	1	1	1	1
Clostridium sensu stricto	1	0	1	1
Clostridium XI	1	1	1	1
Clostridium XlVa	1	1	1	1
Clostridium XlVb	1	1	0	1
Clostridium XVIII	0	1	1	1
Collinsella	0	0	0	1
Coprobacillus	0	1	0	1
Coprococcus	1	1	1	1
Coraliomargarita	0	0	0	1
Coriobacteriaceae	1	1	1	1
Coriobacteriales	1	1	1	1
Corynebacteriaceae	0	0	0	1
Corynebacterium	0	0	0	1
Defluviitalea	0	1	1	1
Defluviitaleaceae	0	1	1	1
Deltaproteobacteria	1	1	1	1
Desulfovibrio	1	1	0	1
Desulfovibrionaceae	1	1	1	1
Desulfovibrionales	1	1	1	1
Dialister	0	1	0	0
Dorea	1	1	1	1
Eggerthella	0	0	0	1
Elusimicrobia	0	0	0	1
Elusimicrobiaceae	0	0	0	1
Elusimicrobiales	0	0	0	1
Elusimicrobium	0	0	0	1
Enterobacteriaceae	0	1	0	1
Enterobacteriales	0	1	0	1
Enterococcaceae	0	0	0	1
Enterococcus	0	0	0	1
Erysipelothrix	0	0	1	1
Erysipelotrichaceae	1	1	1	1
Erysipelotrichaceae_incertae_sedis	0	1	0	1

Erysipelotrichales	1	1	1	1
Erysipelotrichia	1	1	1	1
Escherichia/Shigella	0	1	0	1
Ethanoligenens	1	0	0	1
Eubacteriaceae	1	1	1	1
Eubacterium	1	1	1	1
Euryarchaeota	1	0	0	1
Faecalibacterium	1	1	1	1
Fibrobacter	1	0	1	1
Fibrobacteraceae	1	0	1	1
Fibrobacterales	1	0	1	1
Fibrobacteres	1	0	1	1
Fibrobacteria	1	0	1	1
Firmicutes	1	1	1	1
Flavobacteriaceae	1	0	1	1
Flavobacteriales	1	0	1	1
Flavobacteriia	1	0	1	1
Flavonifractor	1	1	0	1
Fusibacter	0	0	0	1
Gammaproteobacteria	1	1	1	1
Gemmiger	1	0	0	1
Gordonibacter	0	1	0	1
Gracilibacteraceae	1	0	0	1
Hallella	1	0	0	1
Holdemania	1	0	1	1
Hydrogenoanaerobacterium	1	0	0	1
Insolitispirillum	0	0	0	1
Lachnoanaerobaculum	0	0	0	1
Lachnospiracea_incertae_sedis	1	1	1	1
Lachnospiraceae	1	1	1	1
Lactobacillaceae	1	0	0	1
Lactobacillales	1	0	1	1
Lactobacillus	1	0	0	1
Lentisphaerae	0	0	0	1

Lentisphaeria	0	0	0	1
Lutispora	1	0	0	1
Lysinibacillus	0	0	0	1
Marinilabiaceae	0	0	1	1
Megamonas	0	0	0	1
Methanobacteria	1	0	0	0
Methanobacteriales	1	0	0	0
Methanobrevibacter	1	0	0	0
Microbacteriaceae	0	0	0	1
Mogibacterium	0	0	0	1
Mollicutes	1	0	1	1
Moraxella	0	0	1	1
Moraxellaceae	0	0	1	1
Mycoplasma	0	0	0	1
Mycoplasmataceae	0	0	0	1
Mycoplasmatales	0	0	0	1
Negativicutes	1	1	1	1
Opitutae	0	0	0	1
Oribacterium	1	0	1	1
Ornithobacterium	0	0	0	1
Oscillibacter	1	1	1	1
Oxalobacter	0	0	1	0
Oxalobacteraceae	0	0	1	0
Paludibacter	0	0	1	1
Papillibacter	1	0	0	1
Parabacteroides	1	1	0	1
Paraprevotella	1	1	1	1
Parasutterella	0	1	0	0
Pasteurellaceae	0	0	0	1
Pasteurellales	0	0	0	1
Pelistega	0	0	0	1
Pelospora	0	0	0	1
Peptococcaceae 1	0	0	0	1
Peptococcus	0	0	0	1

Peptostreptococcaceae	1	1	1	1
Petrimonas	0	0	1	1
Phascolarctobacterium	1	1	1	1
Planctomycetaceae	1	0	1	1
Planctomycetales	1	0	1	1
Planctomycetes	1	0	1	1
Planctomycetia	1	0	1	1
Planococcaceae	0	0	0	1
Planococcaceae_incertae_sedis	0	0	0	1
Porphyromonadaceae	1	1	1	1
Prevotella	1	1	1	1
Prevotellaceae	1	1	1	1
Propionibacteriaceae	0	0	0	1
Propionibacterium	0	0	0	1
Proteobacteria	1	1	1	1
Pseudobutyrivibrio	1	1	1	1
Pseudoflavonifractor	1	0	1	1
Pseudomonadaceae	0	0	0	1
Pseudomonadales	0	0	1	1
Pseudomonas	0	0	0	1
Psychrobacter	0	0	0	1
Puniceicoccaceae	0	0	0	1
Puniceicoccales	0	0	0	1
Pyramidobacter	1	0	1	1
Rhodospirillaceae	0	0	0	1
Rhodospirillales	0	0	0	1
Rikenellaceae	1	1	0	1
Roseburia	1	1	1	1
Ruminobacter	0	0	0	1
Ruminococcaceae	1	1	1	1
Ruminococcus	1	1	1	1
Ruminococcus2	0	1	1	1
Saccharibacteria_genera_incertae_sedis	0	0	0	1
Saccharofermentans	0	0	1	0

Schwartzia	0	0	1	0
Selenomonadales	1	1	1	1
Sharpea	0	0	0	1
Solibacillus	0	0	0	1
Sphaerochaeta	1	0	1	1
Sphingobacteriaceae	1	0	1	0
Sphingobacteriales	1	0	1	0
Sphingobacteriia	1	0	1	0
Spirochaetaceae	1	0	1	1
Spirochaetales	1	0	1	1
Spirochaetes	1	0	1	1
Spirochaetia	1	0	1	1
Sporobacter	1	1	1	1
Staphylococcaceae	0	0	0	1
Staphylococcus	0	0	0	1
Streptococcaceae	0	0	1	1
Streptococcus	0	0	1	1
Subdivision5	1	0	1	1
Subdivision5_genera_incertae_sedis	1	0	1	1
Succinivibrio	1	0	1	1
Succinivibrionaceae	1	0	1	1
Sutterella	1	1	0	1
Sutterellaceae	1	1	0	1
Synergistaceae	1	0	1	1
Synergistales	1	0	1	1
Synergistetes	1	0	1	1
Synergistia	1	0	1	1
Syntrophomonadaceae	0	0	0	1
Tenericutes	1	0	1	1
Thermogymnomonas	1	0	0	1
Thermoplasmata	1	0	0	1
Thermoplasmatales	0	0	0	1
Thermoplasmatales_incertae_sedis	1	0	0	1
ThermoplasmatalesMethanobacteriaceae	1	0	0	0

Treponema	1	0	1	1
Turicibacter	1	1	0	1
Ureaplasma	0	0	0	1
Vampirovibrio	1	1	0	1
Veillonella	0	0	0	1
Veillonellaceae	1	1	1	1
Verrucomicrobia	1	0	1	1
Victivallaceae	0	0	0	1
Victivallales	0	0	0	1
Victivallis	0	0	0	1

Supplementary Table S5. Mean relative abundances of the OTUs found present in any of the five core group analysis

**Domain**	**Phyla**	**Order**	**Class**	**Family**	**Genus**	**Mean SPF pig**	**Mean COM pig**	**Mean AFR pig**	**Mean AFR warthog**	**Mean SPA warthog**
Bacteria	*Tenericutes*	*Mollicutes*	*Anaeroplasmatales*	*Anaeroplasmataceae*	*Anaeroplasma*	0,002409865	0,000946029	0,000886768	0,00193538	0,011913663
Bacteria	*Firmicutes*	*Clostridia*	*Clostridiales*	*Lachnospiraceae*	*Cellulosilyticum*	0,000155712	0,000388243	0,001875377	0,001997969	0,003928124
Bacteria	*Firmicutes*	*Erysipelotrichia*	*Erysipelotrichales*	*Erysipelotrichaceae*	*Erysipelothrix*	0	6,41854E-05	0,000939883	0,001107819	0,000130191
Bacteria	*Proteobacteria*	*Gammaproteobacteria*	*Pseudomonadales*	*Moraxellaceae*	*Moraxella*	7,04847E-07	0	1,13486E-05	0,0029372	6,06265E-05
Bacteria	*Bacteroidetes*	*Bacteroidia*	*Bacteroidales*	*Porphyromonadaceae*	*Paludibacter*	0	0,003622412	0,006583504	0,002872304	0,015553847
Bacteria	*Bacteroidetes*	*Bacteroidia*	*Bacteroidales*	*Porphyromonadaceae*	*Petrimonas*	0	0	2,47612E-05	0,000903059	0,003815899
Bacteria	*Firmicutes*	*Bacilli*	*Lactobacillales*	*Streptococcaceae*	*Streptococcus*	0,000835519	0,003901821	0,000569569	0,000339425	0,000919502
Bacteria	*Bacteroidetes*	*Bacteroidia*	*Bacteroidales*	*Marinilabiaceae*		0	0,006976556	0,003899366	0,016124349	0,000817929
Bacteria	*Firmicutes*	*Bacilli*	*Lactobacillales*	*Streptococcaceae*		0,000837265	0,0039048 9	0,000580204	0,000343252	0,000921974
Bacteria	*Proteobacteria*	*Gammaproteobacteria*	*Pseudomonadales*	*Moraxellaceae*		2,31218E-05	1,10643E-05	0,000212703	0,005778435	0,000196472
Bacteria	*Proteobacteria*	*Gammaproteobacteria*	*Pseudomonadales*			2,70035E-05	1,59926E-05	0,000218613	0,005801325	0,000225991
Bacteria	*Firmicutes*	*Bacilli*	*Lactobacillales*	*Aerococcaceae*	*Abiotrophia*	7,04847E-07	2,50577E-06	1,76493E-07	0	0
Bacteria	*Actinobacteria*	*Actinobacteria*	*Actinomycetales*	*Micrococcaceae*	*Acaricomes*	0	0	1,92724E-07	0	0
Bacteria	*Firmicutes*	*Clostridia*	*Clostridiales*	*Ruminococcaceae*	*Acetanaerobacterium*	0,026762041	0,009675083	0,00112781	0,001207151	0,004178715
Bacteria	*Firmicutes*	*Clostridia*	*Clostridiales*	*Lachnospiraceae*	*Acetitomaculum*	4,81229E-05	1,16373E-05	2,99601E-05	1,59417E-05	4,87696E-06
Bacteria	*Firmicutes*	*Clostridia*	*Clostridiales*	*Ruminococcaceae*	*Acetivibrio*	0,000990158	0,001873197	0,001677363	0,000594794	0,000769379

Bacteria	*Firmicutes*	*Clostridia*	*Clostridiales*	*Peptostreptococcaceae*	*Acetoanaerobium*	0	0	1,14011E-06	4,08149E-06	0
Bacteria	*Proteobacteria*	*Alphaproteobacteria*	*Rhodospirillales*	*Acetobacteraceae*	*Acetobacter*	0	0	9,27936E-07	0	0
Bacteria	*Firmicutes*	*Clostridia*	*Clostridiales*	*Eubacteriaceae*	*Acetobacterium*	0	0	0	4,70618E-07	0
Bacteria	*Tenericutes*	*Mollicutes*	*Acholeplasmatales*	*Acholeplasmataceae*	*Acholeplasma*	0,000522859	6,47658E-06	6,06724E-05	0,000126821	0,00804251
Bacteria	*Proteobacteria*	*Betaproteobacteria*	*Burkholderiales*	*Alcaligenaceae*	*Achromobacter*	0	0	1,5026E-07	0	0
Bacteria	*Firmicutes*	*Clostridia*	*Clostridiales*	*Clostridiales_Incertae Sedis XII*	*Acidaminobacter*	0,00012998	0,000297145	0,001394139	6,88033E-05	4,5567E-05
Bacteria	*Firmicutes*	*Negativicutes*	*Selenomonadales*	*Acidaminococcaceae*	*Acidaminococcus*	5,36781E-05	0,000103218	0,001142335	0,001169236	4,3175E-07
Bacteria	*Actinobacteria*	*Actinobacteria*	*Acidimicrobiales*	*Acidimicrobineae"_incertae_sedis*	*Aciditerrimonas*	0	0	6,02309E-07	0	0
Bacteria	*Proteobacteria*	*Alphaproteobacteria*	*Rhodospirillales*	*Acetobacteraceae*	*Acidocella*	0	0	1,40916E-06	0	1,79097E-05
Bacteria	*Actinobacteria*	*Actinobacteria*	*Actinomycetales*	*Acidothermaceae*	*Acidothermus*	0	0	1,76493E-07	0	0
Bacteria	*Proteobacteria*	*Betaproteobacteria*	*Burkholderiales*	*Comamonadaceae*	*Acidovorax*	1,99122E-06	0	1,09264E-06	4,17777E-07	1,46139E-06
Bacteria	*Proteobacteria*	*Gammaproteobacteria*	*Pseudomonadales*	*Moraxellaceae*	*Acinetobacter*	9,54688E-06	6,43171E-06	0,000175784	0,002775397	7,27491E-05
Bacteria	*Proteobacteria*	*Gammaproteobacteria*	*Pasteurellales*	*Pasteurellaceae*	*Actinobacillus*	6,18811E-05	3,00305E-06	6,14581E-05	2,39314E-05	0,000990666
Bacteria	*Actinobacteria*	*Actinobacteria*	*Actinomycetales*	*Actinomycetaceae*	*Actinobaculum*	0	1,14513E-06	0	0	0
Bacteria	*Actinobacteria*	*Actinobacteria*	*Actinomycetales*	*Actinomycetaceae*	*Actinomyces*	4,05959E-06	2,18398E-06	2,46445E-06	6,88238E-07	3,83677E-06
Bacteria	*Actinobacteria*	*Actinobacteria*	*Actinomycetales*	*Pseudonocardiaceae*	*Actinomycetospora*	0	0	5,78172E-07	0	0
Bacteria	*Actinobacteria*	*Actinobacteria*	*Actinomycetales*	*Micromonosporaceae*	*Actinoplanes*	0	0	3,6088E-07	0	0
Bacteria	*Bacteroidetes*	*Cytophagia*	*Cytophagales*	*Cytophagaceae*	*Adhaeribacter*	1,15613E-06	0	8,82467E-07	0	0
Bacteria	*Actinobacteria*	*Actinobacteria*	*Coriobacteriales*	*Coriobacteriaceae*	*Adlercreutzia*	4,00055E-05	0	1,17475E-05	0,000134094	0
Bacteria	*Proteobacteria*	*Betaproteobacteria*	*Burkholderiales*	*Alcaligenaceae*	*Advenella*	0	0	3,5255E-07	3,29433E-06	1,50014E-06

Bacteria	*Firmicutes*	*Bacilli*	*Bacillales*	*Bacillaceae 1*	*Aeribacillus*	0	0	3,52987 E-07	0	0
Bacteria	*Firmicutes*	*Bacilli*	*Lactobacillales*	*Aerococcaceae*	*Aerococcus*	1,08774 E-06	0	5,53125 E-05	3,22492 E-05	1,36432E -05
Bacteria	*Actinobacteria*	*Actinobacteria*	*Actinomycetales*	*Nocardioidaceae*	*Aeromicrobium*	0	0	1,80215 E-07	0	0
Bacteria	*Proteobacteria*	*Gammaproteobacteria*	*Aeromonadales*	*Aeromonadaceae*	*Aeromonas*	7,38594 E-07	0	1,80215 E-07	4,18272 E-06	0
Bacteria	*Proteobacteria*	*Gammaproteobacteria*	*Pasteurellales*	*Pasteurellaceae*	*Aggregatibacter*	0	0	3,43604 E-06	0	8,22341E -07
Bacteria	*Actinobacteria*	*Actinobacteria*	*Actinomycetales*	*Microbacteriaceae*	*Agrococcus*	0	0	1,84386 E-07	0	0
Bacteria	*Actinobacteria*	*Actinobacteria*	*Actinomycetales*	*Microbacteriaceae*	*Agromyces*	0	0	1,54656 E-07	0	0
Bacteria	*Verrucomicrobi a*	*Verrucomicrobiae*	*Verrucomicrobiales*	*Verrucomicrobiaceae*	*Akkermansia*	0,00167 0242	0,0024480 03	0,01036 513	6,95456 E-07	9,9488E-06
Bacteria	*Proteobacteria*	*Gammaproteobacteria*	*Alteromonadales*	*Alteromonadaceae*	*Alishewanella*	0	0	1,80215 E-07	6,2012E -07	0
Bacteria	*Bacteroidetes*	*Bacteroidia*	*Bacteroidales*	*Rikenellaceae*	*Alistipes*	0,02740 3396	0,0005007 72	0,00016 3167	6,29729 E-06	0,000880 643
Bacteria	*Firmicutes*	*Clostridia*	*Clostridiales*	*Eubacteriaceae*	*Alkalibacter*	0	1,17934E-06	5,53159 E-07	0	4,3175E-07
Bacteria	*Firmicutes*	*Bacilli*	*Lactobacillales*	*Carnobacteriaceae*	*Alkalibacterium*	0	0	1,51678 E-06	0	1,46139E -06
Bacteria	*Firmicutes*	*Clostridia*	*Clostridiales*	*Eubacteriaceae*	*Alkalibaculum*	0	0	1,80215 E-07	0	0
Bacteria	*Bacteroidetes*	*Bacteroidia*	*Bacteroidales*	*Marinilabiaceae*	*Alkaliflexus*	0	0	3,56721 E-07	3,47728 E-07	0
Bacteria	*Bacteroidetes*	*Bacteroidia*	*Bacteroidales*	*Marinilabiliaceae*	*Alkalitalea*	0	0	1,16074 E-06	2,16547 E-07	8,50838E -07
Bacteria	*Proteobacteria*	*Gammaproteobacteria*	*Pseudomonadales*	*Moraxellaceae*	*Alkanindiges*	8,35086 E-07	0	1,58428 E-06	1,51583 E-06	0
Bacteria	*Firmicutes*	*Negativicutes*	*Selenomonadales*	*Veillonellaceae*	*Allisonella*	0	6,43443E-05	6,73241 E-05	5,48676 E-05	4,3175E-07
Bacteria	*Firmicutes*	*Erysipelotrichia*	*Erysipelotrichales*	*Erysipelotrichaceae*	*Allobaculum*	1,15613 E-06	0	8,84862 E-07	1,80156 E-06	0
Bacteria	*Firmicutes*	*Bacilli*	*Lactobacillales*	*Carnobacteriaceae*	*Alloiococcus*	1,06165 E-06	9,62643E-07	9,38663 E-07	0	5,67671E -06
Bacteria	*Bacteroidetes*	*Bacteroidia*	*Bacteroidales*	*Prevotellaceae*	*Alloprevotella*	1,42144 E-05	0,0672594 47	0,01488 8745	0,00820 818	0,004091 312

Bacteria	*Proteobacteria*	*Alphaproteobacteria*	*Sphingomonadales*	*Erythrobacteraceae*	*Altererythrobacter*	1,15613E-06	0	6,70979E-07	0	0
Bacteria	*Proteobacteria*	*Betaproteobacteria*	*Neisseriales*	*Neisseriaceae*	*Alysiella*	0	0	4,89058E-07	0	1,43184E-06
Bacteria	*Proteobacteria*	*Alphaproteobacteria*	*Rhodobacterales*	*Rhodobacteraceae*	*Amaricoccus*	0	0	8,47489E-07	0	0
Bacteria	*Firmicutes*	*Bacilli*	*Bacillales*	*Bacillaceae 2*	*Amphibacillus*	0	0	0	0	8,635E-07
Bacteria	*Firmicutes*	*Negativicutes*	*Selenomonadales*	*Veillonellaceae*	*Anaeroarcus*	0	0	0	7,0802E-07	0
Bacteria	*Firmicutes*	*Clostridia*	*Clostridiales*	*Clostridiaceae 1*	*Anaerobacter*	4,41335E-06	1,19633E-05	9,21187E-05	6,50071E-05	3,43366E-06
Bacteria	*Proteobacteria*	*Gammaproteobacteria*	*Aeromonadales*	*Succinivibrionaceae*	*Anaerobiospirillum*	0	3,00472E-06	0	0,000680819	0
Bacteria	*Firmicutes*	*Clostridia*	*Clostridiales*	*Clostridiales_Incertae Sedis XI*	*Anaerococcus*	7,11841E-05	4,40557E-05	4,7755E-06	1,39626E-05	1,10136E-05
Bacteria	*Firmicutes*	*Clostridia*	*Clostridiales*	*Ruminococcaceae*	*Anaerofilum*	1,8958E-05	3,31082E-05	0,001328499	3,74322E-06	8,22341E-07
Bacteria	*Firmicutes*	*Clostridia*	*Clostridiales*	*Eubacteriaceae*	*Anaerofustis*	9,19292E-06	8,52203E-05	2,26311E-05	0,001480234	8,80084E-06
Bacteria	*Firmicutes*	*Negativicutes*	*Selenomonadales*	*Veillonellaceae*	*Anaeroglobus*	1,08774E-06	0	1,78939E-06	0	0
Bacteria	*Bacteroidetes*	*Bacteroidia*	*Bacteroidales*	*Marinilabiaceae*	*Anaerophaga*	0	0,006918874	0,003877434	0,003568019	0,000815997
Bacteria	*Bacteroidetes*	*Bacteroidia*	*Bacteroidales*	*Bacteroidaceae*	*Anaerorhabdus*	7,04847E-07	3,58084E-06	0,00028311	0,000665639	5,00045E-07
Bacteria	*Firmicutes*	*Clostridia*	*Clostridiales*	*Incertae Sedis XI*	*Anaerosphaera*	0	0	0	1,86036E-06	0
Bacteria	*Firmicutes*	*Clostridia*	*Clostridiales*	*Clostridiaceae 1*	*Anaerosporobacter*	0,000773948	0,000521201	0,000236617	0,000524648	0,000169202
Bacteria	*Firmicutes*	*Clostridia*	*Clostridiales*	*Lachnospiraceae*	*Anaerostipes*	0,000528183	0,000270193	5,78693E-05	0,000240736	0,007491562
Bacteria	*Firmicutes*	*Clostridia*	*Clostridiales*	*Ruminococcaceae*	*Anaerotruncus*	0,000814477	0,0007186 8	0,003005857	0,000127451	0,002135014
Bacteria	*Firmicutes*	*Negativicutes*	*Selenomonadales*	*Veillonellaceae*	*Anaerovibrio*	0	0,004330536	0,003136723	0,001769561	0,0023892
Bacteria	*Firmicutes*	*Clostridia*	*Clostridiales*	*Clostridiales_Incertae Sedis XIII*	*Anaerovorax*	0,000678883	0,000543155	0,001098271	0,005374011	0,000562862
Bacteria	*Firmicutes*	*Bacilli*	*Bacillales*	*Bacillaceae 1*	*Anoxybacillus*	0	0	5,88364E-07	0	0

Bacteria	*Firmicutes*	*Clostridia*	*Clostridiales*	*Clostridiaceae 2*	*Anoxynatronum*	0	1,14513E-06	3,32776E-07	1,0379E-06	0
Bacteria	*Proteobacteria*	*Betaproteobacteria*	*Burkholderiales*	*Burkholderiales_incertae_sedis*	*Aquabacterium*	0	0	0	1,88247E-06	8,22341E-06
Bacteria	*Proteobacteria*	*Alphaproteobacteria*	*Rhizobiales*	*Phyllobacteriaceae*	*Aquamicrobium*	0	0	3,0768E-07	0	2,19209E-06
Bacteria	*Proteobacteria*	*Gammaproteobacteria*	*Legionellales*	*Coxiellaceae*	*Aquicella*	0	0	4,98644E-07	0	1,25726E-06
Bacteria	*Planctomycetes*	*Planctomycetia*	*Planctomycetales*	*Planctomycetaceae*	*Aquisphaera*	0	0	1,53397E-06	2,89771E-07	0
Bacteria	*Actinobacteria*	*Actinobacteria*	*Actinomycetales*	*Actinomycetaceae*	*Arcanobacterium*	0	0	3,18177E-05	1,69937E-05	0
Bacteria	*Proteobacteria*	*Deltaproteobacteria*	*Myxococcales*	*Cystobacteraceae*	*Archangium*	0	0	3,52987E-07	0	0
Bacteria	*Proteobacteria*	*Epsilonproteobacteria*	*Campylobacterales*	*Campylobacteraceae*	*Arcobacter*	0	0	1,92724E-07	2,78394E-05	0
Bacteria	*Bacteroidetes*	*Sphingobacteriia*	*Sphingobacteriales*	*Sphingobacteriaceae*	*Arcticibacter*	0	0	0	4,23557E-06	0
Bacteria	*Proteobacteria*	*Gammaproteobacteria*	*Xanthomonadales*	*Xanthomonadaceae*	*Arenimonas*	0	0	1,56282E-07	0	0
Bacteria	*Armatimonadetes*	*Armatimonadetes_gp4*	*Armatimonadetes_gp4*	*Armatimonadetes_gp4*	*Armatimonadetes_gp4*	0	0	3,85448E-07	0	0
Bacteria	*Armatimonadetes*	*Armatimonadetes_gp5*	*Armatimonadetes_gp5*	*Armatimonadetes_gp5*	*Armatimonadetes_gp5*	0	0	4,56803E-07	2,16547E-07	0
Bacteria	*Armatimonadetes*	*Armatimonadia*	*Armatimonadales*	*Armatimonadaceae*	*Armatimonas/Armatimonadetes_gp1*	0	0	5,2948E-07	0	0
Bacteria	*Proteobacteria*	*Gammaproteobacteria*	*Enterobacteriales*	*Enterobacteriaceae*	*Arsenophonus*	0	0	1,56282E-07	0	0
Bacteria	*Actinobacteria*	*Actinobacteria*	*Actinomycetales*	*Micrococcaceae*	*Arthrobacter*	0	0	1,30032E-05	0,004071585	7,00995E-06
Bacteria	*Actinobacteria*	*Actinobacteria*	*Coriobacteriales*	*Coriobacteriaceae*	*Asaccharobacter*	0	0	0	3,5401E-07	0
Bacteria	*Tenericutes*	*Mollicutes*	*Anaeroplasmatales*	*Anaeroplasmataceae*	*Asteroleplasma*	1,36934E-06	0,000248052	2,74518E-05	0,000305253	1,67635E-06
Bacteria	*Actinobacteria*	*Actinobacteria*	*Coriobacteriales*	*Coriobacteriaceae*	*Atopobium*	0	1,22217E-06	8,56234E-07	3,47728E-07	0
Bacteria	*Firmicutes*	*Bacilli*	*Lactobacillales*	*Carnobacteriaceae*	*Atopostipes*	0	0	1,80345E-06	9,35203E-07	1,33843E-05

Bacteria	*Proteobacteria*	*Alphaproteobacteria*	*Rhizobiales*	*Aurantimonadaceae*	*Aurantimonas*	0	0	4,52376 E-06	5,78093 E-07	0
Bacteria	*Proteobacteria*	*Alphaproteobacteria*	*Rhizobiales*	*Aurantimonadaceae*	*Aureimonas*	0	0	3,22049 E-06	2,89771 E-07	0
Bacteria	*Proteobacteria*	*Betaproteobacteria*	*Burkholderiales*	*Alcaligenaceae*	*Azohydromonas*	0	0	1,72335 E-07	0	0
Bacteria	*Proteobacteria*	*Gammaproteobacteria*	*Pseudomonadales*	*Pseudomonadaceae*	*Azorhizophilus*	0	0	1,72335 E-07	0	0
Bacteria	*Proteobacteria*	*Alphaproteobacteria*	*Rhodospirillales*	*Rhodospirillaceae*	*Azospirillum*	0	0	1,54656 E-07	0	0
Bacteria	*Firmicutes*	*Bacilli*	*Bacillales*	*Bacillaceae 1*	*Bacillus*	0	0	0,01413 9802	0,00247 612	0,003627 793
Bacteria	*Proteobacteria*	*Deltaproteobacteria*	*Bdellovibrionales*	*Bacteriovoracaceae*	*Bacteriovorax*	0	0	0	0	4,11171E -06
Bacteria	*Bacteroidetes*	*Bacteroidia*	*Bacteroidales*	*Bacteroidaceae*	*Bacteroides*	0,05184 3576	0,0120335 97	0,01998 306	0,00130 5522	0,028344 895
Bacteria	*Proteobacteria*	*Alphaproteobacteria*	*Rhizobiales*	*Bradyrhizobiaceae*	*Balneimonas*	0	0	3,21591 E-07	0	0
Bacteria	*Bacteroidetes*	*Bacteroidia*	*Bacteroidales*	*Porphyromonadaceae*	*Barnesiella*	0,00792 95	0,0261486 33	0,05301 9252	0,01285 846	0,011829 223
Bacteria	*Firmicutes*	*Bacilli*	*Lactobacillales*	*Enterococcaceae*	*Bavariicoccus*	0	0	1,92724 E-07	0	0
Bacteria	*Proteobacteria*	*Deltaproteobacteria*	*Bdellovibrionales*	*Bdellovibrionaceae*	*Bdellovibrio*	1,06266 E-06	0	1,06298 E-06	5,78093 E-07	0
Bacteria	*Proteobacteria*	*Alphaproteobacteria*	*Rhizobiales*	*Beijerinckiaceae*	*Beijerinckia*	0	0	2,13319 E-06	4,02996 E-07	4,11171E -06
Bacteria	*Chloroflexi*	*Anaerolineae*	*Anaerolineales*	*Anaerolineaceae*	*Bellilinea*	0	0	0	8,14961 E-07	0
Bacteria	*Proteobacteria*	*Alphaproteobacteria*	*Rhodospirillales*	*Acetobacteraceae*	*Belnapia*	0	0	3,31149 E-07	0	0
Bacteria	*Proteobacteria*	*Betaproteobacteria*	*Neisseriales*	*Neisseriaceae*	*Bergeriella*	0	0	0	0	1,46139E -06
Bacteria	*Bacteroidetes*	*Flavobacteriia*	*Flavobacteriales*	*Flavobacteriaceae*	*Bergeyella*	0	0	0	5,04636 E-05	0
Bacteria	*Firmicutes*	*Bacilli*	*Bacillales*	*Planococcaceae*	*Bhargavaea*	0	0	5,30892 E-07	0	0
Bacteria	*Proteobacteria*	*Gammaproteobacteria*	*Pasteurellales*	*Pasteurellaceae*	*Bibersteinia*	0	0	3,52987 E-07	0	0
Bacteria	*Actinobacteria*	*Actinobacteria*	*Bifidobacteriales*	*Bifidobacteriaceae*	*Bifidobacterium*	0	2,201E-05	0,00134 7169	0	0,000223 29

Bacteria	*Proteobacteria*	*Deltaproteobacteria*	*Desulfovibrionales*	*Desulfovibrionaceae*	*Bilophila*	0,000322018	0,000198777	0,00019513	0,00040411	8,09122E-05
Bacteria	*Acidobacteria*	*Acidobacteria_Gp4*	*Blastocatella*	*Blastocatella*	*Blastocatella*	0	0	8,82467E-07	6,49642E-07	0
Bacteria	*Actinobacteria*	*Actinobacteria*	*Actinomycetales*	*Geodermatophilaceae*	*Blastococcus*	0	0	6,66021E-07	1,24024E-06	0
Bacteria	*Planctomycetes*	*Planctomycetia*	*Planctomycetales*	*Planctomycetaceae*	*Blastopirellula*	1,15613E-06	5,16562E-06	1,7428E-05	2,16547E-07	0
Bacteria	*Firmicutes*	*Clostridia*	*Clostridiales*	*Lachnospiraceae*	*Blautia*	0,004081855	0,001412472	0,000973308	0,000960842	0,001524311
Bacteria	*Proteobacteria*	*Betaproteobacteria*	*Burkholderiales*	*Alcaligenaceae*	*Bordetella*	0	0	6,01042E-07	0,000171604	0
Bacteria	*Proteobacteria*	*Alphaproteobacteria*	*Rhizobiales*	*Bradyrhizobiaceae*	*Bosea*	1,06165E-06	1,36064E-06	1,12856E-06	2,16547E-07	5,00045E-07
Bacteria	*Actinobacteria*	*Actinobacteria*	*Actinomycetales*	*Dermabacteraceae*	*Brachybacterium*	0	0	1,57647E-05	2,16547E-07	2,67644E-06
Bacteria	*Proteobacteria*	*Betaproteobacteria*	*Burkholderiales*	*Comamonadaceae*	*Brachymonas*	0	0	0	5,78093E-07	0
Bacteria	*Proteobacteria*	*Alphaproteobacteria*	*Rhizobiales*	*Bradyrhizobiaceae*	*Bradyrhizobium*	0	0	2,10514E-06	1,44606E-05	1,14072E-05
Bacteria	*BRC1*	*BRC1_genera_incertae_sedis*	*BRC1_genera_incertae_sedis*	*BRC1_genera_incertae_sedis*	*BRC1_genera_incertae_sedis*	0	0	0	2,16547E-07	0
Bacteria	*Firmicutes*	*Bacilli*	*Bacillales*	*Paenibacillaceae 1*	*Brevibacillus*	0	0	1,76493E-07	0	0
Bacteria	*Actinobacteria*	*Actinobacteria*	*Actinomycetales*	*Brevibacteriaceae*	*Brevibacterium*	3,42656E-06	0	1,96173E-06	6,2012E-07	5,98119E-06
Bacteria	*Proteobacteria*	*Alphaproteobacteria*	*Caulobacterales*	*Caulobacteraceae*	*Brevundimonas*	2,56583E-06	0	1,5541E-06	1,00365E-06	2,37538E-06
Bacteria	*Firmicutes*	*Bacilli*	*Bacillales*	*Listeriaceae*	*Brochothrix*	0	0	0	0	2,15875E-06
Bacteria	*Actinobacteria*	*Actinobacteria*	*Actinomycetales*	*Propionibacteriaceae*	*Brooklawnia*	0	0	9,6362E-07	0	0
Bacteria	*Firmicutes*	*Erysipelotrichia*	*Erysipelotrichales*	*Erysipelotrichaceae*	*Bulleidia*	3,12573E-06	0,002045783	0,000678475	0,000836796	0,000102553
Bacteria	*Proteobacteria*	*Betaproteobacteria*	*Burkholderiales*	*Burkholderiaceae*	*Burkholderia*	0	0	1,18051E-06	2,89771E-07	1,50014E-06
Bacteria	*Firmicutes*	*Clostridia*	*Clostridiales*	*Ruminococcaceae*	*Butyricicoccus*	0,005568386	0,000920445	0,000899393	0,000884279	0,003761197
Bacteria	*Bacteroidetes*	*Bacteroidia*	*Bacteroidales*	*Porphyromonadaceae*	*Butyricimonas*	0,002741497	4,66523E-05	9,51575E-05	5,46908E-05	0,000166455

Bacteria	*Firmicutes*	*Clostridia*	*Clostridiales*	*Lachnospiraceae*	*Butyrivibrio*	0	8,18694E-06	0,00030855	3,57345E-06	4,38417E-06
Bacteria	*Proteobacteria*	*Deltaproteobacteria*	*Myxococcales*	*Polyangiaceae*	*Byssovorax*	0	0	5,17006E-07	0	0
Bacteria	*Proteobacteria*	*Betaproteobacteria*	*Burkholderiales*	*Comamonadaceae*	*Caenimonas*	0	0	0	0	7,09936E-06
Bacteria	*Chloroflexi*	*Caldilineae*	*Caldilineales*	*Caldilineaceae*	*Caldilinea*	1,15613E-06	0	0	0	0
Bacteria	*Proteobacteria*	*Betaproteobacteria*	*Burkholderiales*	*Comamonadaceae*	*Caldimonas*	0	0	3,12565E-07	0	0
Bacteria	*Proteobacteria*	*Epsilonproteobacteria*	*Campylobacterales*	*Campylobacteraceae*	*Campylobacter*	1,41169E-05	0,003820261	0,0005449	0,008093497	5,87048E-05
Bacteria	*Proteobacteria*	*Gammaproteobacteria*	*Candidatus Carsonella*	*Candidatus Carsonella*	*Candidatus Carsonella*	9,43072E-06	2,60525E-06	1,48739E-06	0	0
Bacteria	*Acidobacteria*	*Acidobacteria_Gp1*	*Candidatus Koribacter*	*Candidatus Koribacter*	*Candidatus Koribacter*	0	0	1,92029E-07	6,2012E-07	0
Bacteria	*Bacteroidetes*	*Flavobacteriia*	*Flavobacteriales*	*Flavobacteriaceae*	*Capnocytophaga*	1,67017E-06	0	0	0	2,00018E-06
Bacteria	*Proteobacteria*	*Gammaproteobacteria*	*Cardiobacteriales*	*Cardiobacteriaceae*	*Cardiobacterium*	0	0	1,56282E-07	0	0
Bacteria	*Firmicutes*	*Bacilli*	*Lactobacillales*	*Carnobacteriaceae*	*Carnobacterium*	0	0	1,27464E-07	0	3,454E-06
Bacteria	*Firmicutes*	*Bacilli*	*Bacillales*	*Planococcaceae*	*Caryophanon*	0	1,22217E-06	6,7923E-05	0,000523828	2,42821E-05
Bacteria	*Proteobacteria*	*Alphaproteobacteria*	*Rhodobacterales*	*Rhodobacteraceae*	*Catellibacterium*	0	0	1,92029E-07	0	0
Bacteria	*Firmicutes*	*Erysipelotrichia*	*Erysipelotrichales*	*Erysipelotrichaceae*	*Catenibacterium*	0	2,23061E-05	3,68772E-07	0,0008913	0
Bacteria	*Firmicutes*	*Clostridia*	*Clostridiales*	*Lachnospiraceae*	*Catonella*	8,41608E-06	3,3697E-06	4,8974E-06	0,004276762	0
Bacteria	*Proteobacteria*	*Alphaproteobacteria*	*Caulobacterales*	*Caulobacteraceae*	*Caulobacter*	0	0	0	8,8341E-07	0
Bacteria	*Actinobacteria*	*Actinobacteria*	*Actinomycetales*	*Cellulomonadaceae*	*Cellulomonas*	0	0	1,10121E-06	1,01429E-06	0
Bacteria	*Firmicutes*	*Clostridia*	*Clostridiales*	*Ruminococcaceae*	*Cellulosibacter*	0,000161548	0,000584094	0,00024638	1,5856E-05	3,11484E-05
Bacteria	*Actinobacteria*	*Actinobacteria*	*Actinomycetales*	*Promicromonosporaceae*	*Cellulosimicrobium*	0	0	1,13133E-06	2,16547E-07	5,0482E-06
Bacteria	*Firmicutes*	*Negativicutes*	*Selenomonadales*	*Veillonellaceae*	*Centipeda*	0	0	4,50781E-07	0	0

Bacteria	*Verrucomicrobia*	*Opitutae*	*Puniceicoccales*	*Puniceicoccaceae*	*Cerasicoccus*	0	2,52479E-06	7,08043E-07	1,1258E-06	1,68584E-06
Bacteria	*Bacteroidetes*	*Cytophagia*	*Cytophagales*	*Flammeovirgaceae*	*Cesiribacter*	0	0	1,27464E-07	0	0
Bacteria	*Fusobacteria*	*Fusobacteriia*	*Fusobacteriales*	*Fusobacteriaceae*	*Cetobacterium*	0	0	3,52402E-05	0	4,06231E-05
Bacteria	*Proteobacteria*	*Alphaproteobacteria*	*Rhizobiales*	*Beijerinckiaceae*	*Chelatococcus*	0	0	8,61676E-07	3,23593E-07	0
Bacteria	*Proteobacteria*	*Gammaproteobacteria*	*Pasteurellales*	*Pasteurellaceae*	*Chelonobacter*	0	0	4,98644E-07	0	0
Bacteria	*Chlamydiae*	*Chlamydiia*	*Chlamydiales*	*Chlamydiaceae*	*Chlamydia*	0	0,00069837	7,64786E-07	0,000768759	0
Bacteria	*Chlamydiae*	*Chlamydiia*	*Chlamydiales*	*Chlamydiaceae*	*Chlamydophila*	0	0	0	2,16547E-07	0
Bacteria	*Cyanobacteria/ Chloroplast*	*Chloroplast*	*Chloroplast*	*Chloroplast*	*Chlorophyta*	2,17548E-06	1,22217E-06	0	4,50315E-07	7,30695E-07
Bacteria	*Bacteroidetes*	*Flavobacteriia*	*Flavobacteriales*	*Flavobacteriaceae*	*Chryseobacterium*	4,60748E-06	0	1,49143E-05	4,61763E-06	6,31162E-06
Bacteria	*Firmicutes*	*Bacilli*	*Bacillales*	*Planococcaceae*	*Chryseomicrobium*	1,06266E-06	0	5,11598E-06	6,49642E-07	0
Bacteria	*Armatimonadetes*	*Chthonomonadetes*	*Chthonomonadales*	*Chthonomonadaceae*	*Chthonomonas/Armatimonadetes_gp3*	0	0	2,8157E-07	0	0
Bacteria	*Proteobacteria*	*Alphaproteobacteria*	*Rhodobacterales*	*Rhodobacteraceae*	*Citreicella*	0	0	5,76087E-07	0	0
Bacteria	*Actinobacteria*	*Actinobacteria*	*Actinomycetales*	*Micrococcaceae*	*Citricoccus*	0	0	3,52987E-07	0	0
Bacteria	*Proteobacteria*	*Gammaproteobacteria*	*Enterobacteriales*	*Enterobacteriaceae*	*Citrobacter*	5,22367E-06	2,44434E-06	0,00011959	4,8808E-06	1,15945E-05
Bacteria	*Synergistetes*	*Synergistia*	*Synergistales*	*Synergistaceae*	*Cloacibacillus*	0	0,000206507	1,53944E-05	7,71787E-07	0
Bacteria	*Bacteroidetes*	*Flavobacteriia*	*Flavobacteriales*	*Flavobacteriaceae*	*Cloacibacterium*	7,38594E-07	0	0	1,03831E-05	1,64468E-06
Bacteria	*Firmicutes*	*Clostridia*	*Clostridiales*	*Clostridiaceae 3*	*Clostridiisalibacter*	0	0	1,54656E-07	0	0
Bacteria	*Firmicutes*	*Clostridia*	*Clostridiales*	*Ruminococcaceae*	*Clostridium III*	0,000629549	0,00058693	0,00059249	4,98188E-06	4,59565E-05
Bacteria	*Firmicutes*	*Clostridia*	*Clostridiales*	*Ruminococcaceae*	*Clostridium IV*	0,022132887	0,016273929	0,020885222	0,002763672	0,020615903

Bacteria	*Firmicutes*	*Clostridia*	*Clostridiales*	*Clostridiaceae 1*	*Clostridium sensu stricto*	0,000654546	0,001292071	0,004872062	0,003179614	0,001380002
Bacteria	*Firmicutes*	*Clostridia*	*Clostridiales*	*Peptostreptococcaceae*	*Clostridium XI*	0,003283809	0,00247612	0,002555779	0,005659529	0,018140686
Bacteria	*Fusobacteria*	*Fusobacteriia*	*Fusobacteriales*	*Fusobacteriaceae*	*Clostridium XIX*	0	0	1,80399E-05	2,07796E-06	0
Bacteria	*Firmicutes*	*Clostridia*	*Clostridiales*	*Lachnospiraceae*	*Clostridium XlVa*	0,037975199	0,048915641	0,078022258	0,070091924	0,064187634
Bacteria	*Firmicutes*	*Clostridia*	*Clostridiales*	*Lachnospiraceae*	*Clostridium XlVb*	0,002114271	0,000863059	0,002317586	0,000288226	0,001015246
Bacteria	*Firmicutes*	*Erysipelotrichia*	*Erysipelotrichales*	*Erysipelotrichaceae*	*Clostridium XVIII*	0,003130837	0,000121553	0,00039206	0,00146352	0,000893301
Bacteria	*Actinobacteria*	*Actinobacteria*	*Coriobacteriales*	*Coriobacteriaceae*	*Collinsella*	3,65015E-05	1,66297E-05	4,84107E-05	0,000125809	6,98399E-05
Bacteria	*Proteobacteria*	*Betaproteobacteria*	*Burkholderiales*	*Comamonadaceae*	*Comamonas*	0	0	1,06372E-05	4,70618E-07	3,80978E-06
Bacteria	*Actinobacteria*	*Actinobacteria*	*Solirubrobacterales*	*Conexibacteraceae*	*Conexibacter*	1,15613E-06	0	8,44179E-07	0	0
Bacteria	*Firmicutes*	*Erysipelotrichia*	*Erysipelotrichales*	*Erysipelotrichaceae*	*Coprobacillus*	0,001452342	0,000233167	0,000232017	0,00012997	0,001189359
Bacteria	*Firmicutes*	*Clostridia*	*Clostridiales*	*Lachnospiraceae*	*Coprococcus*	0,008928015	0,004204126	0,001588002	0,002454789	0,001001681
Bacteria	*Verrucomicrobia*	*Opitutae*	*Puniceicoccales*	*Puniceicoccaceae*	*Coraliomargarita*	6,67592E-05	0,000307531	3,88577E-05	1,70017E-05	0,000248547
Bacteria	*Proteobacteria*	*Deltaproteobacteria*	*Myxococcales*	*Myxococcaceae*	*Corallococcus*	0	0	0	0	1,00009E-06
Bacteria	*Actinobacteria*	*Actinobacteria*	*Coriobacteriales*	*Coriobacteriaceae*	*Coriobacterium*	6,81966E-06	0	1,54656E-07	0	0
Bacteria	*Actinobacteria*	*Actinobacteria*	*Actinomycetales*	*Corynebacteriaceae*	*Corynebacterium*	1,86243E-05	2,5392E-05	6,46589E-05	8,69254E-06	0,000183527
Bacteria	*Proteobacteria*	*Gammaproteobacteria*	*Enterobacteriales*	*Enterobacteriaceae*	*Cosenzaea*	0	0	0	3,5401E-07	0
Bacteria	*Proteobacteria*	*Gammaproteobacteria*	*Legionellales*	*Coxiellaceae*	*Coxiella*	0	0	7,68116E-07	0,000110221	0
Bacteria	*Proteobacteria*	*Alphaproteobacteria*	*Rhodospirillales*	*Acetobacteraceae*	*Craurococcus*	0	0	3,31149E-07	0	0
Bacteria	*Proteobacteria*	*Gammaproteobacteria*	*Enterobacteriales*	*Enterobacteriaceae*	*Cronobacter*	0	0	4,40386E-05	0	1,82434E-05
Bacteria	*Bacteroidetes*	*Flavobacteriia*	*Flavobacteriales*	*Flavobacteriaceae*	*Cruoricaptor*	0	1,08505E-05	1,19665E-05	1,03755E-05	4,19088E-07

Bacteria	*Proteobacteria*	*Betaproteobacteria*	*Burkholderiales*	*Burkholderiaceae*	*Cupriavidus*	0	0	1,54656 E-07	0	0
Bacteria	*Actinobacteria*	*Actinobacteria*	*Actinomycetales*	*Microbacteriaceae*	*Curtobacterium*	0	0	7,47966 E-07	0	0
Bacteria	*Proteobacteria*	*Betaproteobacteria*	*Burkholderiales*	*Comamonadaceae*	*Curvibacter*	0	0	9,38663 E-07	0	7,46325E -06
Bacteria	*Proteobacteria*	*Deltaproteobacteria*	*Myxococcales*	*Cystobacteraceae*	*Cystobacter*	0	0	1,76493 E-07	0	0
Bacteria	*Actinobacteria*	*Actinobacteria*	*Actinomycetales*	*Micromonosporaceae*	*Dactylosporangi um*	0	0	3,12565 E-07	0	0
Bacteria	*Proteobacteria*	*Betaproteobacteria*	*Rhodocyclales*	*Rhodocyclaceae*	*Dechloromonas*	0	0	1,56282 E-07	0	0
Bacteria	*Firmicutes*	*Clostridia*	*Clostridiales*	*Defluviitaleaceae*	*Defluviitalea*	0,00075 7012	0,0001402 38	0,00013 9525	0,00091 8847	0,000154 871
Bacteria	*Firmicutes*	*Clostridia*	*Clostridiales*	*Peptococcaceae 1*	*Dehalobacter*	0	0	1,40785 E-07	0	0
Bacteria	*Deinococcus-Thermus*	*Deinococci*	*Deinococcales*	*Deinococcaceae*	*Deinococcus*	3,4684E -06	0	1,80215 E-07	4,70618 E-07	8,635E-07
Bacteria	*Proteobacteria*	*Betaproteobacteria*	*Burkholderiales*	*Comamonadaceae*	*Delftia*	1,06266 E-06	1,07943E-06	0	3,95755 E-06	8,50671E -06
Bacteria	*Actinobacteria*	*Actinobacteria*	*Actinomycetales*	*Demequinaceae*	*Demequina*	0	0	7,20861 E-07	0	0
Bacteria	*Firmicutes*	*Negativicutes*	*Selenomonadales*	*Veillonellaceae*	*Dendrosporobact er*	0	0	0	1,20806 E-06	0
Bacteria	*Actinobacteria*	*Actinobacteria*	*Actinomycetales*	*Dermacoccaceae*	*Dermacoccus*	0	0	2,09912 E-06	0	0
Bacteria	*Firmicutes*	*Bacilli*	*Lactobacillales*	*Carnobacteriaceae*	*Desemzia*	0	0	4,50781 E-07	3,24821 E-06	0
Bacteria	*Proteobacteria*	*Deltaproteobacteria*	*Desulfobacterales*	*Desulfobacteraceae*	*Desulfatitalea*	0	3,25208E-06	2,08693 E-05	3,23593 E-07	0
Bacteria	*Proteobacteria*	*Deltaproteobacteria*	*Desulfobacterales*	*Desulfobulbaceae*	*Desulfobulbus*	0	0	0	6,95456 E-07	0
Bacteria	*Proteobacteria*	*Deltaproteobacteria*	*Desulfovibrionales*	*Desulfomicrobiaceae*	*Desulfomicrobiu m*	0	2,52479E-06	0	2,38682 E-06	0
Bacteria	*Proteobacteria*	*Deltaproteobacteria*	*Desulfovibrionales*	*Desulfohalobiaceae*	*Desulfovermicul us*	0	0	5,2948E -07	0	0
Bacteria	*Proteobacteria*	*Deltaproteobacteria*	*Desulfovibrionales*	*Desulfovibrionaceae*	*Desulfovibrio*	0,00049 4569	0,0022286 49	0,00264 1559	0,00099 132	0,001891 364
Bacteria	*Firmicutes*	*Clostridia*	*Clostridiales*	*Incertae Sedis XI*	*Dethiosulfatibact er*	0	0	1,61593 E-06	5,2161E -05	0

Bacteria	*Proteobacteria*	*Alphaproteobacteria*	*Rhizobiales*	*Hyphomicrobiaceae*	*Devosia*	0	0	9,8853E-06	1,69077E-06	0
Bacteria	*Firmicutes*	*Negativicutes*	*Selenomonadales*	*Veillonellaceae*	*Dialister*	0,004166014	0,002024378	0,000729352	2,03534E-05	1,46139E-06
Bacteria	*Actinobacteria*	*Actinobacteria*	*Actinomycetales*	*Dietziaceae*	*Dietzia*	1,07312E-05	9,61809E-07	3,2231E-05	1,23742E-06	2,07444E-05
Bacteria	*Proteobacteria*	*Gammaproteobacteria*	*Legionellales*	*Coxiellaceae*	*Diplorickettsia*	0	0	0	0	1,29525E-06
Bacteria	*Proteobacteria*	*Gammaproteobacteria*	*Xanthomonadales*	*Xanthomonadaceae*	*Dokdonella*	0	0	0	4,17777E-07	0
Bacteria	*Firmicutes*	*Bacilli*	*Lactobacillales*	*Carnobacteriaceae*	*Dolosigranulum*	2,94302E-06	5,16903E-06	0	0	5,80361E-06
Bacteria	*Proteobacteria*	*Alphaproteobacteria*	*Rhodospirillales*	*Rhodospirillaceae*	*Dongia*	0	0	2,84082E-06	0	5,32275E-06
Bacteria	*Firmicutes*	*Clostridia*	*Clostridiales*	*Lachnospiraceae*	*Dorea*	0,00114792	0,00087763	0,001033214	0,00111602	0,001553794
Bacteria	*Proteobacteria*	*Betaproteobacteria*	*Burkholderiales*	*Oxalobacteraceae*	*Duganella*	0	0	1,56282E-07	0	0
Bacteria	*Proteobacteria*	*Gammaproteobacteria*	*Xanthomonadales*	*Xanthomonadaceae*	*Dyella*	0	0	0	0	1,15571E-05
Bacteria	*Bacteroidetes*	*Bacteroidia*	*Bacteroidales*	*Porphyromonadaceae*	*Dysgonomonas*	0	0	2,89086E-06	1,06203E-06	0
Bacteria	*Acidobacteria*	*Acidobacteria_Gp1*	*Edaphobacter*	*Edaphobacter*	*Edaphobacter*	0	0	4,65595E-07	0	0
Bacteria	*Actinobacteria*	*Actinobacteria*	*Coriobacteriales*	*Coriobacteriaceae*	*Eggerthella*	0	0	8,58518E-06	0,000122553	2,90426E-05
Bacteria	*Elusimicrobia*	*Elusimicrobia*	*Elusimicrobiales*	*Elusimicrobiaceae*	*Elusimicrobium*	0,006730554	0,001870505	0,000328789	0	0,008924486
Bacteria	*Bacteroidetes*	*Flavobacteriia*	*Flavobacteriales*	*Flavobacteriaceae*	*Empedobacter*	0	0	5,71263E-06	0	5,00045E-07
Bacteria	*Proteobacteria*	*Gammaproteobacteria*	*Pseudomonadales*	*Moraxellaceae*	*Enhydrobacter*	1,2035E-05	4,63257E-06	1,47462E-05	8,43151E-06	7,13396E-06
Bacteria	*Proteobacteria*	*Gammaproteobacteria*	*Enterobacteriales*	*Enterobacteriaceae*	*Enterobacter*	6,8467E-07	2,40151E-06	0,000448837	9,31626E-06	5,22647E-05
Bacteria	*Firmicutes*	*Bacilli*	*Lactobacillales*	*Enterococcaceae*	*Enterococcus*	5,05272E-06	1,30262E-06	2,30364E-05	1,49512E-06	0,000553352
Bacteria	*Actinobacteria*	*Actinobacteria*	*Coriobacteriales*	*Coriobacteriaceae*	*Enterorhabdus*	4,56347E-05	1,28947E-05	0,000581432	1,70568E-05	1,17942E-05
Bacteria	*Bacteroidetes*	*Flavobacteriia*	*Flavobacteriales*	*Flavobacteriaceae*	*Epilithonimonas*	0	0	0	0	8,22341E-07

Bacteria	*Firmicutes*	*Erysipelotrichia*	*Erysipelotrichales*	*Erysipelotrichaceae*	*Erysipelotrichaceae_incertae_sedis*	0,002981668	0,00040828	0,00026959	0,000382323	0,000227416
Bacteria	*Proteobacteria*	*Gammaproteobacteria*	*Enterobacteriales*	*Enterobacteriaceae*	*Escherichia/Shigella*	0,000891106	0,00046004	0,027220755	0,00084495	0,002297988
Bacteria	*Firmicutes*	*Clostridia*	*Clostridiales*	*Ruminococcaceae*	*Ethanoligenens*	0,000589745	0,000616109	0,000496218	8,69854E-05	0,000653798
Bacteria	*Firmicutes*	*Clostridia*	*Clostridiales*	*Eubacteriaceae*	*Eubacterium*	0,00282379	0,00401786	0,00761159	0,002586874	0,001832123
Bacteria	*Actinobacteria*	*Actinobacteria*	*Euzebyales*	*Euzebyaceae*	*Euzebya*	0	0	3,52987E-07	0	0
Bacteria	*Firmicutes*	*Bacilli*	*Bacillales*	*Bacillales_Incertae Sedis XII*	*Exiguobacterium*	0	0	5,82297E-06	0	0
Bacteria	*Firmicutes*	*Bacilli*	*Lactobacillales*	*Aerococcaceae*	*Facklamia*	0	0	2,54835E-06	3,88729E-06	1,99107E-05
Bacteria	*Firmicutes*	*Clostridia*	*Clostridiales*	*Ruminococcaceae*	*Faecalibacterium*	0,020893011	0,014153137	0,002024029	0,013052958	0,00246611
Bacteria	*Firmicutes*	*Bacilli*	*Bacillales*	*Bacillaceae 1*	*Falsibacillus*	0	0	6,19072E-06	0	1,727E-06
Bacteria	*Firmicutes*	*Clostridia*	*Clostridiales*	*Ruminococcaceae*	*Fastidiosipila*	2,07419E-06	3,50262E-06	2,03609E-06	3,5401E-07	5,43731E-06
Bacteria	*Bacteroidetes*	*Sphingobacteriia*	*Sphingobacteriales*	*Chitinophagaceae*	*Ferruginibacter*	0	0	1,37772E-06	0	0
Bacteria	*Firmicutes*	*Clostridia*	*Clostridiales*	*Clostridiaceae 1*	*Fervidicella*	0	0	3,17688E-06	0	0
Bacteria	*Fibrobacteres*	*Fibrobacteria*	*Fibrobacterales*	*Fibrobacteraceae*	*Fibrobacter*	0	0,000746676	0,00226435	0,00128398	0,000893402
Bacteria	*Firmicutes*	*Clostridia*	*Clostridiales*	*Clostridiales_Incertae Sedis XI*	*Finegoldia*	4,69713E-05	1,77562E-05	2,32134E-06	2,00574E-06	9,76735E-06
Bacteria	*Bacteroidetes*	*Sphingobacteriia*	*Sphingobacteriales*	*Chitinophagaceae*	*Flavisolibacter*	0	0	2,48547E-06	1,15619E-06	7,30695E-07
Bacteria	*Bacteroidetes*	*Flavobacteriia*	*Flavobacteriales*	*Flavobacteriaceae*	*Flavobacterium*	1,15613E-06	0	2,48123E-06	1,27858E-06	7,64216E-06
Bacteria	*Firmicutes*	*Clostridia*	*Clostridiales*	*Ruminococcaceae*	*Flavonifractor*	0,003022801	0,005291291	0,003955598	0,002171858	0,001563069
Bacteria	*Bacteroidetes*	*Flavobacteriia*	*Flavobacteriales*	*Cryomorphaceae*	*Fluviicola*	0	0	0	7,63737E-06	0
Bacteria	*Actinobacteria*	*Actinobacteria*	*Actinomycetales*	*Intrasporangiaceae*	*Fodinibacter*	0	0	1,92724E-07	0	0

Bacteria	*Firmicutes*	*Bacilli*	*Bacillales*	*Paenibacillaceae 1*	*Fontibacillus*	0	0	0	3,5401E-07	0
Bacteria	*Actinobacteria*	*Actinobacteria*	*Actinomycetales*	*Propionibacteriaceae*	*Friedmanniella*	0	0	6,2513E-07	3,64645E-07	0
Bacteria	*Proteobacteria*	*Gammaproteobacteria*	*Xanthomonadales*	*Xanthomonadaceae*	*Fulvimonas*	0	0	0	0	8,50838E-07
Bacteria	*Firmicutes*	*Clostridia*	*Clostridiales*	*Clostridiales_Incertae Sedis XII*	*Fusibacter*	1,4002E-05	5,64495E-06	7,98416E-06	3,66202E-06	0,000265393
Bacteria	*Fusobacteria*	*Fusobacteriia*	*Fusobacteriales*	*Fusobacteriaceae*	*Fusobacterium*	7,04847E-07	4,88868E-06	0,000452002	6,30609E-05	1,4036E-05
Bacteria	*Actinobacteria*	*Actinobacteria*	*Gaiellales*	*Gaiellaceae*	*Gaiella*	0	0	2,32654E-06	1,74562E-06	2,02595E-06
Bacteria	*Bacteroidetes*	*Flavobacteriia*	*Flavobacteriales*	*Flavobacteriaceae*	*Galbibacter*	0	2,10778E-06	3,04917E-07	0	0
Bacteria	*Proteobacteria*	*Gammaproteobacteria*	*Pasteurellales*	*Pasteurellaceae*	*Gallibacterium*	0	0	0	0	7,45147E-06
Bacteria	*Firmicutes*	*Clostridia*	*Clostridiales*	*Clostridiales_Incertae Sedis XI*	*Gallicola*	0	0	5,44817E-07	0	0
Bacteria	*Actinobacteria*	*Actinobacteria*	*Bifidobacteriales*	*Bifidobacteriaceae*	*Gardnerella*	0	0	0	0	4,3175E-07
Bacteria	*Bacteroidetes*	*Flavobacteriia*	*Flavobacteriales*	*Flavobacteriaceae*	*Gelidibacter*	0	0	0	2,16547E-07	0
Bacteria	*Firmicutes*	*Bacilli*	*Bacillales*	*Bacillales_Incertae Sedis XI*	*Gemella*	2,1233E-06	0	7,89213E-07	0	8,10378E-06
Bacteria	*Planctomycetes*	*Planctomycetia*	*Planctomycetales*	*Planctomycetaceae*	*Gemmata*	0	0	7,94768E-07	0	4,38417E-06
Bacteria	*Gemmatimonadetes*	*Gemmatimonadetes*	*Gemmatimonadales*	*Gemmatimonadaceae*	*Gemmatimonas*	0	0	6,20688E-06	2,12386E-06	8,22341E-07
Bacteria	*Firmicutes*	*Clostridia*	*Clostridiales*	*Ruminococcaceae*	*Gemmiger*	0,001094628	0,002945363	0,000176864	0,003669146	9,31094E-05
Bacteria	*Proteobacteria*	*Alphaproteobacteria*	*Rhodobacterales*	*Rhodobacteraceae*	*Gemmobacter*	0	0	1,5026E-07	2,16547E-07	0
Bacteria	*Firmicutes*	*Bacilli*	*Bacillales*	*Bacillaceae 1*	*Geobacillus*	0	0	3,52987E-07	0	0
Bacteria	*Actinobacteria*	*Actinobacteria*	*Actinomycetales*	*Geodermatophilaceae*	*Geodermatophilus*	0	0	6,42088E-07	0	0
Bacteria	*Proteobacteria*	*Deltaproteobacteria*	*Desulfuromonadales*	*Geobacteraceae*	*Geopsychrobacter*	0	0	1,15357E-06	0	0
Bacteria	*Actinobacteria*	*Actinobacteria*	*Actinomycetales*	*Bogoriellaceae*	*Georgenia*	0	0	6,67018E-07	0	0

Bacteria	*Firmicutes*	*Clostridia*	*Clostridiales*	*Clostridiaceae 4*	*Geosporobacter*	0	0	1,76493 E-07	0	0
Bacteria	*Proteobacteria*	*Gammaproteobacteria*	*Enterobacteriales*	*Enterobacteriaceae*	*Gibbsiella*	0	0	1,0548E -06	2,89771 E-07	0
Bacteria	*Bacteroidetes*	*Flavobacteriia*	*Flavobacteriales*	*Flavobacteriaceae*	*Gillisia*	7,04847 E-07	0	0	1,33681 E-06	0
Bacteria	*Firmicutes*	*Bacilli*	*Lactobacillales*	*Aerococcaceae*	*Globicatella*	0	0	1,92724 E-07	7,0802E -07	4,11171E -06
Bacteria	*Proteobacteria*	*Alphaproteobacteria*	*Rhodospirillales*	*Acetobacteraceae*	*Gluconacetobact er*	0	0	1,23725 E-06	0	0
Bacteria	*Proteobacteria*	*Alphaproteobacteria*	*Rhodospirillales*	*Acetobacteraceae*	*Gluconobacter*	0	0	9,88173 E-06	0	0
Bacteria	*Actinobacteria*	*Actinobacteria*	*Actinomycetales*	*Nocardiaceae*	*Gordonia*	0	0	2,24865 E-06	0	2,71819E -05
Bacteria	*Actinobacteria*	*Actinobacteria*	*Coriobacteriales*	*Coriobacteriaceae*	*Gordonibacter*	0,00046 0489	1,30262E-06	0,00013 8141	0,00039 377	3,48302E -05
Bacteria	*Acidobacteria*	*Acidobacteria_Gp1*	*Gp1*	*Gp1*	*Gp1*	0	0	9,37695 E-07	0	0
Bacteria	*Acidobacteria*	*Acidobacteria_Gp16*	*Gp16*	*Gp16*	*Gp16*	0	0	1,10742 E-06	0	8,635E-07
Bacteria	*Acidobacteria*	*Acidobacteria_Gp2*	*Gp2*	*Gp2*	*Gp2*	0	0	8,7086E -07	0	1,55733E -05
Bacteria	*Acidobacteria*	*Acidobacteria_Gp3*	*Gp3*	*Gp3*	*Gp3*	0	0	9,698E-07	5,56336 E-07	0
Bacteria	*Acidobacteria*	*Acidobacteria_Gp4*	*Gp4*	*Gp4*	*Gp4*	6,51926 E-06	0	2,17315 E-06	8,46107 E-07	0
Bacteria	*Acidobacteria*	*Acidobacteria_Gp6*	*Gp6*	*Gp6*	*Gp6*	2,31227 E-06	0	5,80644 E-06	0	0
Bacteria	*Acidobacteria*	*Acidobacteria_Gp7*	*Gp7*	*Gp7*	*Gp7*	0	0	7,53505 E-07	4,50315 E-07	0
Bacteria	*Cyanobacteria/ Chloroplast*	*Cyanobacteria*	*Family II*	*Family II*	*GpIIa*	0	0	0	8,20773 E-07	0
Bacteria	*Cyanobacteria/ Chloroplast*	*Cyanobacteria*	*Family XIII*	*Family XIII*	*GpXIII*	0	0	1,56282 E-07	4,33095 E-07	0
Bacteria	*Firmicutes*	*Clostridia*	*Clostridiales*	*Gracilibacteraceae*	*Gracilibacter*	2,59603 E-05	1,67093E-05	9,852E-06	9,28636 E-05	1,32239E -06
Bacteria	*Firmicutes*	*Bacilli*	*Lactobacillales*	*Carnobacteriaceae*	*Granulicatella*	2,85524 E-06	0	1,66935 E-07	0	7,31443E -06
Bacteria	*Acidobacteria*	*Acidobacteria_Gp1*	*Granulicella*	*Granulicella*	*Granulicella*	0	0	0	5,78093 E-07	0

Bacteria	*Firmicutes*	*Clostridia*	*Clostridiales*	*Clostridiales_Incertae Sedis XII*	*Guggenheimella*	0	0	3,25484 E-06	5,19945 E-06	2,46702E -06
Bacteria	*Proteobacteria*	*Gammaproteobacteria*	*Pasteurellales*	*Pasteurellaceae*	*Haemophilus*	4,38971 E-06	5,77586E-06	1,54534 E-06	0	1,53906E -05
Bacteria	*Bacteroidetes*	*Sphingobacteriia*	*Sphingobacteriales*	*Saprospiraceae*	*Haliscomenobacter*	0	0	0	9,70778 E-07	0
Bacteria	*Bacteroidetes*	*Bacteroidia*	*Bacteroidales*	*Prevotellaceae*	*Hallella*	0,00058 0152	0,0034289 08	0,00216 3944	0,00103 5796	0,001789 438
Bacteria	*Firmicutes*	*Bacilli*	*Bacillales*	*Bacillaceae 2*	*Halobacillus*	0	0	1,27464 E-07	0	0
Bacteria	*Tenericutes*	*Mollicutes*	*Haloplasmatales*	*Haloplasmataceae*	*Haloplasma*	0	0	0	0	1,25726E -06
Bacteria	*Proteobacteria*	*Alphaproteobacteria*	*Rhizobiales*	*Methylocystaceae*	*Hansschlegelia*	0	0	0	5,56336 E-07	0
Bacteria	*Actinobacteria*	*Actinobacteria*	*Actinomycetales*	*Dermabacteraceae*	*Helcobacillus*	0	0	1,80215 E-07	0	0
Bacteria	*Firmicutes*	*Clostridia*	*Clostridiales*	*Clostridiales_Incertae Sedis XI*	*Helcococcus*	0	0	3,65559 E-06	1,33681 E-06	1,21019E -05
Bacteria	*Proteobacteria*	*Epsilonproteobacteria*	*Campylobacterales*	*Helicobacteraceae*	*Helicobacter*	8,35086 E-07	0,0003073 59	0,00020 4999	0,00068 5216	0
Bacteria	*Proteobacteria*	*Betaproteobacteria*	*Burkholderiales*	*Oxalobacteraceae*	*Herbaspirillum*	7,38594 E-07	0	0	0	0
Bacteria	*Firmicutes*	*Clostridia*	*Clostridiales*	*Lachnospiraceae*	*Hespellia*	8,0461E -06	5,03056E-06	1,39726 E-05	1,29351 E-05	5,76153E -06
Bacteria	*Firmicutes*	*Erysipelotrichia*	*Erysipelotrichales*	*Erysipelotrichaceae*	*Holdemania*	8,48859 E-06	0,0002077 62	0,00312 098	0,00919 5056	0,001775 065
Bacteria	*Firmicutes*	*Clostridia*	*Clostridiales*	*Lachnospiraceae*	*Howardella*	0	0	1,14921 E-05	1,73963 E-06	3,09055E -06
Bacteria	*Proteobacteria*	*Epsilonproteobacteria*	*Campylobacterales*	*Hydrogenimonaceae*	*Hydrogenimonas*	0	0	1,80215 E-07	0	0
Bacteria	*Firmicutes*	*Clostridia*	*Clostridiales*	*Ruminococcaceae*	*Hydrogenoanaerobacterium*	0,00050 7878	0,0003172 26	0,00053 868	0,00014 0839	0,000334 361
Bacteria	*Proteobacteria*	*Betaproteobacteria*	*Burkholderiales*	*Comamonadaceae*	*Hydrogenophaga*	0	0	1,56282 E-07	0	0
Bacteria	*Bacteroidetes*	*Cytophagia*	*Cytophagales*	*Cytophagaceae*	*Hymenobacter*	0	0	1,5851E -06	1,19764 E-06	0
Bacteria	*Proteobacteria*	*Alphaproteobacteria*	*Rhizobiales*	*Hyphomicrobiaceae*	*Hyphomicrobium*	0	0	0	0	8,635E-07
Bacteria	*Actinobacteria*	*Actinobacteria*	*Acidimicrobiales*	*Iamiaceae*	*Iamia*	0	0	9,60051 E-07	0	0

Bacteria	*Actinobacteria*	*Actinobacteria*	*Acidimicrobiales*	*Acidimicrobiaceae*	*Ilumatobacter*	5,78067E-06	0	5,82098E-07	3,64645E-07	1,46139E-06
Bacteria	*Proteobacteria*	*Alphaproteobacteria*	*Rhodospirillales*	*Rhodospirillaceae*	*Insolitispirillum*	8,01297E-05	0,000152312	4,55949E-05	2,57674E-05	0,000910066
Bacteria	*Firmicutes*	*Bacilli*	*Lactobacillales*	*Carnobacteriaceae*	*Isobaculum*	0	0	1,92029E-07	0	4,62023E-06
Bacteria	*Proteobacteria*	*Betaproteobacteria*	*Burkholderiales*	*Oxalobacteraceae*	*Janthinobacterium*	0	0	0	2,16547E-07	1,00009E-06
Bacteria	*Firmicutes*	*Bacilli*	*Bacillales*	*Planococcaceae*	*Jeotgalibacillus*	0	0	1,02325E-06	9,208E-06	0
Bacteria	*Firmicutes*	*Bacilli*	*Bacillales*	*Staphylococcaceae*	*Jeotgalicoccus*	0	0	2,49322E-07	0	4,38625E-06
Bacteria	*Synergistetes*	*Synergistia*	*Synergistales*	*Synergistaceae*	*Jonquetella*	0	1,36064E-06	0	1,51567E-06	0
Bacteria	*Proteobacteria*	*Alphaproteobacteria*	*Rhizobiales*	*Rhizobiaceae*	*Kaistia*	0	1,30262E-06	0	0	0
Bacteria	*Firmicutes*	*Erysipelotrichia*	*Erysipelotrichales*	*Erysipelotrichaceae*	*Kandleria*	0	0	0,000530524	0,000215254	1,82418E-05
Bacteria	*Proteobacteria*	*Betaproteobacteria*	*Burkholderiales*	*Alcaligenaceae*	*Kerstersia*	0	0	2,40417E-06	4,70618E-06	0
Bacteria	*Proteobacteria*	*Alphaproteobacteria*	*Kiloniellales*	*Kiloniellaceae*	*Kiloniella*	0	0	0	2,20656E-05	0
Bacteria	*Actinobacteria*	*Actinobacteria*	*Actinomycetales*	*Kineosporiaceae*	*Kineococcus*	0	0	7,3838E-07	5,56336E-07	0
Bacteria	*Actinobacteria*	*Actinobacteria*	*Actinomycetales*	*Kineosporiaceae*	*Kineosporia*	0	0	0	6,2012E-07	0
Bacteria	*Proteobacteria*	*Betaproteobacteria*	*Neisseriales*	*Neisseriaceae*	*Kingella*	0	0	1,09398E-06	1,25333E-06	0
Bacteria	*Proteobacteria*	*Betaproteobacteria*	*Burkholderiales*	*Comamonadaceae*	*Kinneretia*	0	0	0	0	8,38176E-07
Bacteria	*Proteobacteria*	*Gammaproteobacteria*	*Enterobacteriales*	*Enterobacteriaceae*	*Klebsiella*	0	0	0,000200718	2,90311E-05	0
Bacteria	*Actinobacteria*	*Actinobacteria*	*Actinomycetales*	*Micrococcaceae*	*Kocuria*	0	0	3,69217E-07	1,08274E-06	1,16245E-06
Bacteria	*Proteobacteria*	*Deltaproteobacteria*	*Myxococcales*	*Kofleriaceae*	*Kofleria*	0	0	3,12565E-07	0	0
Bacteria	*Proteobacteria*	*Gammaproteobacteria*	*Enterobacteriales*	*Enterobacteriaceae*	*Kosakonia*	0	0	2,32439E-06	0	9,18743E-06
Bacteria	*Actinobacteria*	*Actinobacteria*	*Actinomycetales*	*Nocardioidaceae*	*Kribbella*	0	0	0	2,89771E-07	0

Bacteria	*Firmicutes*	*Bacilli*	*Bacillales*	*Planococcaceae*	*Kurthia*	0	2,72128E-06	3,18267E-05	3,0813E-06	2,59548E-06
Bacteria	*Proteobacteria*	*Alphaproteobacteria*	*Rhizobiales*	*Xanthobacteraceae*	*Labrys*	0	0	3,6043E-07	4,02996E-07	0
Bacteria	*Firmicutes*	*Clostridia*	*Clostridiales*	*Lachnospiraceae*	*Lachnoanaerobaculum*	0,000225738	0,000202376	0,000156694	9,78319E-05	9,64775E-05
Bacteria	*Firmicutes*	*Clostridia*	*Clostridiales*	*Lachnospiraceae*	*Lachnobacterium*	2,92063E-06	7,4032E-06	1,53938E-06	2,89878E-05	4,71505E-06
Bacteria	*Firmicutes*	*Clostridia*	*Clostridiales*	*Lachnospiraceae*	*Lachnospira*	3,06258E-05	5,58094E-05	1,31516E-05	5,02513E-05	5,73935E-05
Bacteria	*Firmicutes*	*Clostridia*	*Clostridiales*	*Lachnospiraceae*	*Lachnospiracea_incertae_sedis*	0,027238906	0,037117376	0,04302749	0,019216133	0,022422667
Bacteria	*Firmicutes*	*Bacilli*	*Lactobacillales*	*Lactobacillaceae*	*Lactobacillus*	5,3964E-05	0,004839212	0,012037922	0,000620451	0,000450592
Bacteria	*Firmicutes*	*Bacilli*	*Lactobacillales*	*Streptococcaceae*	*Lactococcus*	1,06165E-06	0	7,25384E-06	3,5401E-07	0
Bacteria	*Firmicutes*	*Clostridia*	*Clostridiales*	*Lachnospiraceae*	*Lactonifactor*	4,27439E-05	7,05285E-05	0,000117616	9,44071E-05	2,52434E-05
Bacteria	*Firmicutes*	*Bacilli*	*Lactobacillales*	*Streptococcaceae*	*Lactovum*	0	1,92362E-06	1,3919E-06	2,78601E-06	2,47217E-06
Bacteria	*Proteobacteria*	*Deltaproteobacteria*	*Desulfovibrionales*	*Desulfovibrionaceae*	*Lawsonia*	0	0	0	4,17777E-07	0
Bacteria	*Proteobacteria*	*Gammaproteobacteria*	*Enterobacteriales*	*Enterobacteriaceae*	*Leclercia*	0	0	8,82715E-06	2,16547E-07	0
Bacteria	*Proteobacteria*	*Gammaproteobacteria*	*Legionellales*	*Legionellaceae*	*Legionella*	0	1,30262E-06	4,31831E-07	0	0
Bacteria	*Actinobacteria*	*Actinobacteria*	*Actinomycetales*	*Microbacteriaceae*	*Leifsonia*	0	0	0	4,50315E-07	0
Bacteria	*Proteobacteria*	*Gammaproteobacteria*	*Enterobacteriales*	*Enterobacteriaceae*	*Lelliottia*	0	0	2,49322E-07	0	0
Bacteria	*Fusobacteria*	*Fusobacteriia*	*Fusobacteriales*	*Leptotrichiaceae*	*Leptotrichia*	1,06266E-06	2,10778E-06	3,90107E-07	0	0
Bacteria	*Actinobacteria*	*Actinobacteria*	*Actinomycetales*	*Microbacteriaceae*	*Leucobacter*	0	0	3,07926E-06	0	0
Bacteria	*Firmicutes*	*Bacilli*	*Lactobacillales*	*Leuconostocaceae*	*Leuconostoc*	0	3,82742E-06	3,92192E-06	0	0
Bacteria	*Proteobacteria*	*Betaproteobacteria*	*Burkholderiales*	*Burkholderiaceae*	*Limnobacter*	0	1,14513E-06	3,11916E-06	0	0
Bacteria	*Chloroflexi*	*Caldilineae*	*Caldilineales*	*Caldilineaceae*	*Litorilinea*	4,62453E-06	0	0	0	0

Bacteria	*Bacteroidetes*	*Flavobacteriia*	*Flavobacteriales*	*Flavobacteriaceae*	*Lutaonella*	0	0	1,92724 E-07	0	2,00018E -06
Bacteria	*Proteobacteria*	*Gammaproteobacteria*	*Xanthomonadales*	*Xanthomonadaceae*	*Luteimonas*	0	0	6,18539 E-07	1,51583 E-06	2,93361E -06
Bacteria	*Verrucomicrobi a*	*Verrucomicrobiae*	*Verrucomicrobiales*	*Verrucomicrobiaceae*	*Luteolibacter*	0	0	2,49322 E-07	2,16547 E-07	0
Bacteria	*Firmicutes*	*Clostridia*	*Clostridiales*	*Gracilibacteraceae*	*Lutispora*	0,00063 2364	0,0005287 61	0,00051 1156	1,2221E -06	0,001658 782
Bacteria	*Firmicutes*	*Bacilli*	*Bacillales*	*Planococcaceae*	*Lysinibacillus*	6,72584 E-06	0,0001742 03	0,00195 8194	0,00146 0519	0,000377 528
Bacteria	*Proteobacteria*	*Gammaproteobacteria*	*Xanthomonadales*	*Xanthomonadaceae*	*Lysobacter*	0	0	2,41604 E-06	7,9464E -07	0
Bacteria	*Firmicutes*	*Bacilli*	*Bacillales*	*Staphylococcaceae*	*Macrococcus*	0	0	1,26613 E-06	0	0
Bacteria	*Proteobacteria*	*Betaproteobacteria*	*Burkholderiales*	*Comamonadaceae*	*Macromonas*	0	0	0	9,70778 E-07	0
Bacteria	*Proteobacteria*	*Alphaproteobacteria*	*Rhodospirillales*	*Rhodospirillaceae*	*Magnetospirillu m*	0	0	8,35085 E-07	0	2,19209E -06
Bacteria	*Bacteroidetes*	*Bacteroidia*	*Bacteroidales*	*Marinilabiliaceae*	*Mangroviflexus*	0	0	0	4,70618 E-07	1,7192E-05
Bacteria	*Bacteroidetes*	*Bacteroidetes"_incertae _sedis*	*Marinifilum*	*Marinifilum*	*Marinifilum*	0	0	0	0	4,3175E-07
Bacteria	*Actinobacteria*	*Actinobacteria*	*Actinomycetales*	*Nocardioidaceae*	*Marmoricola*	4,62453 E-06	0	4,08185 E-06	1,7576E -06	0
Bacteria	*Firmicutes*	*Clostridia*	*Clostridiales*	*Lachnospiraceae*	*Marvinbryantia*	0	9,62643E-07	3,68667 E-06	1,41604 E-06	4,3175E-07
Bacteria	*Proteobacteria*	*Betaproteobacteria*	*Burkholderiales*	*Oxalobacteraceae*	*Massilia*	0	2,88793E-06	2,01687 E-06	2,83424 E-06	3,37168E -06
Bacteria	*Firmicutes*	*Negativicutes*	*Selenomonadales*	*Veillonellaceae*	*Megamonas*	0	0	2,38598 E-06	0	4,70771E -05
Bacteria	*Firmicutes*	*Negativicutes*	*Selenomonadales*	*Veillonellaceae*	*Megasphaera*	4,54875 E-05	0,0003254 54	0,00478 0706	0,00050 0667	9,90526E -06
Bacteria	*Deinococcus-Thermus*	*Deinococci*	*Thermales*	*Thermaceae*	*Meiothermus*	0	0	0	0	2,15875E -06
Bacteria	*Proteobacteria*	*Deltaproteobacteria*	*Myxococcales*	*Cystobacteraceae*	*Melittangium*	0	0	0	5,79542 E-07	0
Bacteria	*Bacteroidetes*	*Cytophagia*	*Cytophagales*	*Cytophagaceae*	*Meniscus*	1,36934 E-06	1,07943E-06	2,63812 E-06	3,76789 E-05	4,19088E -07
Bacteria	*Proteobacteria*	*Alphaproteobacteria*	*Rhizobiales*	*Phyllobacteriaceae*	*Mesorhizobium*	0	0	1,0597E -06	1,66107 E-06	7,30943E -06

Archaea	*Euryarchaeota*	*Methanobacteria*	*Methanobacteriales*	*Methanobacteriaceae*	*Methanobrevibacter*	0	0,001601267	0,00532259	0,000189169	1,30821E-05
Archaea	*Euryarchaeota*	*Methanobacteria*	*Methanobacteriales*	*Methanobacteriaceae*	*Methanosphaera*	0	0,000208958	7,67577E-07	3,03151E-05	0
Bacteria	*Proteobacteria*	*Betaproteobacteria*	*Burkholderiales*	*Burkholderiales_incertae_sedis*	*Methylibium*	0	0	1,29333E-06	0	0
Bacteria	*Proteobacteria*	*Alphaproteobacteria*	*Rhizobiales*	*Methylobacteriaceae*	*Methylobacterium*	0	0	1,78684E-05	2,36234E-06	5,06704E-06
Bacteria	*Proteobacteria*	*Alphaproteobacteria*	*Rhizobiales*	*Methylocystaceae*	*Methylocystis*	0	0	4,59573E-07	0	0
Bacteria	*Proteobacteria*	*Betaproteobacteria*	*Methylophilales*	*Methylophilaceae*	*Methylophilus*	0	0	7,74293E-06	0	0
Bacteria	*Proteobacteria*	*Alphaproteobacteria*	*Rhizobiales*	*Beijerinckiaceae*	*Methylorosula*	0	0	1,56282E-07	0	0
Bacteria	*Actinobacteria*	*Actinobacteria*	*Actinomycetales*	*Microbacteriaceae*	*Microbacterium*	1,82634E-06	1,17934E-06	5,71616E-06	2,82371E-06	3,42868E-06
Bacteria	*Actinobacteria*	*Actinobacteria*	*Actinomycetales*	*Microbacteriaceae*	*Microcella*	0	1,30262E-06	3,32594E-06	1,15175E-06	0
Bacteria	*Actinobacteria*	*Actinobacteria*	*Actinomycetales*	*Micrococcaceae*	*Micrococcus*	4,98125E-06	2,14198E-06	2,56681E-05	1,27858E-06	2,59695E-05
Bacteria	*Proteobacteria*	*Alphaproteobacteria*	*Rhizobiales*	*Methylobacteriaceae*	*Microvirga*	0	0	3,27279E-06	0	4,19088E-07
Bacteria	*Actinobacteria*	*Actinobacteria*	*Actinomycetales*	*Nocardiaceae*	*Millisia*	0	0	1,72201E-06	0	0
Bacteria	*Actinobacteria*	*Actinobacteria*	*Actinomycetales*	*Beutenbergiaceae*	*Miniimonas*	0	0	1,92724E-07	0	0
Bacteria	*Firmicutes*	*Negativicutes*	*Selenomonadales*	*Veillonellaceae*	*Mitsuokella*	6,8467E-07	0,000955482	0,003174335	0,000325221	0
Bacteria	*Actinobacteria*	*Actinobacteria*	*Actinomycetales*	*Actinomycetaceae*	*Mobiluncus*	0	0	2,41536E-06	1,56383E-06	4,19088E-07
Bacteria	*Actinobacteria*	*Actinobacteria*	*Actinomycetales*	*Geodermatophilaceae*	*Modestobacter*	0	0	0	2,16547E-07	8,635E-07
Bacteria	*Firmicutes*	*Clostridia*	*Clostridiales*	*Clostridiales_Incertae Sedis XIII*	*Mogibacterium*	4,84839E-05	9,73031E-05	0,000136971	5,84953E-05	0,00014638
Bacteria	*Bacteroidetes*	*Flavobacteriia*	*Flavobacteriales*	*Flavobacteriaceae*	*Moheibacter*	0	0	0	4,33095E-07	0
Bacteria	*Firmicutes*	*Clostridia*	*Clostridiales*	*Lachnospiraceae*	*Moryella*	0	0	5,08833E-07	0	0
Bacteria	*Deferribacteres*	*Deferribacteres*	*Deferribacterales*	*Deferribacteraceae*	*Mucispirillum*	0	5,59831E-05	5,09107E-05	0,000582846	0

Bacteria	*Firmicutes*	*Clostridia*	*Clostridiales*	*Incertae Sedis XI*	*Murdochiella*	0	0	1,98276 E-06	3,33417 E-06	0
Bacteria	*Actinobacteria*	*Actinobacteria*	*Actinomycetales*	*Microbacteriaceae*	*Mycetocola*	0	0	0	4,17777 E-07	0
Bacteria	*Actinobacteria*	*Actinobacteria*	*Actinomycetales*	*Mycobacteriaceae*	*Mycobacterium*	0	0	3,02927 E-06	6,09384 E-06	1,64468E -06
Bacteria	*Tenericutes*	*Mollicutes*	*Mycoplasmatales*	*Mycoplasmataceae*	*Mycoplasma*	7,38594 E-07	0	5,80791 E-05	7,10103 E-05	0,000388 092
Bacteria	*Actinobacteria*	*Actinobacteria*	*Actinomycetales*	*Nakamurellaceae*	*Nakamurella*	0	0	4,67221 E-07	4,02996 E-07	4,3175E-07
Bacteria	*Firmicutes*	*Clostridia*	*Clostridiales*	*Natranaerovirga*	*Natranaerovirga*	0	0	1,22257 E-06	3,23593 E-07	8,635E-07
Bacteria	*Proteobacteria*	*Betaproteobacteria*	*Burkholderiales*	*Oxalobacteraceae*	*Naxibacter*	0	0	5,21164 E-07	0	0
Bacteria	*Firmicutes*	*Negativicutes*	*Selenomonadales*	*Veillonellaceae*	*Negativicoccus*	5,3893E -06	0	0	0	0
Bacteria	*Proteobacteria*	*Betaproteobacteria*	*Neisseriales*	*Neisseriaceae*	*Neisseria*	2,80998 E-06	3,32911E-06	0,00016 9436	5,88337 E-06	4,6173E-05
Bacteria	*Chlamydiae*	*Chlamydiia*	*Chlamydiales*	*Parachlamydiaceae*	*Neochlamydia*	0	0	5,32654 E-07	0	0
Bacteria	*Actinobacteria*	*Actinobacteria*	*Actinomycetales*	*Micrococcaceae*	*Nesterenkonia*	0	0	8,85156 E-07	6,49642 E-07	1,24143E -06
Bacteria	*Proteobacteria*	*Gammaproteobacteria*	*Pasteurellales*	*Pasteurellaceae*	*Nicoletella*	0	0	0	0	8,95084E -06
Bacteria	*Nitrospirae*	*Nitrospira*	*Nitrospirales*	*Nitrospiraceae*	*Nitrospira*	0	0	2,1459E -06	0	0
Bacteria	*Actinobacteria*	*Actinobacteria*	*Actinomycetales*	*Nocardioidaceae*	*Nocardioides*	1,15613 E-06	0	7,41419 E-06	3,70531 E-06	0
Bacteria	*Firmicutes*	*Bacilli*	*Bacillales*	*Staphylococcaceae*	*Nosocomiicoccus*	0	0	0	0	5,00045E -07
Bacteria	*Proteobacteria*	*Alphaproteobacteria*	*Sphingomonadales*	*Sphingomonadaceae*	*Novosphingobiu m*	3,4684E -06	0	3,85009 E-06	1,05016 E-06	4,72121E -06
Bacteria	*Bacteroidetes*	*Sphingobacteriia*	*Sphingobacteriales*	*Sphingobacteriaceae*	*Nubsella*	0	0	0	0	4,80731E -06
Bacteria	*Firmicutes*	*Bacilli*	*Bacillales*	*Bacillaceae 2*	*Oceanobacillus*	0	0	0	2,16547 E-07	0
Bacteria	*Proteobacteria*	*Alphaproteobacteria*	*Rhizobiales*	*Brucellaceae*	*Ochrobactrum*	8,20734 E-06	1,07943E-06	3,99582 E-07	4,03688 E-06	6,30366E -06
Bacteria	*Bacteroidetes*	*Bacteroidia*	*Bacteroidales*	*Porphyromonadaceae*	*Odoribacter*	0	0	1,36659 E-06	0	3,36188E -05

Bacteria	*Bacteroidetes*	*Bacteroidetes"_incertae_sedis*	*Ohtaekwangia*	*Ohtaekwangia*	*Ohtaekwangia*	2,31227E-06	0	1,05189E-06	0	0
Bacteria	*Actinobacteria*	*Actinobacteria*	*Actinomycetales*	*Microbacteriaceae*	*Okibacterium*	0	0	0	0	2,00018E-06
Bacteria	*Proteobacteria*	*Betaproteobacteria*	*Burkholderiales*	*Alcaligenaceae*	*Oligella*	0	0	6,2513E-07	0	2,92278E-06
Bacteria	*Lentisphaerae*	*Oligosphaeria*	*Oligosphaerales*	*Oligosphaeraceae*	*Oligosphaera*	0	5,69725E-06	2,11151E-06	2,77339E-06	0
Bacteria	*Actinobacteria*	*Actinobacteria*	*Coriobacteriales*	*Coriobacteriaceae*	*Olsenella*	0,000152091	3,62157E-05	0,000351396	8,65292E-05	5,46609E-05
Bacteria	*Verrucomicrobia*	*Opitutae*	*Opitutales*	*Opitutaceae*	*Opitutus*	1,15613E-06	0	5,78172E-07	0	1,00009E-06
Bacteria	*Firmicutes*	*Clostridia*	*Clostridiales*	*Lachnospiraceae*	*Oribacterium*	0	0,000506462	0,000663296	0,005912754	0,000606877
Bacteria	*Actinobacteria*	*Actinobacteria*	*Actinomycetales*	*Intrasporangiaceae*	*Ornithinicoccus*	0	0	0	0	1,64468E-06
Bacteria	*Actinobacteria*	*Actinobacteria*	*Actinomycetales*	*Intrasporangiaceae*	*Ornithinimicrobium*	2,31227E-06	0	0,000134128	1,01119E-06	1,727E-06
Bacteria	*Bacteroidetes*	*Flavobacteriia*	*Flavobacteriales*	*Flavobacteriaceae*	*Ornithobacterium*	1,36934E-06	0,001895087	0,003260714	7,18769E-05	0,00706337
Bacteria	*Firmicutes*	*Clostridia*	*Clostridiales*	*Ruminococcaceae*	*Oscillibacter*	0,046339027	0,032909507	0,02158873	0,009584477	0,006200557
Bacteria	*Proteobacteria*	*Gammaproteobacteria*	*Pasteurellales*	*Pasteurellaceae*	*Otariodibacter*	0	0	0	6,77603E-07	0
Bacteria	*Bacteroidetes*	*Flavobacteriia*	*Flavobacteriales*	*Cryomorphaceae*	*Owenweeksia*	0	0	0	3,32093E-06	0
Bacteria	*Proteobacteria*	*Betaproteobacteria*	*Burkholderiales*	*Oxalobacteraceae*	*Oxalicibacterium*	0	0	1,56282E-07	0	0
Bacteria	*Proteobacteria*	*Betaproteobacteria*	*Burkholderiales*	*Oxalobacteraceae*	*Oxalobacter*	2,05401E-06	0,001337466	0,001264246	0,001195954	1,24143E-06
Bacteria	*Firmicutes*	*Bacilli*	*Bacillales*	*Paenibacillaceae 2*	*Oxalophagus*	0	1,36064E-06	2,49322E-07	0	0
Bacteria	*Firmicutes*	*Bacilli*	*Bacillales*	*Paenibacillaceae 1*	*Paenibacillus*	0	0	1,06982E-06	2,17747E-05	0
Bacteria	*Firmicutes*	*Bacilli*	*Bacillales*	*Planococcaceae*	*Paenisporosarcina*	0	0	0	6,06333E-06	0
Bacteria	*Proteobacteria*	*Gammaproteobacteria*	*Enterobacteriales*	*Enterobacteriaceae*	*Pantoea*	0	0	1,5026E-07	0	1,46139E-06
Bacteria	*Firmicutes*	*Clostridia*	*Clostridiales*	*Ruminococcaceae*	*Papillibacter*	0,002439223	0,000960806	0,004550016	0,00039259	0,002213555

Bacteria	*Bacteroidetes*	*Bacteroidia*	*Bacteroidales*	*Porphyromonadaceae*	*Parabacteroides*	0,005395332	0,004388671	0,00143434	0,000156735	0,003166421
Bacteria	*Chlamydiae*	*Chlamydiia*	*Chlamydiales*	*Parachlamydiaceae*	*Parachlamydia*	0	0	1,68908E-06	0	1,25726E-06
Bacteria	*Proteobacteria*	*Alphaproteobacteria*	*Rhodobacterales*	*Rhodobacteraceae*	*Paracoccus*	4,30156E-06	1,30262E-06	1,169E-05	6,66863E-07	7,65925E-06
Bacteria	*Actinobacteria*	*Actinobacteria*	*Coriobacteriales*	*Coriobacteriaceae*	*Paraeggerthella*	0	1,56077E-05	3,21033E-05	1,58223E-05	0
Bacteria	*Firmicutes*	*Bacilli*	*Lactobacillales*	*Lactobacillaceae*	*Paralactobacillus*	1,15613E-06	4,96498E-05	0,000106256	4,70618E-07	0
Bacteria	*Bacteroidetes*	*Sphingobacteriia*	*Sphingobacteriales*	*Sphingobacteriaceae*	*Parapedobacter*	0	0	7,03925E-07	0	1,46139E-06
Bacteria	*Bacteroidetes*	*Bacteroidia*	*Bacteroidales*	*Prevotellaceae*	*Paraprevotella*	0,011868633	0,011142106	0,0071648	0,030328039	0,016493814
Bacteria	*Bacteroidetes*	*Sphingobacteriia*	*Sphingobacteriales*	*Chitinophagaceae*	*Parasegetibacter*	0	0	0	9,00631E-07	1,29525E-06
Bacteria	*Firmicutes*	*Clostridia*	*Clostridiales*	*Lachnospiraceae*	*Parasporobacterium*	3,79669E-05	2,32361E-05	3,58116E-05	1,87918E-05	5,70204E-06
Bacteria	*Proteobacteria*	*Betaproteobacteria*	*Burkholderiales*	*Sutterellaceae*	*Parasutterella*	0,001429451	6,51723E-05	1,21803E-05	2,50666E-06	2,15206E-05
Bacteria	*Parcubacteria*	*Parcubacteria_genera_incertae_sedis*	*Parcubacteria_genera_incertae_sedis*	*Parcubacteria_genera_incertae_sedis*	*Parcubacteria_genera_incertae_sedis*	0	0	6,24915E-07	4,02996E-07	5,61184E-06
Bacteria	*Firmicutes*	*Clostridia*	*Clostridiales*	*Clostridiales_Incertae Sedis XI*	*Parvimonas*	0	0	3,2444E-06	0	0
Bacteria	*Proteobacteria*	*Gammaproteobacteria*	*Pasteurellales*	*Pasteurellaceae*	*Pasteurella*	0	0	2,99959E-05	3,46845E-06	2,08464E-05
Bacteria	*Actinobacteria*	*Actinobacteria*	*Solirubrobacterales*	*Patulibacteraceae*	*Patulibacter*	0	0	0	6,2012E-07	0
Bacteria	*Firmicutes*	*Bacilli*	*Bacillales*	*Bacillaceae 2*	*Paucisalibacillus*	0	0	1,76493E-07	2,16547E-07	0
Bacteria	*Firmicutes*	*Bacilli*	*Lactobacillales*	*Lactobacillaceae*	*Pediococcus*	0	2,3673E-06	2,8157E-07	0	2,5905E-06
Bacteria	*Bacteroidetes*	*Sphingobacteriia*	*Sphingobacteriales*	*Sphingobacteriaceae*	*Pedobacter*	0	0	0	6,49642E-07	0
Bacteria	*Proteobacteria*	*Alphaproteobacteria*	*Rhizobiales*	*Hyphomicrobiaceae*	*Pedomicrobium*	0	0	5,61887E-07	0	5,00045E-07
Bacteria	*Proteobacteria*	*Alphaproteobacteria*	*Rhizobiales*	*Hyphomicrobiaceae*	*Pelagibacterium*	0	0	1,40785E-07	0	0

Bacteria	*Proteobacteria*	*Betaproteobacteria*	*Burkholderiales*	*Alcaligenaceae*	*Pelistega*	0	0	3,39678 E-06	0	2,65414E -05
Bacteria	*Proteobacteria*	*Betaproteobacteria*	*Burkholderiales*	*Comamonadaceae*	*Pelomonas*	2,95738 E-06	2,44434E-06	7,3337E -07	0	1,70561E -05
Bacteria	*Firmicutes*	*Clostridia*	*Clostridiales*	*Syntrophomonadaceae*	*Pelospora*	1,35932 E-05	4,54903E-06	3,14114 E-05	2,7973E -05	1,92161E -05
Bacteria	*Firmicutes*	*Clostridia*	*Clostridiales*	*Peptococcaceae 1*	*Peptococcus*	0,00022 5542	0,0001304 71	0,00053 5722	0,00039 0157	0,001385 708
Bacteria	*Firmicutes*	*Clostridia*	*Clostridiales*	*Clostridiales_Incertae Sedis XI*	*Peptoniphilus*	3,46841 E-06	6,52626E-06	2,20799 E-06	1,44077 E-06	4,3175E-07
Bacteria	*Firmicutes*	*Clostridia*	*Clostridiales*	*Peptostreptococcaceae*	*Peptostreptococcaceae_incertae_sedis*	0	0	1,80215 E-07	0	0
Bacteria	*Firmicutes*	*Clostridia*	*Clostridiales*	*Peptostreptococcaceae*	*Peptostreptococcus*	0	0	4,40675 E-06	6,33659 E-06	0
Bacteria	*Proteobacteria*	*Deltaproteobacteria*	*Bdellovibrionales*	*Bacteriovoracaceae*	*Peredibacter*	0	0	1,66935 E-07	0	0
Bacteria	*Proteobacteria*	*Alphaproteobacteria*	*Rhodospirillales*	*Rhodospirillaceae*	*Phaeovibrio*	0	0	0	0	1,55304E -06
Bacteria	*Firmicutes*	*Negativicutes*	*Selenomonadales*	*Acidaminococcaceae*	*Phascolarctobacterium*	0,01062 2926	0,0325542 84	0,01655 29	0,04910 7779	0,004919 848
Bacteria	*Proteobacteria*	*Deltaproteobacteria*	*Myxococcales*	*Phaselicystidaceae*	*Phaselicystis*	0	0	1,29646 E-06	0	0
Bacteria	*Proteobacteria*	*Alphaproteobacteria*	*Caulobacterales*	*Caulobacteraceae*	*Phenylobacterium*	0	0	5,96312 E-07	0	1,54673E -05
Bacteria	*Bacteroidetes*	*Bacteroidia*	*Bacteroidales*	*Bacteroidales"_incertae_sedis*	*Phocaeicola*	0	0	1,12067 E-06	6,37546 E-05	0
Bacteria	*Actinobacteria*	*Actinobacteria*	*Actinomycetales*	*Intrasporangiaceae*	*Phycicoccus*	0	0	3,34871 E-07	0	2,15875E -06
Bacteria	*Proteobacteria*	*Betaproteobacteria*	*Burkholderiales*	*Alcaligenaceae*	*Pigmentiphaga*	0	0	0	1,0521E -06	0
Bacteria	*Firmicutes*	*Bacilli*	*Lactobacillales*	*Enterococcaceae*	*Pilibacter*	6,8467E -07	1,49798E-05	1,92029 E-07	0	0
Bacteria	*Planctomycetes*	*Planctomycetia*	*Planctomycetales*	*Planctomycetaceae*	*Pirellula*	0	0	6,35107 E-07	1,56705 E-05	8,50838E -07
Bacteria	*Planctomycetes*	*Planctomycetia*	*Planctomycetales*	*Planctomycetaceae*	*Planctomyces*	0	0	2,38872 E-06	4,70618 E-07	0
Bacteria	*Bacteroidetes*	*Flavobacteriia*	*Flavobacteriales*	*Flavobacteriaceae*	*Planobacterium*	0	0	3,62381 E-06	1,61796 E-06	0

Bacteria	*Firmicutes*	*Bacilli*	*Bacillales*	*Planococcaceae*	*Planococcaceae_ incertae_sedis*	0	0,0001173 28	0,00045 1764	0,00090 1557	6,94775E -05
Bacteria	*Firmicutes*	*Bacilli*	*Bacillales*	*Planococcaceae*	*Planococcus*	0	0	1,40785 E-07	6,49642 E-07	0
Bacteria	*Firmicutes*	*Bacilli*	*Bacillales*	*Planococcaceae*	*Planomicrobium*	0	0	2,5833E -06	6,71297 E-06	8,22341E -06
Bacteria	*Proteobacteria*	*Gammaproteobacteria*	*Enterobacteriales*	*Enterobacteriaceae*	*Pluralibacter*	0	0	2,34959 E-06	0	0
Bacteria	*Proteobacteria*	*Betaproteobacteria*	*Burkholderiales*	*Burkholderiaceae*	*Polynucleobacter*	0	0	0	1,41186 E-06	0
Bacteria	*Firmicutes*	*Bacilli*	*Bacillales*	*Bacillaceae 2*	*Pontibacillus*	0	0	4,35274 E-06	5,43065 E-06	1,75731E -06
Bacteria	*Bacteroidetes*	*Cytophagia*	*Cytophagales*	*Cytophagaceae*	*Pontibacter*	0	0	1,05896 E-06	4,50315 E-07	1,50014E -06
Bacteria	*Proteobacteria*	*Alphaproteobacteria*	*Caulobacterales*	*Hyphomonadaceae*	*Ponticaulis*	0	0	0	3,47728 E-07	0
Bacteria	*Proteobacteria*	*Alphaproteobacteria*	*Sphingomonadales*	*Erythrobacteraceae*	*Porphyrobacter*	1,15613 E-06	0	0	1,29928 E-06	8,635E- 07
Bacteria	*Bacteroidetes*	*Bacteroidia*	*Bacteroidales*	*Porphyromonadaceae*	*Porphyromonas*	0	0	0,00017 2284	0,00024 3151	1,30425E -05
Bacteria	*Bacteroidetes*	*Bacteroidia*	*Bacteroidales*	*Prevotellaceae*	*Prevotella*	0,16021 4152	0,1510314 22	0,09891 2159	0,10080 9421	0,073382 862
Bacteria	*Bacteroidetes*	*Bacteroidetes"_incertae _sedis*	*Prolixibacter*	*Prolixibacter*	*Prolixibacter*	0	1,4956E- 05	4,60037 E-06	1,47076 E-05	0
Bacteria	*Actinobacteria*	*Actinobacteria*	*Actinomycetales*	*Promicromonosporacea e*	*Promicromonosp ora*	0	0	0	3,47728 E-07	0
Bacteria	*Actinobacteria*	*Actinobacteria*	*Actinomycetales*	*Propionibacteriaceae*	*Propionibacteriu m*	2,57095 E-05	2,4028E- 05	6,17395 E-06	3,04602 E-05	8,08753E -05
Bacteria	*Actinobacteria*	*Actinobacteria*	*Actinomycetales*	*Propionibacteriaceae*	*Propionicimonas*	0	0	1,76228 E-05	0	0
Bacteria	*Fusobacteria*	*Fusobacteriia*	*Fusobacteriales*	*Fusobacteriaceae*	*Propionigenium*	0	0	0	1,12532 E-06	0
Bacteria	*Firmicutes*	*Negativicutes*	*Selenomonadales*	*Veillonellaceae*	*Propionispira*	8,35086 E-07	0	0	0	0
Bacteria	*Firmicutes*	*Clostridia*	*Clostridiales*	*Clostridiaceae 1*	*Proteiniclasticu m*	1,15613 E-06	0	7,98767 E-07	1,24024 E-06	0
Bacteria	*Proteobacteria*	*Gammaproteobacteria*	*Enterobacteriales*	*Enterobacteriaceae*	*Proteus*	0	0	3,6043E -07	0	0
Bacteria	*Proteobacteria*	*Deltaproteobacteria*	*Myxococcales*	*Nannocystaceae*	*Pseudenhygromy xa*	0	0	1,27464 E-07	0	0

Bacteria	*Firmicutes*	*Clostridia*	*Clostridiales*	*Lachnospiraceae*	*Pseudobutyrivibrio*	0,002416588	0,002037305	0,003111477	0,006073297	0,001234327
Bacteria	*Proteobacteria*	*Alphaproteobacteria*	*Rhizobiales*	*Brucellaceae*	*Pseudochrobactrum*	0	0	7,60094E-07	0	0
Bacteria	*Firmicutes*	*Clostridia*	*Clostridiales*	*Ruminococcaceae*	*Pseudoflavonifractor*	0,001189221	0,000584021	0,00182829	0,000259868	0,00039378
Bacteria	*Proteobacteria*	*Alphaproteobacteria*	*Rhizobiales*	*Xanthobacteraceae*	*Pseudolabrys*	0	0	6,5546E-07	0	0
Bacteria	*Proteobacteria*	*Gammaproteobacteria*	*Pseudomonadales*	*Pseudomonadaceae*	*Pseudomonas*	3,8817E-06	4,92833E-06	5,04033E-06	2,18081E-05	2,80583E-05
Bacteria	*Actinobacteria*	*Actinobacteria*	*Actinomycetales*	*Pseudonocardiaceae*	*Pseudonocardia*	0	0	1,54097E-06	0	0
Bacteria	*Firmicutes*	*Clostridia*	*Clostridiales*	*Eubacteriaceae*	*Pseudoramibacter*	0	0	3,01381E-06	0	0
Bacteria	*Bacteroidetes*	*Sphingobacteriia*	*Sphingobacteriales*	*Sphingobacteriaceae*	*Pseudosphingobacterium*	6,8467E-07	0,000100891	4,02944E-05	1,24192E-05	0
Bacteria	*Proteobacteria*	*Gammaproteobacteria*	*Xanthomonadales*	*Xanthomonadaceae*	*Pseudoxanthomonas*	0	0	2,49322E-07	0	0
Bacteria	*Firmicutes*	*Bacilli*	*Bacillales*	*Planococcaceae*	*Psychrobacillus*	0	3,80498E-06	9,74464E-06	1,38383E-05	1,71434E-06
Bacteria	*Proteobacteria*	*Gammaproteobacteria*	*Pseudomonadales*	*Moraxellaceae*	*Psychrobacter*	0	0	6,06416E-06	1,29928E-06	5,59621E-05
Bacteria	*Firmicutes*	*Negativicutes*	*Selenomonadales*	*Veillonellaceae*	*Psychrosinus*	0	9,61809E-07	0	0	0
Bacteria	*Proteobacteria*	*Betaproteobacteria*	*Burkholderiales*	*Alcaligenaceae*	*Pusillimonas*	0	0	6,4447E-07	0	0
Bacteria	*Synergistetes*	*Synergistia*	*Synergistales*	*Synergistaceae*	*Pyramidobacter*	6,8467E-07	0,000776705	2,57951E-05	0,000595541	0,001765256
Bacteria	*Actinobacteria*	*Actinobacteria*	*Actinomycetales*	*Kineosporiaceae*	*Quadrisphaera*	0	0	1,3714E-06	0	0
Bacteria	*Proteobacteria*	*Betaproteobacteria*	*Burkholderiales*	*Burkholderiaceae*	*Ralstonia*	0	0	5,24138E-06	0	1,91397E-05
Bacteria	*Proteobacteria*	*Betaproteobacteria*	*Burkholderiales*	*Comamonadaceae*	*Ramlibacter*	0	0	1,56282E-07	0	0
Bacteria	*Proteobacteria*	*Gammaproteobacteria*	*Enterobacteriales*	*Enterobacteriaceae*	*Raoultella*	0	0	9,20301E-07	3,64645E-07	0
Bacteria	*Actinobacteria*	*Actinobacteria*	*Actinomycetales*	*Microbacteriaceae*	*Rathayibacter*	8,35086E-07	0	1,67884E-06	6,66863E-07	1,06904E-05
Bacteria	*Actinobacteria*	*Actinobacteria*	*Actinomycetales*	*Micrococcaceae*	*Renibacterium*	1,86098E-06	0	3,52987E-07	0	1,727E-06

Bacteria	*Proteobacteria*	*Alphaproteobacteria*	*Rhizobiales*	*Rhizobiaceae*	*Rhizobium*	5,31328 E-06	2,38205E-06	2,58125 E-05	6,63572 E-06	9,31254E -06
Bacteria	*Proteobacteria*	*Alphaproteobacteria*	*Alphaproteobacteria _incertae_sedis*	*Rhizomicrobium*	*Rhizomicrobium*	0	0	5,83041 E-07	0	8,38176E -07
Bacteria	*Proteobacteria*	*Alphaproteobacteria*	*Rhodobacterales*	*Rhodobacteraceae*	*Rhodobacter*	0	0	1,80215 E-07	0	0
Bacteria	*Proteobacteria*	*Alphaproteobacteria*	*Rhodospirillales*	*Rhodospirillaceae*	*Rhodocista*	0	0	0	0	7,30695E -07
Bacteria	*Actinobacteria*	*Actinobacteria*	*Actinomycetales*	*Nocardiaceae*	*Rhodococcus*	2,12431 E-06	1,14513E-06	4,40306 E-06	0	5,80542E -06
Bacteria	*Proteobacteria*	*Alphaproteobacteria*	*Rhizobiales*	*Rhodobiaceae*	*Rhodoligotropho s*	0	0	1,56282 E-07	0	0
Bacteria	*Planctomycetes*	*Planctomycetia*	*Planctomycetales*	*Planctomycetaceae*	*Rhodopirellula*	2,31227 E-06	0	6,6027E -07	0	0
Bacteria	*Proteobacteria*	*Alphaproteobacteria*	*Rhizobiales*	*Bradyrhizobiaceae*	*Rhodopseudomo nas*	0	0	1,40785 E-07	0	7,30695E -07
Bacteria	*Bacteroidetes*	*Bacteroidia*	*Bacteroidales*	*Rikenellaceae*	*Rikenella*	4,83626 E-06	0	3,68772 E-07	0	0
Bacteria	*Firmicutes*	*Clostridia*	*Clostridiales*	*Lachnospiraceae*	*Robinsoniella*	0,00012 7883	0,0001700 83	0,00024 9852	0,00020 7358	8,70955E -05
Bacteria	*Firmicutes*	*Clostridia*	*Clostridiales*	*Lachnospiraceae*	*Roseburia*	0,02546 087	0,0111953 29	0,00537 7641	0,03547 226	0,013954 853
Bacteria	*Chloroflexi*	*Chloroflexia*	*Chloroflexales*	*Chloroflexaceae*	*Roseiflexus*	0	0	3,52987 E-07	0	4,3175E-07
Bacteria	*Proteobacteria*	*Alphaproteobacteria*	*Rhodospirillales*	*Acetobacteraceae*	*Roseococcus*	0	0	4,63968 E-07	0	0
Bacteria	*Proteobacteria*	*Alphaproteobacteria*	*Rhodospirillales*	*Acetobacteraceae*	*Roseomonas*	2,24478 E-06	1,22217E-06	1,48982 E-06	4,33095 E-07	0
Bacteria	*Actinobacteria*	*Actinobacteria*	*Actinomycetales*	*Micrococcaceae*	*Rothia*	7,30624 E-06	3,06875E-06	4,64971 E-06	7,81229 E-05	1,38854E -05
Bacteria	*Proteobacteria*	*Alphaproteobacteria*	*Rhodobacterales*	*Rhodobacteraceae*	*Rubellimicrobiu m*	0	0	1,32614 E-06	0	0
Bacteria	*Actinobacteria*	*Actinobacteria*	*Rubrobacterales*	*Rubrobacteraceae*	*Rubrobacter*	1,08774 E-06	1,30262E-06	1,61254 E-06	3,3313E -06	1,84938E -05
Bacteria	*Proteobacteria*	*Gammaproteobacteria*	*Aeromonadales*	*Succinivibrionaceae*	*Ruminobacter*	8,35086 E-07	4,11475E-05	4,9597E -05	1,17028 E-05	0,000983 056
Bacteria	*Firmicutes*	*Clostridia*	*Clostridiales*	*Ruminococcaceae*	*Ruminococcus*	0,03843 0075	0,0232398 13	0,01332 0976	0,01884 9797	0,043513 43
Bacteria	*Firmicutes*	*Clostridia*	*Clostridiales*	*Lachnospiraceae*	*Ruminococcus2*	0,00642 9844	0,0015594 96	0,00159 7831	0,00106 1375	0,000830 592

Bacteria	*Firmicutes*	*Bacilli*	*Bacillales*	*Planococcaceae*	*Rummeliibacillus*	2,21879E-06	0,01323085	0,001110872	1,64701E-06	0
Bacteria	*Candidatus Saccharibacteria*	*Saccharibacteria_genera_incertae_sedis*	*Saccharibacteria_genera_incertae_sedis*	*Saccharibacteria_genera_incertae_sedis*	*Saccharibacteria_genera_incertae_sedis*	1,99122E-06	7,69525E-05	0,00053016	0,002204092	0,000102494
Bacteria	*Firmicutes*	*Clostridia*	*Clostridiales*	*Ruminococcaceae*	*Saccharofermentans*	7,74183E-05	8,2906E-05	0,000278311	0,012447607	7,41021E-06
Bacteria	*Bacteroidetes*	*Sphingobacteriia*	*Sphingobacteriales*	*Rhodothermaceae*	*Salinibacter*	1,15613E-06	0	0	0	0
Bacteria	*Actinobacteria*	*Actinobacteria*	*Actinomycetales*	*Microbacteriaceae*	*Salinibacterium*	0	9,61809E-07	0	0	0
Bacteria	*Bacteroidetes*	*Flavobacteriia*	*Flavobacteriales*	*Flavobacteriaceae*	*Salinimicrobium*	0	0	0	8,66189E-07	1,00009E-06
Bacteria	*Firmicutes*	*Bacilli*	*Bacillales*	*Bacillaceae 2*	*Salirhabdus*	0	0	1,76493E-07	0	0
Bacteria	*Proteobacteria*	*Alphaproteobacteria*	*Sphingomonadales*	*Sphingomonadaceae*	*Sandarakinorhabdus*	0	0	0	4,33095E-07	0
Bacteria	*Actinobacteria*	*Actinobacteria*	*Actinomycetales*	*Sanguibacteraceae*	*Sanguibacter*	0	0	0	9,00631E-07	0
Bacteria	*Firmicutes*	*Clostridia*	*Clostridiales*	*Clostridiaceae 1*	*Sarcina*	9,11237E-06	1,93673E-05	0,000120091	2,10898E-05	5,59336E-05
Bacteria	*Planctomycetes*	*Planctomycetia*	*Planctomycetales*	*Planctomycetaceae*	*Schlesneria*	0	1,22217E-06	8,62017E-07	1,56896E-05	0
Bacteria	*Firmicutes*	*Negativicutes*	*Selenomonadales*	*Veillonellaceae*	*Schwartzia*	0	0,000140537	0,000412857	0,0010394	0
Bacteria	*Firmicutes*	*Clostridia*	*Clostridiales*	*Clostridiales_Incertae Sedis XI*	*Sedimentibacter*	0	0	0,000115639	0	0
Bacteria	*Bacteroidetes*	*Sphingobacteriia*	*Sphingobacteriales*	*Chitinophagaceae*	*Sediminibacterium*	0	0	2,8873E-06	5,79542E-07	2,46169E-05
Bacteria	*Firmicutes*	*Negativicutes*	*Selenomonadales*	*Veillonellaceae*	*Selenomonas*	0	0,000207074	0,001192356	0,001367257	2,5905E-06
Bacteria	*Proteobacteria*	*Gammaproteobacteria*	*Pseudomonadales*	*Pseudomonadaceae*	*Serpens*	0	0	3,4467E-07	0	1,46139E-06
Bacteria	*Firmicutes*	*Erysipelotrichia*	*Erysipelotrichales*	*Erysipelotrichaceae*	*Sharpea*	0	2,60525E-06	0,000853838	2,30087E-06	5,02533E-05
Bacteria	*Proteobacteria*	*Gammaproteobacteria*	*Alteromonadales*	*Shewanellaceae*	*Shewanella*	0	0	0	8,35554E-07	0
Bacteria	*Proteobacteria*	*Betaproteobacteria*	*Rhodocyclales*	*Rhodocyclaceae*	*Shinella*	0	0	3,00521E-07	0	8,635E-07

Bacteria	*Firmicutes*	*Clostridia*	*Clostridiales*	*Lachnospiraceae*	*Shuttleworthia*	1,89674 E-06	2,88793E-06	1,27151 E-05	9,7943E-06	0
Bacteria	*Proteobacteria*	*Betaproteobacteria*	*Neisseriales*	*Neisseriaceae*	*Simonsiella*	0	0	0	0	8,635E-07
Bacteria	*Planctomycetes*	*Planctomycetia*	*Planctomycetales*	*Planctomycetaceae*	*Singulisphaera*	0	0	3,09312 E-07	0	0
Bacteria	*Actinobacteria*	*Actinobacteria*	*Actinomycetales*	*Micrococcaceae*	*Sinomonas*	0	0	1,76493 E-07	2,70477 E-05	0
Bacteria	*Actinobacteria*	*Actinobacteria*	*Coriobacteriales*	*Coriobacteriaceae*	*Slackia*	0	2,31304E-05	0,00010 3075	4,79966 E-05	1,13966E-05
Bacteria	*Firmicutes*	*Bacilli*	*Bacillales*	*Planococcaceae*	*Solibacillus*	0	5,16562E-06	0,00084 9774	0,00101 013	0,001126 393
Bacteria	*Actinobacteria*	*Actinobacteria*	*Solirubrobacterales*	*Solirubrobacteraceae*	*Solirubrobacter*	0	0	2,42555 E-06	1,13443 E-06	0
Bacteria	*Bacteroidetes*	*Sphingobacteriia*	*Sphingobacteriales*	*Sphingobacteriaceae*	*Solitalea*	0	0	0	5,56507 E-06	0
Bacteria	*Firmicutes*	*Erysipelotrichia*	*Erysipelotrichales*	*Erysipelotrichaceae*	*Solobacterium*	0	0	1,44352 E-06	0	0
Bacteria	*Bacteroidetes*	*Flavobacteriia*	*Flavobacteriales*	*Flavobacteriaceae*	*Soonwooa*	1,75639 E-06	9,62643E-07	0	0	0
Bacteria	*Verrucomicrobia*	*Spartobacteria*	*Spartobacteria_genera_incertae_sedis*	*Spartobacteria_genera_incertae_sedis*	*Spartobacteria_genera_incertae_sedis*	2,31227 E-06	0	2,32559 E-06	4,0574E-06	1,32809E-05
Bacteria	*Chloroflexi*	*Thermomicrobia*	*Sphaerobacterales*	*Sphaerobacteraceae*	*Sphaerobacter*	3,4684E-06	0	1,36342 E-05	2,16547 E-07	0
Bacteria	*Spirochaetes*	*Spirochaetia*	*Spirochaetales*	*Spirochaetaceae*	*Sphaerochaeta*	1,36934 E-06	0,0055016 47	0,00116 1582	0,00275 7896	0,010555 532
Bacteria	*Bacteroidetes*	*Sphingobacteriia*	*Sphingobacteriales*	*Sphingobacteriaceae*	*Sphingobacterium*	1,06266 E-06	0	1,35642 E-06	5,80459 E-06	1,727E-06
Bacteria	*Proteobacteria*	*Alphaproteobacteria*	*Sphingomonadales*	*Sphingomonadaceae*	*Sphingobium*	0	0	1,46451 E-06	5,56336 E-07	1,50014E-06
Bacteria	*Proteobacteria*	*Alphaproteobacteria*	*Sphingomonadales*	*Sphingomonadaceae*	*Sphingomonas*	1,15613 E-06	2,66326E-06	3,01419 E-05	0,00010 4352	2,81119E-05
Bacteria	*Proteobacteria*	*Alphaproteobacteria*	*Sphingomonadales*	*Sphingomonadaceae*	*Sphingopyxis*	0	0	1,13042 E-06	0	0
Bacteria	*Proteobacteria*	*Alphaproteobacteria*	*Sphingomonadales*	*Sphingomonadaceae*	*Sphingorhabdus*	0	0	0	2,16547 E-07	0
Bacteria	*Proteobacteria*	*Alphaproteobacteria*	*Sphingomonadales*	*Sphingomonadaceae*	*Sphingosinicella*	0	0	3,12565 E-07	1,15619 E-06	0

Bacteria	*Bacteroidetes*	*Cytophagia*	*Cytophagales*	*Cytophagaceae*	*Spirosoma*	0	0	0	6,49642 E-07	0
Bacteria	*Firmicutes*	*Clostridia*	*Clostridiales*	*Peptostreptococcaceae*	*Sporacetigenium*	2,30344 E-05	4,9932E-06	1,61579 E-05	2,63989 E-05	2,36155E -05
Bacteria	*Actinobacteria*	*Actinobacteria*	*Actinomycetales*	*Sporichthyaceae*	*Sporichthya*	0	0	1,27464 E-07	0	0
Bacteria	*Firmicutes*	*Clostridia*	*Clostridiales*	*Ruminococcaceae*	*Sporobacter*	0,00577 0222	0,0046311 74	0,00623 628	0,00371 7772	0,004484 292
Bacteria	*Firmicutes*	*Clostridia*	*Clostridiales*	*Lachnospiraceae*	*Sporobacterium*	0	0	0	0	4,3175E-07
Bacteria	*SR1*	*SR1_genera_incertae_sedis*	*SR1_genera_incertae_sedis*	*SR1_genera_incertae_sedis*	*SR1_genera_incertae_sedis*	0	0	1,41073 E-05	0,00044 25	0
Bacteria	*Firmicutes*	*Bacilli*	*Bacillales*	*Staphylococcaceae*	*Staphylococcus*	1,95708 E-05	1,56933E-05	8,61121 E-06	0,00015 8163	7,28502E -05
Bacteria	*Proteobacteria*	*Alphaproteobacteria*	*Rhodospirillales*	*Acetobacteraceae*	*Stella*	0	0	2,97068 E-07	0	0
Bacteria	*Proteobacteria*	*Gammaproteobacteria*	*Xanthomonadales*	*Xanthomonadaceae*	*Stenotrophomonas*	0	3,40957E-06	4,1528E -06	1,12805 E-05	8,22341E -07
Bacteria	*Proteobacteria*	*Gammaproteobacteria*	*Xanthomonadales*	*Sinobacteraceae*	*Steroidobacter*	0	9,61809E-07	3,52987 E-07	0	0
Bacteria	*Actinobacteria*	*Actinobacteria*	*Actinomycetales*	*Streptomycetaceae*	*Streptomyces*	0	0	3,52987 E-07	1,22773 E-06	0
Bacteria	*Cyanobacteria/ Chloroplast*	*Chloroplast*	*Chloroplast*	*Chloroplast*	*Streptophyta*	1,77241 E-06	9,1343E-06	0,00132 072	3,62511 E-05	3,20122E -05
Bacteria	*Verrucomicrobia*	*Subdivision3*	*Subdivision3_genera_incertae_sedis*	*Subdivision3_genera_incertae_sedis*	*Subdivision3_genera_incertae_sedis*	0	1,14513E-06	7,81412 E-07	0	0
Bacteria	*Verrucomicrobia*	*Subdivision5*	*Subdivision5_genera_incertae_sedis*	*Subdivision5_genera_incertae_sedis*	*Subdivision5_genera_incertae_sedis*	1,38952 E-06	0,0060027 29	0,00454 1873	0,02035 1255	0,001349 141
Bacteria	*Firmicutes*	*Clostridia*	*Clostridiales*	*Ruminococcaceae*	*Subdoligranulum*	1,40969 E-06	5,47074E-06	1,68778 E-06	6,44992 E-06	1,46139E -06
Bacteria	*Firmicutes*	*Negativicutes*	*Selenomonadales*	*Acidaminococcaceae*	*Succiniclasticum*	0	0	2,22735 E-05	0	0
Bacteria	*Firmicutes*	*Negativicutes*	*Selenomonadales*	*Acidaminococcaceae*	*Succinispira*	0	0	2,07896 E-06	5,23481 E-06	0
Bacteria	*Proteobacteria*	*Gammaproteobacteria*	*Aeromonadales*	*Succinivibrionaceae*	*Succinivibrio*	1,77241 E-06	0,0036422 09	0,00363 348	0,00070 1018	0,002322 991
Bacteria	*Proteobacteria*	*Betaproteobacteria*	*Rhodocyclales*	*Rhodocyclaceae*	*Sulfuritalea*	0	0	0	1,29437 E-06	0

Bacteria	*Proteobacteria*	*Betaproteobacteria*	*Burkholderiales*	*Sutterellaceae*	*Sutterella*	0,001885715	0,001870969	0,000267181	0,000144836	0,00641745
Bacteria	*Proteobacteria*	*Gammaproteobacteria*	*Cardiobacteriales*	*Cardiobacteriaceae*	*Suttonella*	0	0	4,98644E-07	0	1,25726E-06
Bacteria	*Synergistetes*	*Synergistia*	*Synergistales*	*Synergistaceae*	*Synergistes*	0	0	4,98644E-07	0	0
Bacteria	*Firmicutes*	*Clostridia*	*Clostridiales*	*Lachnospiraceae*	*Syntrophococcus*	0	1,36064E-05	4,80963E-06	1,65851E-05	2,12221E-05
Bacteria	*Firmicutes*	*Clostridia*	*Clostridiales*	*Syntrophomonadaceae*	*Syntrophothermus*	0	0	0	1,49066E-05	0
Bacteria	*Bacteroidetes*	*Bacteroidia*	*Bacteroidales*	*Porphyromonadaceae*	*Tannerella*	9,24554E-05	0,001596114	0,00280873	3,12847E-05	2,98878E-05
Bacteria	*Proteobacteria*	*Alphaproteobacteria*	*Rhizobiales*	*Bradyrhizobiaceae*	*Tardiphaga*	0	0	1,54656E-07	0	0
Bacteria	*Proteobacteria*	*Gammaproteobacteria*	*Enterobacteriales*	*Enterobacteriaceae*	*Tatumella*	0	0	6,51909E-06	0	0
Bacteria	*Actinobacteria*	*Actinobacteria*	*Actinomycetales*	*Intrasporangiaceae*	*Terrabacter*	0	0	1,76493E-07	0	0
Bacteria	*Acidobacteria*	*Acidobacteria_Gp1*	*Terriglobus*	*Terriglobus*	*Terriglobus*	0	0	0	1,15619E-06	0
Bacteria	*Actinobacteria*	*Actinobacteria*	*Actinomycetales*	*Propionibacteriaceae*	*Tessaracoccus*	0	0	1,14444E-05	0	0
Bacteria	*Proteobacteria*	*Betaproteobacteria*	*Rhodocyclales*	*Rhodocyclaceae*	*Thauera*	0	0	0	1,02087E-06	0
Bacteria	*Firmicutes*	*Clostridia*	*Clostridiales*	*Clostridiaceae 1*	*Thermobrachium*	0	0	1,76493E-07	0	0
Archaea	*Euryarchaeota*	*Thermoplasmata*	*Thermoplasmatales*	*Thermoplasmatales_incertae_sedis*	*Thermogymnomonas*	0	0,000530091	3,2605E-05	7,81647E-05	0,000202928
Bacteria	*Actinobacteria*	*Thermoleophilia*	*Thermoleophilales*	*Thermoleophilaceae*	*Thermoleophilum*	0	0	1,36693E-06	0	2,15875E-06
Bacteria	*Proteobacteria*	*Gammaproteobacteria*	*Xanthomonadales*	*Xanthomonadaceae*	*Thermomonas*	0	0	1,27464E-07	0	0
Bacteria	*Deinococcus-Thermus*	*Deinococci*	*Thermales*	*Thermaceae*	*Thermus*	0	0	0	2,89771E-07	0
Bacteria	*Proteobacteria*	*Betaproteobacteria*	*Hydrogenophilales*	*Hydrogenophilaceae*	*Thiobacillus*	0	0	0	2,16547E-07	0
Bacteria	*Proteobacteria*	*Betaproteobacteria*	*Burkholderiales*	*Burkholderiales_incertae_sedis*	*Thiobacter*	0	0	1,84386E-07	0	0
Bacteria	*Firmicutes*	*Clostridia*	*Clostridiales*	*Clostridiales_Incertae Sedis XI*	*Tissierella*	0	1,14513E-06	2,3967E-06	0	2,93361E-06

Bacteria	*Proteobacteria*	*Gammaproteobacteria*	*Aeromonadales*	*Aeromonadaceae*	*Tolumonas*	0	0	0	3,47728 E-07	0
Bacteria	*Actinobacteria*	*Actinobacteria*	*Actinomycetales*	*Corynebacterineae_incertae_sedis*	*Tomitella*	0	0	0	2,16547 E-07	0
Bacteria	*Spirochaetes*	*Spirochaetia*	*Spirochaetales*	*Spirochaetaceae*	*Treponema*	6,8467E-06	0,018893157	0,029077193	0,023670822	0,03089014
Bacteria	*Firmicutes*	*Bacilli*	*Lactobacillales*	*Carnobacteriaceae*	*Trichococcus*	0	0	1,0938E-06	0	2,15875E-06
Bacteria	*Proteobacteria*	*Alphaproteobacteria*	*Rhodobacterales*	*Rhodobacteraceae*	*Tropicimonas*	2,31227 E-06	0	0	0	0
Bacteria	*Actinobacteria*	*Actinobacteria*	*Actinomycetales*	*Actinomycetaceae*	*Trueperella*	0	0	1,56282 E-07	8,10705 E-06	0
Bacteria	*Actinobacteria*	*Actinobacteria*	*Actinomycetales*	*Tsukamurellaceae*	*Tsukamurella*	0	0	0	4,02996 E-07	0
Bacteria	*Actinobacteria*	*Actinobacteria*	*Actinomycetales*	*Corynebacteriaceae*	*Turicella*	1,08885 E-05	9,93508E-06	1,58643 E-06	0	6,48123E-06
Bacteria	*Firmicutes*	*Erysipelotrichia*	*Erysipelotrichales*	*Erysipelotrichaceae*	*Turicibacter*	0,00216658	0,000447761	0,000448258	0,000162526	0,005061284
Bacteria	*Proteobacteria*	*Betaproteobacteria*	*Burkholderiales*	*Oxalobacteraceae*	*Undibacterium*	1,82634 E-06	9,61809E-07	3,90107 E-07	1,88959 E-06	5,2508E-06
Bacteria	*Tenericutes*	*Mollicutes*	*Mycoplasmatales*	*Mycoplasmataceae*	*Ureaplasma*	0	0	8,2872E-06	1,57888 E-06	7,24589E-05
Bacteria	*Firmicutes*	*Bacilli*	*Bacillales*	*Planococcaceae*	*Ureibacillus*	0	0	0	0	8,22341E-07
Bacteria	*Firmicutes*	*Bacilli*	*Lactobacillales*	*Enterococcaceae*	*Vagococcus*	0	0	0	0	2,93361E-06
Bacteria	*Proteobacteria*	*Deltaproteobacteria*	*Bdellovibrionales*	*Bdellovibrionaceae*	*Vampirovibrio*	0,011589652	0,005746942	0,004915285	0,005838519	0,019362328
Bacteria	*Proteobacteria*	*Betaproteobacteria*	*Burkholderiales*	*Comamonadaceae*	*Variovorax*	0	1,22217E-06	2,57701 E-06	0	3,23092E-06
Bacteria	*Firmicutes*	*Negativicutes*	*Selenomonadales*	*Veillonellaceae*	*Veillonella*	3,23971 E-06	1,11836E-05	4,03886 E-05	2,15565 E-05	0,000274621
Bacteria	*Verrucomicrobia*	*Verrucomicrobiae*	*Verrucomicrobiales*	*Verrucomicrobiaceae*	*Verrucomicrobium*	0	0	8,06263 E-07	0	0
Bacteria	*Proteobacteria*	*Gammaproteobacteria*	*Vibrionales*	*Vibrionaceae*	*Vibrio*	0	0	0	0	1,50014E-06
Bacteria	*Lentisphaerae*	*Lentisphaeria*	*Victivallales*	*Victivallaceae*	*Victivallis*	4,46225 E-05	0,000271246	7,11297 E-05	5,70807 E-06	9,84705E-05
Bacteria	*Firmicutes*	*Bacilli*	*Bacillales*	*Bacillaceae 2*	*Virgibacillus*	0	0	1,76493 E-07	0	0

Bacteria	*Proteobacteria*	*Betaproteobacteria*	*Neisseriales*	*Neisseriaceae*	*Vitreoscilla*	0	0	5,83041 E-07	0	0
Bacteria	*Bacteroidetes*	*Flavobacteriia*	*Flavobacteriales*	*Cryomorphaceae*	*Wandonia*	0	0	0	4,50315 E-07	0
Bacteria	*Bacteroidetes*	*Flavobacteriia*	*Flavobacteriales*	*Flavobacteriaceae*	*Wautersiella*	1,20231 E-06	1,22217E-06	3,4467E-07	1,2002E-06	1,00009E-06
Bacteria	*Firmicutes*	*Bacilli*	*Lactobacillales*	*Leuconostocaceae*	*Weissella*	0	3,4354E-06	4,81204 E-05	2,16547 E-07	7,56571E-06
Bacteria	*Proteobacteria*	*Epsilonproteobacteria*	*Campylobacterales*	*Helicobacteraceae*	*Wolinella*	0	6,38408E-06	7,42156 E-07	5,95733 E-06	0
Bacteria	*candidate division WPS-1*	*WPS-1_genera_incertae_sedis*	*WPS-1_genera_incertae_sedis*	*WPS-1_genera_incertae_sedis*	*WPS-1_genera_incertae_sedis*	1,08774 E-06	0	3,5999E-06	0	0
Bacteria	*candidate division WPS-2*	*WPS-2_genera_incertae_sedis*	*WPS-2_genera_incertae_sedis*	*WPS-2_genera_incertae_sedis*	*WPS-2_genera_incertae_sedis*	0	0	3,52987 E-07	0	0
Bacteria	*Proteobacteria*	*Gammaproteobacteria*	*Xanthomonadales*	*Xanthomonadaceae*	*Xanthomonas*	0	0	3,00249 E-06	0	1,60753E-05
Bacteria	*Verrucomicrobia*	*Spartobacteria*	*Xiphinematobacter*	*Xiphinematobacter*	*Xiphinematobacter*	0	0	6,18624 E-07	0	0
Bacteria	*Bacteroidetes*	*Bacteroidia*	*Bacteroidales*	*Prevotellaceae*	*Xylanibacter*	9,3919E-06	0,000155304	2,969E-06	1,2504E-05	0
Bacteria	*Actinobacteria*	*Actinobacteria*	*Actinomycetales*	*Micrococcaceae*	*Yaniella*	0	0	1,74217 E-06	0	0
Bacteria	*Proteobacteria*	*Gammaproteobacteria*	*Enterobacteriales*	*Enterobacteriaceae*	*Yersinia*	0	3,08529E-05	0	0	0
Bacteria	*Planctomycetes*	*Planctomycetia*	*Planctomycetales*	*Planctomycetaceae*	*Zavarzinella*	0	0	1,72335 E-07	0	0
Bacteria	*Actinobacteria*	*Actinobacteria*	*Actinomycetales*	*Micrococcaceae*	*Zhihengliuella*	0	0	0	2,16547 E-07	0
Bacteria	*Proteobacteria*	*Betaproteobacteria*	*Rhodocyclales*	*Rhodocyclaceae*	*Zoogloea*	0	0	0	5,78093 E-07	0
Archaea	*Euryarchaeota*	*Methanobacteria*	*Methanobacteriales*	*Methanobacteriaceae*		0	0,001810226	0,005324131	0,000219484	1,30821E-05
Archaea	*Euryarchaeota*	*Thermoplasmata*	*Thermoplasmatales*	*Thermoplasmatales_incertae_sedis*		0	0,000530091	3,2605E-05	7,81647 E-05	0,000202928
Bacteria	*Acidobacteria*	*Acidobacteria_Gp1*	*Candidatus Koribacter*	*Candidatus Koribacter*		0	0	1,92029 E-07	6,2012E-07	0
Bacteria	*Acidobacteria*	*Acidobacteria_Gp1*	*Edaphobacter*	*Edaphobacter*		0	0	4,65595 E-07	0	0

Bacteria	*Acidobacteria*	*Acidobacteria_Gp1*	*Gp1*	*Gp1*		0	0	9,37695 E-07	0	0
Bacteria	*Acidobacteria*	*Acidobacteria_Gp1*	*Granulicella*	*Granulicella*		0	0	0	5,78093 E-07	0
Bacteria	*Acidobacteria*	*Acidobacteria_Gp1*	*Terriglobus*	*Terriglobus*		0	0	0	1,15619 E-06	0
Bacteria	*Acidobacteria*	*Acidobacteria_Gp16*	*Gp16*	*Gp16*		0	0	1,10742 E-06	0	8,635E-07
Bacteria	*Acidobacteria*	*Acidobacteria_Gp2*	*Gp2*	*Gp2*		0	0	8,7086E-07	0	1,55733E-05
Bacteria	*Acidobacteria*	*Acidobacteria_Gp3*	*Gp3*	*Gp3*		0	0	9,698E-07	5,56336 E-07	0
Bacteria	*Acidobacteria*	*Acidobacteria_Gp4*	*Blastocatella*	*Blastocatella*		0	0	8,82467 E-07	6,49642 E-07	0
Bacteria	*Acidobacteria*	*Acidobacteria_Gp4*	*Gp4*	*Gp4*		6,51926 E-06	0	2,17315 E-06	8,46107 E-07	0
Bacteria	*Acidobacteria*	*Acidobacteria_Gp6*	*Gp6*	*Gp6*		2,31227 E-06	0	5,80644 E-06	0	0
Bacteria	*Acidobacteria*	*Acidobacteria_Gp7*	*Gp7*	*Gp7*		0	0	7,53505 E-07	4,50315 E-07	0
Bacteria	*Actinobacteria*	*Actinobacteria*	*Acidimicrobiales*	*Acidimicrobiaceae*		5,78067 E-06	0	5,82098 E-07	3,64645 E-07	1,46139E-06
Bacteria	*Actinobacteria*	*Actinobacteria*	*Acidimicrobiales*	*Acidimicrobineae"_incertae_sedis*		0	0	6,02309 E-07	0	0
Bacteria	*Actinobacteria*	*Actinobacteria*	*Acidimicrobiales*	*Iamiaceae*		0	0	9,60051 E-07	0	0
Bacteria	*Actinobacteria*	*Actinobacteria*	*Actinomycetales*	*Acidothermaceae*		0	0	1,76493 E-07	0	0
Bacteria	*Actinobacteria*	*Actinobacteria*	*Actinomycetales*	*Actinomycetaceae*		4,05959 E-06	3,32911E-06	3,68537 E-05	2,73528 E-05	4,25586E-06
Bacteria	*Actinobacteria*	*Actinobacteria*	*Actinomycetales*	*Beutenbergiaceae*		0	0	1,92724 E-07	0	0
Bacteria	*Actinobacteria*	*Actinobacteria*	*Actinomycetales*	*Bogoriellaceae*		0	0	6,67018 E-07	0	0
Bacteria	*Actinobacteria*	*Actinobacteria*	*Actinomycetales*	*Brevibacteriaceae*		3,42656 E-06	0	1,96173 E-06	6,2012E-07	5,98119E-06
Bacteria	*Actinobacteria*	*Actinobacteria*	*Actinomycetales*	*Cellulomonadaceae*		0	0	1,10121 E-06	1,01429 E-06	0
Bacteria	*Actinobacteria*	*Actinobacteria*	*Actinomycetales*	*Corynebacteriaceae*		2,95128 E-05	3,62898E-05	6,62453 E-05	1,00516 E-05	0,000190008

Bacteria	*Actinobacteria*	*Actinobacteria*	*Actinomycetales*	*Corynebacterineae_incertae_sedis*		0	0	0	2,16547E-07	0
Bacteria	*Actinobacteria*	*Actinobacteria*	*Actinomycetales*	*Demequinaceae*		0	0	7,20861E-07	0	0
Bacteria	*Actinobacteria*	*Actinobacteria*	*Actinomycetales*	*Dermabacteraceae*		0	0	1,63303E-05	2,16547E-07	2,67644E-06
Bacteria	*Actinobacteria*	*Actinobacteria*	*Actinomycetales*	*Dermacoccaceae*		0	0	2,09912E-06	0	0
Bacteria	*Actinobacteria*	*Actinobacteria*	*Actinomycetales*	*Dietziaceae*		1,07312E-05	9,61809E-07	3,2231E-05	1,23742E-06	2,07444E-05
Bacteria	*Actinobacteria*	*Actinobacteria*	*Actinomycetales*	*Geodermatophilaceae*		0	0	1,4846E-06	1,45679E-06	2,50818E-06
Bacteria	*Actinobacteria*	*Actinobacteria*	*Actinomycetales*	*Intrasporangiaceae*		3,4684E-06	9,62643E-07	0,000166751	3,25913E-06	6,82568E-06
Bacteria	*Actinobacteria*	*Actinobacteria*	*Actinomycetales*	*Kineosporiaceae*		0	0	2,10978E-06	1,17646E-06	0
Bacteria	*Actinobacteria*	*Actinobacteria*	*Actinomycetales*	*Microbacteriaceae*		2,66142E-06	4,80441E-06	2,51594E-05	5,86442E-06	7,26272E-05
Bacteria	*Actinobacteria*	*Actinobacteria*	*Actinomycetales*	*Micrococcaceae*		1,41485E-05	5,21073E-06	4,75692E-05	0,004297798	5,09956E-05
Bacteria	*Actinobacteria*	*Actinobacteria*	*Actinomycetales*	*Micromonosporaceae*		0	0	1,1211E-06	0	5,75639E-06
Bacteria	*Actinobacteria*	*Actinobacteria*	*Actinomycetales*	*Mycobacteriaceae*		0	0	3,02927E-06	6,09384E-06	1,64468E-06
Bacteria	*Actinobacteria*	*Actinobacteria*	*Actinomycetales*	*Nakamurellaceae*		0	0	4,67221E-07	4,02996E-07	4,3175E-07
Bacteria	*Actinobacteria*	*Actinobacteria*	*Actinomycetales*	*Nocardiaceae*		2,12431E-06	1,14513E-06	8,37373E-06	0	3,29873E-05
Bacteria	*Actinobacteria*	*Actinobacteria*	*Actinomycetales*	*Nocardioidaceae*		5,78067E-06	0	1,16763E-05	5,75268E-06	0
Bacteria	*Actinobacteria*	*Actinobacteria*	*Actinomycetales*	*Promicromonosporaceae*		0	0	1,13133E-06	5,64275E-07	5,0482E-06
Bacteria	*Actinobacteria*	*Actinobacteria*	*Actinomycetales*	*Propionibacteriaceae*		2,57095E-05	2,4028E-05	3,75492E-05	3,12955E-05	8,64484E-05
Bacteria	*Actinobacteria*	*Actinobacteria*	*Actinomycetales*	*Pseudonocardiaceae*		0	0	2,11914E-06	0	7,30695E-07
Bacteria	*Actinobacteria*	*Actinobacteria*	*Actinomycetales*	*Sanguibacteraceae*		0	0	0	9,00631E-07	0
Bacteria	*Actinobacteria*	*Actinobacteria*	*Actinomycetales*	*Sporichthyaceae*		0	0	1,27464E-07	0	0

Bacteria	*Actinobacteria*	*Actinobacteria*	*Actinomycetales*	*Streptomycetaceae*		0	0	3,52987 E-07	1,22773 E-06	0
Bacteria	*Actinobacteria*	*Actinobacteria*	*Actinomycetales*	*Tsukamurellaceae*		0	0	0	4,02996 E-07	0
Bacteria	*Actinobacteria*	*Actinobacteria*	*Bifidobacteriales*	*Bifidobacteriaceae*		0	2,201E-05	0,00136 4881	0	0,000224 56
Bacteria	*Actinobacteria*	*Actinobacteria*	*Coriobacteriales*	*Coriobacteriaceae*		0,00075 6844	0,0011467 68	0,00576 8236	0,00192 916	0,001054 867
Bacteria	*Actinobacteria*	*Actinobacteria*	*Euzebyales*	*Euzebyaceae*		0	0	3,52987 E-07	0	0
Bacteria	*Actinobacteria*	*Actinobacteria*	*Gaiellales*	*Gaiellaceae*		0	0	2,32654 E-06	1,74562 E-06	2,02595E -06
Bacteria	*Actinobacteria*	*Actinobacteria*	*Rubrobacterales*	*Rubrobacteraceae*		1,08774 E-06	1,30262E-06	1,61254 E-06	3,3313E -06	1,84938E -05
Bacteria	*Actinobacteria*	*Actinobacteria*	*Solirubrobacterales*	*Conexibacteraceae*		1,15613 E-06	0	8,44179 E-07	0	0
Bacteria	*Actinobacteria*	*Actinobacteria*	*Solirubrobacterales*	*Patulibacteraceae*		0	0	0	6,2012E -07	0
Bacteria	*Actinobacteria*	*Actinobacteria*	*Solirubrobacterales*	*Solirubrobacteraceae*		0	0	2,42555 E-06	1,13443 E-06	0
Bacteria	*Actinobacteria*	*Thermoleophilia*	*Thermoleophilales*	*Thermoleophilaceae*		0	0	1,36693 E-06	0	2,15875E -06
Bacteria	*Armatimonadetes*	*Armatimonadetes_gp4*	*Armatimonadetes_gp4*	*Armatimonadetes_gp4*		0	0	3,85448 E-07	0	0
Bacteria	*Armatimonadetes*	*Armatimonadetes_gp5*	*Armatimonadetes_gp5*	*Armatimonadetes_gp5*		0	0	4,56803 E-07	2,16547 E-07	0
Bacteria	*Armatimonadetes*	*Armatimonadia*	*Armatimonadales*	*Armatimonadaceae*		0	0	5,2948E -07	0	0
Bacteria	*Armatimonadetes*	*Chthonomonadetes*	*Chthonomonadales*	*Chthonomonadaceae*		0	0	2,8157E -07	0	0
Bacteria	*BRC1*	*BRC1_genera_incertae_sedis*	*BRC1_genera_incertae_sedis*	*BRC1_genera_incertae_sedis*		0	0	0	2,16547 E-07	0
Bacteria	*Bacteroidetes*	*Bacteroidetes"_incertae_sedis*	*Marinifilum*	*Marinifilum*		0	0	0	0	4,3175E-07
Bacteria	*Bacteroidetes*	*Bacteroidetes"_incertae_sedis*	*Ohtaekwangia*	*Ohtaekwangia*		2,31227 E-06	0	1,05189 E-06	0	0
Bacteria	*Bacteroidetes*	*Bacteroidetes"_incertae_sedis*	*Prolixibacter*	*Prolixibacter*		0	1,4956E-05	4,60037 E-06	1,47076 E-05	0
Bacteria	*Bacteroidetes*	*Bacteroidia*	*Bacteroidales*	*Bacteroidaceae*		0,05184 428	0,0120371 78	0,02026 617	0,00197 1161	0,028345 395

Bacteria	*Bacteroidetes*	*Bacteroidia*	*Bacteroidales*	*Bacteroidales"_incertae_sedis*		0	0	1,12067E-06	6,37546E-05	0
Bacteria	*Bacteroidetes*	*Bacteroidia*	*Bacteroidales*	*Marinilabiliaceae*		0	0	1,16074E-06	3,28161E-06	1,9543E-05
Bacteria	*Bacteroidetes*	*Bacteroidia*	*Bacteroidales*	*Porphyromonadaceae*		0,034561785	0,0506325	0,076216376	0,026358342	0,048062276
Bacteria	*Bacteroidetes*	*Bacteroidia*	*Bacteroidales*	*Prevotellaceae*		0,176025408	0,24043013	0,142395485	0,146148025	0,096734508
Bacteria	*Bacteroidetes*	*Bacteroidia*	*Bacteroidales*	*Rikenellaceae*		0,027483265	0,000501952	0,000177965	6,29729E-06	0,000884586
Bacteria	*Bacteroidetes*	*Cytophagia*	*Cytophagales*	*Cytophagaceae*		2,52547E-06	5,40539E-06	8,3456E-06	4,53189E-05	1,91922E-06
Bacteria	*Bacteroidetes*	*Cytophagia*	*Cytophagales*	*Flammeovirgaceae*		0	0	1,27464E-07	4,70618E-07	0
Bacteria	*Bacteroidetes*	*Flavobacteriia*	*Flavobacteriales*	*Cryomorphaceae*		0	6,07143E-06	1,20756E-06	1,51085E-05	0
Bacteria	*Bacteroidetes*	*Flavobacteriia*	*Flavobacteriales*	*Flavobacteriaceae*		1,38899E-05	0,009118713	0,011517735	0,016462586	0,007175793
Bacteria	*Bacteroidetes*	*Sphingobacteriia*	*Sphingobacteriales*	*Chitinophagaceae*		1,15613E-06	1,14513E-06	1,23792E-05	0,004729916	3,18238E-05
Bacteria	*Bacteroidetes*	*Sphingobacteriia*	*Sphingobacteriales*	*Rhodothermaceae*		1,15613E-06	0	0	0	0
Bacteria	*Bacteroidetes*	*Sphingobacteriia*	*Sphingobacteriales*	*Saprospiraceae*		0	0	0	9,70778E-07	0
Bacteria	*Bacteroidetes*	*Sphingobacteriia*	*Sphingobacteriales*	*Sphingobacteriaceae*		1,08697E-05	0,015231129	0,004527715	0,001547794	1,6074E-05
Bacteria	*Candidatus Saccharibacteria*	*Saccharibacteria_genera_incertae_sedis*	*Saccharibacteria_genera_incertae_sedis*	*Saccharibacteria_genera_incertae_sedis*		1,99122E-06	7,69525E-05	0,00053016	0,002204092	0,000102494
Bacteria	*Chlamydiae*	*Chlamydiia*	*Chlamydiales*	*Chlamydiaceae*		0	0,00069837	7,64786E-07	0,000768975	0
Bacteria	*Chlamydiae*	*Chlamydiia*	*Chlamydiales*	*Parachlamydiaceae*		0	0	2,22173E-06	0	1,25726E-06
Bacteria	*Chloroflexi*	*Anaerolineae*	*Anaerolineales*	*Anaerolineaceae*		0	0	1,76493E-07	0,000191085	4,38417E-06
Bacteria	*Chloroflexi*	*Caldilineae*	*Caldilineales*	*Caldilineaceae*		5,78067E-06	0	0	0	0
Bacteria	*Chloroflexi*	*Chloroflexia*	*Chloroflexales*	*Chloroflexaceae*		0	0	3,52987E-07	0	4,3175E-07

Bacteria	*Chloroflexi*	*Thermomicrobia*	*Sphaerobacterales*	*Sphaerobacteraceae*		3,4684E-06	0	1,36342E-05	2,16547E-07	0
Bacteria	*Cyanobacteria/ Chloroplast*	*Chloroplast*	*Chloroplast*	*Chloroplast*		3,94789E-06	1,03565E-05	0,001322938	3,93451E-05	3,27429E-05
Bacteria	*Cyanobacteria/ Chloroplast*	*Cyanobacteria*	*Family II*	*Family II*		0	0	0	8,20773E-07	0
Bacteria	*Cyanobacteria/ Chloroplast*	*Cyanobacteria*	*Family XIII*	*Family XIII*		0	0	1,56282E-07	4,33095E-07	0
Bacteria	*Deferribacteres*	*Deferribacteres*	*Deferribacterales*	*Deferribacteraceae*		0	5,59831E-05	5,09107E-05	0,000582846	0
Bacteria	*Deinococcus-Thermus*	*Deinococci*	*Deinococcales*	*Deinococcaceae*		3,4684E-06	0	1,80215E-07	4,70618E-07	8,635E-07
Bacteria	*Deinococcus-Thermus*	*Deinococci*	*Thermales*	*Thermaceae*		0	0	0	2,89771E-07	2,15875E-06
Bacteria	*Elusimicrobia*	*Elusimicrobia*	*Elusimicrobiales*	*Elusimicrobiaceae*		0,006730554	0,001870505	0,000328789	0	0,008924486
Bacteria	*Fibrobacteres*	*Fibrobacteria*	*Fibrobacterales*	*Fibrobacteraceae*		0	0,000746676	0,00226435	0,00128398	0,000893402
Bacteria	*Firmicutes*	*Bacilli*	*Bacillales*	*Bacillaceae 1*		0	0	0,01416457	0,002479004	0,003630439
Bacteria	*Firmicutes*	*Bacilli*	*Bacillales*	*Bacillaceae 2*		0	4,48899E-06	0,0001166	3,57568E-05	1,38718E-05
Bacteria	*Firmicutes*	*Bacilli*	*Bacillales*	*Bacillales_Incertae Sedis XI*		2,1233E-06	0	7,89213E-07	0	8,10378E-06
Bacteria	*Firmicutes*	*Bacilli*	*Bacillales*	*Bacillales_Incertae Sedis XII*		0	0	5,82297E-06	0	0
Bacteria	*Firmicutes*	*Bacilli*	*Bacillales*	*Listeriaceae*		0	0	0	0	2,15875E-06
Bacteria	*Firmicutes*	*Bacilli*	*Bacillales*	*Paenibacillaceae 1*		0	0	1,42281E-06	2,29949E-05	0
Bacteria	*Firmicutes*	*Bacilli*	*Bacillales*	*Paenibacillaceae 2*		0	1,36064E-06	2,49322E-07	0	0
Bacteria	*Firmicutes*	*Bacilli*	*Bacillales*	*Planococcaceae*		1,00073E-05	0,013556235	0,005071204	0,004071065	0,001670146
Bacteria	*Firmicutes*	*Bacilli*	*Bacillales*	*Staphylococcaceae*		1,95708E-05	1,56933E-05	1,01267E-05	0,000158633	7,82365E-05
Bacteria	*Firmicutes*	*Bacilli*	*Lactobacillales*	*Aerococcaceae*		1,79259E-06	2,50577E-06	5,86989E-05	3,77181E-05	3,76656E-05
Bacteria	*Firmicutes*	*Bacilli*	*Lactobacillales*	*Carnobacteriaceae*		6,85991E-06	6,13168E-06	9,37774E-06	4,39996E-06	4,51041E-05

Bacteria	*Firmicutes*	*Bacilli*	*Lactobacillales*	*Enterococcaceae*		5,73739 E-06	1,62824E-05	2,50554 E-05	1,49512 E-06	0,000558 781
Bacteria	*Firmicutes*	*Bacilli*	*Lactobacillales*	*Lactobacillaceae*		5,5825E -05	0,0049134 09	0,01219 4856	0,00062 9258	0,000454 776
Bacteria	*Firmicutes*	*Bacilli*	*Lactobacillales*	*Leuconostocaceae*		0	7,26281E-06	5,20423 E-05	2,16547 E-07	7,56571E -06
Bacteria	*Firmicutes*	*Clostridia*	*Clostridiales*	*Clostridiaceae 1*		0,00148 0926	0,0019029 7	0,00580 0648	0,00385 8588	0,001638 696
Bacteria	*Firmicutes*	*Clostridia*	*Clostridiales*	*Clostridiaceae 2*		0	1,14513E-06	1,27784 E-06	1,25444 E-06	0
Bacteria	*Firmicutes*	*Clostridia*	*Clostridiales*	*Clostridiaceae 3*		0	0	1,54656 E-07	0	0
Bacteria	*Firmicutes*	*Clostridia*	*Clostridiales*	*Clostridiaceae 4*		8,35086 E-07	2,99596E-06	2,77498 E-06	0	1,89314E -06
Bacteria	*Firmicutes*	*Clostridia*	*Clostridiales*	*Clostridiales_Incertae Sedis XI*		0,00012 1624	7,35651E-05	0,00016 4506	1,9857E -05	3,82361E -05
Bacteria	*Firmicutes*	*Clostridia*	*Clostridiales*	*Clostridiales_Incertae Sedis XII*		0,00014 3982	0,0003027 9	0,00140 5746	8,43298 E-05	0,000313 427
Bacteria	*Firmicutes*	*Clostridia*	*Clostridiales*	*Clostridiales_Incertae Sedis XIII*		0,00696 5627	0,004626	0,00772 0518	0,00735 4206	0,001293 406
Bacteria	*Firmicutes*	*Clostridia*	*Clostridiales*	*Defluviitaleaceae*		0,00075 7012	0,0001402 38	0,00013 9525	0,00091 8847	0,000154 871
Bacteria	*Firmicutes*	*Clostridia*	*Clostridiales*	*Eubacteriaceae*		0,00284 0856	0,0041131 37	0,00764 0591	0,00408 2753	0,002767 016
Bacteria	*Firmicutes*	*Clostridia*	*Clostridiales*	*Gracilibacteraceae*		0,00066 336	0,0005466 49	0,00052 4963	9,83864 E-05	0,001660 955
Bacteria	*Firmicutes*	*Clostridia*	*Clostridiales*	*Incertae Sedis XI*		0	0	3,98414 E-06	5,8752E -05	0
Bacteria	*Firmicutes*	*Clostridia*	*Clostridiales*	*Lachnospiraceae*		0,14097 9019	0,1413934 23	0,16733 6771	0,19924 934	0,165506 083
Bacteria	*Firmicutes*	*Clostridia*	*Clostridiales*	*Natranaerovirga*		0	0	1,22257 E-06	3,23593 E-07	8,635E-07
Bacteria	*Firmicutes*	*Clostridia*	*Clostridiales*	*Peptococcaceae 1*		0,00022 6247	0,0001304 71	0,00053 7594	0,00039 0801	0,001385 708
Bacteria	*Firmicutes*	*Clostridia*	*Clostridiales*	*Peptostreptococcaceae*		0,00335 2002	0,0024977 11	0,00261 2007	0,00574 793	0,018231 012
Bacteria	*Firmicutes*	*Clostridia*	*Clostridiales*	*Ruminococcaceae*		0,36890 5606	0,2143051 39	0,20411 6357	0,13881 0581	0,217065 546
Bacteria	*Firmicutes*	*Clostridia*	*Clostridiales*	*Syntrophomonadaceae*		1,35932 E-05	4,54903E-06	3,14114 E-05	4,34577 E-05	1,92161E -05

Bacteria	*Firmicutes*	*Clostridia*	*Thermoanaerobacterales*	*Thermoanaerobacteraceae*		0	0	1,56282E-07	1,23561E-06	0
Bacteria	*Firmicutes*	*Erysipelotrichia*	*Erysipelotrichales*	*Erysipelotrichaceae*		0,012482446	0,005930106	0,011187702	0,021082713	0,01591162
Bacteria	*Firmicutes*	*Negativicutes*	*Selenomonadales*	*Acidaminococcaceae*		0,010739222	0,032791287	0,017857787	0,05070666	0,004934776
Bacteria	*Firmicutes*	*Negativicutes*	*Selenomonadales*	*Veillonellaceae*		0,004248334	0,010184416	0,01369963	0,00788023	0,002743927
Bacteria	*Fusobacteria*	*Fusobacteriia*	*Fusobacteriales*	*Fusobacteriaceae*		7,04847E-07	4,88868E-06	0,000509656	6,70056E-05	5,46591E-05
Bacteria	*Fusobacteria*	*Fusobacteriia*	*Fusobacteriales*	*Leptotrichiaceae*		1,06266E-06	2,10778E-06	3,90107E-07	0	7,30695E-07
Bacteria	*Gemmatimonadetes*	*Gemmatimonadetes*	*Gemmatimonadales*	*Gemmatimonadaceae*		0	0	6,20688E-06	2,12386E-06	8,22341E-07
Bacteria	*Lentisphaerae*	*Lentisphaeria*	*Victivallales*	*Victivallaceae*		4,46225E-05	0,000271246	7,11297E-05	5,70807E-06	9,84705E-05
Bacteria	*Lentisphaerae*	*Oligosphaeria*	*Oligosphaerales*	*Oligosphaeraceae*		0	5,69725E-06	2,11151E-06	2,77339E-06	0
Bacteria	*Nitrospirae*	*Nitrospira*	*Nitrospirales*	*Nitrospiraceae*		0	0	2,1459E-06	0	0
Bacteria	*Parcubacteria*	*Parcubacteria_genera_incertae_sedis*	*Parcubacteria_genera_incertae_sedis*	*Parcubacteria_genera_incertae_sedis*		0	0	6,24915E-07	4,02996E-07	5,61184E-06
Bacteria	*Planctomycetes*	*Planctomycetia*	*Planctomycetales*	*Planctomycetaceae*		4,62453E-06	0,007323585	0,01076704	0,009285287	0,001085587
Bacteria	*Proteobacteria*	*Alphaproteobacteria*	*Alphaproteobacteria_incertae_sedis*	*Rhizomicrobium*		0	0	5,83041E-07	0	8,38176E-07
Bacteria	*Proteobacteria*	*Alphaproteobacteria*	*Caulobacterales*	*Caulobacteraceae*		2,56583E-06	0	2,15041E-06	1,88706E-06	1,78427E-05
Bacteria	*Proteobacteria*	*Alphaproteobacteria*	*Caulobacterales*	*Hyphomonadaceae*		0	0	0	3,47728E-07	0
Bacteria	*Proteobacteria*	*Alphaproteobacteria*	*Kiloniellales*	*Kiloniellaceae*		0	0	0	2,20656E-05	0
Bacteria	*Proteobacteria*	*Alphaproteobacteria*	*Rhizobiales*	*Aurantimonadaceae*		0	0	8,83564E-06	8,67864E-07	0
Bacteria	*Proteobacteria*	*Alphaproteobacteria*	*Rhizobiales*	*Beijerinckiaceae*		0	0	3,15115E-06	7,26588E-07	4,11171E-06
Bacteria	*Proteobacteria*	*Alphaproteobacteria*	*Rhizobiales*	*Bradyrhizobiaceae*		1,06165E-06	1,36064E-06	4,90262E-06	1,51478E-05	1,2638E-05
Bacteria	*Proteobacteria*	*Alphaproteobacteria*	*Rhizobiales*	*Brucellaceae*		8,20734E-06	1,07943E-06	1,15968E-06	4,03688E-06	6,30366E-06

Bacteria	*Proteobacteria*	*Alphaproteobacteria*	*Rhizobiales*	*Hyphomicrobiaceae*		0	0	1,0588E-05	1,69077E-06	1,36355E-06
Bacteria	*Proteobacteria*	*Alphaproteobacteria*	*Rhizobiales*	*Methylobacteriaceae*		0	0	2,11412E-05	2,76533E-06	5,48613E-06
Bacteria	*Proteobacteria*	*Alphaproteobacteria*	*Rhizobiales*	*Methylocystaceae*		0	0	4,59573E-07	5,56336E-07	0
Bacteria	*Proteobacteria*	*Alphaproteobacteria*	*Rhizobiales*	*Phyllobacteriaceae*		0	0	1,99838E-06	2,41776E-06	9,50151E-06
Bacteria	*Proteobacteria*	*Alphaproteobacteria*	*Rhizobiales*	*Rhizobiaceae*		5,31328E-06	3,68468E-06	2,58125E-05	6,63572E-06	9,31254E-06
Bacteria	*Proteobacteria*	*Alphaproteobacteria*	*Rhizobiales*	*Rhodobiaceae*		0	0	1,56282E-07	0	0
Bacteria	*Proteobacteria*	*Alphaproteobacteria*	*Rhizobiales*	*Xanthobacteraceae*		0	0	1,01589E-06	4,02996E-07	0
Bacteria	*Proteobacteria*	*Alphaproteobacteria*	*Rhodobacterales*	*Rhodobacteraceae*		6,61382E-06	1,30262E-06	1,61849E-05	8,8341E-07	1,28221E-05
Bacteria	*Proteobacteria*	*Alphaproteobacteria*	*Rhodospirillales*	*Acetobacteraceae*		2,24478E-06	1,22217E-06	1,70627E-05	4,33095E-07	1,79097E-05
Bacteria	*Proteobacteria*	*Alphaproteobacteria*	*Rhodospirillales*	*Rhodospirillaceae*		0,000519404	0,001545014	0,000332412	9,38372E-05	0,018000358
Bacteria	*Proteobacteria*	*Alphaproteobacteria*	*Sphingomonadales*	*Erythrobacteraceae*		2,31227E-06	0	8,27261E-07	1,29928E-06	8,635E-07
Bacteria	*Proteobacteria*	*Alphaproteobacteria*	*Sphingomonadales*	*Sphingomonadaceae*		6,10172E-06	2,66326E-06	3,88224E-05	0,000109177	3,43332E-05
Bacteria	*Proteobacteria*	*Betaproteobacteria*	*Burkholderiales*	*Alcaligenaceae*		1,15613E-06	0	9,06892E-06	0,000182285	3,09644E-05
Bacteria	*Proteobacteria*	*Betaproteobacteria*	*Burkholderiales*	*Burkholderiaceae*		0	1,14513E-06	9,69571E-06	1,70163E-06	2,06399E-05
Bacteria	*Proteobacteria*	*Betaproteobacteria*	*Burkholderiales*	*Burkholderiales_incertae_sedis*		0	3,07042E-06	1,47771E-06	6,7568E-06	9,51866E-06
Bacteria	*Proteobacteria*	*Betaproteobacteria*	*Burkholderiales*	*Comamonadaceae*		6,01126E-06	4,74594E-06	1,78073E-05	6,39482E-06	4,94657E-05
Bacteria	*Proteobacteria*	*Betaproteobacteria*	*Burkholderiales*	*Oxalobacteraceae*		4,61894E-06	0,001342538	0,001270138	0,001200895	1,12958E-05
Bacteria	*Proteobacteria*	*Betaproteobacteria*	*Burkholderiales*	*Sutterellaceae*		0,004290141	0,002097645	0,000297244	0,000167927	0,008069616
Bacteria	*Proteobacteria*	*Betaproteobacteria*	*Hydrogenophilales*	*Hydrogenophilaceae*		0	0	0	2,16547E-07	0
Bacteria	*Proteobacteria*	*Betaproteobacteria*	*Methylophilales*	*Methylophilaceae*		0	0	7,74293E-06	1,15619E-06	0

Bacteria	*Proteobacteria*	*Betaproteobacteria*	*Neisseriales*	*Neisseriaceae*		2,80998E-06	3,32911E-06	0,000182356	7,1367E-06	5,1524E-05
Bacteria	*Proteobacteria*	*Betaproteobacteria*	*Rhodocyclales*	*Rhodocyclaceae*		0	0	4,56803E-07	2,89334E-06	2,53985E-06
Bacteria	*Proteobacteria*	*Deltaproteobacteria*	*Bdellovibrionales*	*Bacteriovoracaceae*		0	0	1,66935E-07	0	4,11171E-06
Bacteria	*Proteobacteria*	*Deltaproteobacteria*	*Bdellovibrionales*	*Bdellovibrionaceae*		0,011590715	0,005746942	0,004916348	0,005839097	0,019362328
Bacteria	*Proteobacteria*	*Deltaproteobacteria*	*Desulfobacterales*	*Desulfobacteraceae*		0	4,39721E-06	2,10239E-05	0,000691621	4,19088E-07
Bacteria	*Proteobacteria*	*Deltaproteobacteria*	*Desulfobacterales*	*Desulfobulbaceae*		0	0	0	6,95456E-07	0
Bacteria	*Proteobacteria*	*Deltaproteobacteria*	*Desulfovibrionales*	*Desulfohalobiaceae*		0	1,07943E-06	4,97911E-06	0	0
Bacteria	*Proteobacteria*	*Deltaproteobacteria*	*Desulfovibrionales*	*Desulfomicrobiaceae*		0	2,52479E-06	0	2,38682E-06	0
Bacteria	*Proteobacteria*	*Deltaproteobacteria*	*Desulfovibrionales*	*Desulfovibrionaceae*		0,000833182	0,002978155	0,003277759	0,001665183	0,002000912
Bacteria	*Proteobacteria*	*Deltaproteobacteria*	*Desulfuromonadales*	*Geobacteraceae*		0	0	1,15357E-06	3,23593E-07	0
Bacteria	*Proteobacteria*	*Deltaproteobacteria*	*Myxococcales*	*Cystobacteraceae*		0	0	5,2948E-07	0,000133073	0
Bacteria	*Proteobacteria*	*Deltaproteobacteria*	*Myxococcales*	*Kofleriaceae*		0	0	3,12565E-07	0	0
Bacteria	*Proteobacteria*	*Deltaproteobacteria*	*Myxococcales*	*Myxococcaceae*		0	0	0	5,78093E-07	1,00009E-06
Bacteria	*Proteobacteria*	*Deltaproteobacteria*	*Myxococcales*	*Nannocystaceae*		0	0	1,27464E-07	0	0
Bacteria	*Proteobacteria*	*Deltaproteobacteria*	*Myxococcales*	*Phaselicystidaceae*		0	0	1,29646E-06	0	0
Bacteria	*Proteobacteria*	*Deltaproteobacteria*	*Myxococcales*	*Polyangiaceae*		0	0	8,51877E-07	0	0
Bacteria	*Proteobacteria*	*Epsilonproteobacteria*	*Campylobacterales*	*Campylobacteraceae*		1,41169E-05	0,003820261	0,000545093	0,008122387	5,87048E-05
Bacteria	*Proteobacteria*	*Epsilonproteobacteria*	*Campylobacterales*	*Helicobacteraceae*		8,35086E-07	0,000316326	0,000206677	0,000694006	0
Bacteria	*Proteobacteria*	*Epsilonproteobacteria*	*Campylobacterales*	*Hydrogenimonaceae*		0	0	1,80215E-07	0	0
Bacteria	*Proteobacteria*	*Gammaproteobacteria*	*Aeromonadales*	*Aeromonadaceae*		7,38594E-07	0	1,80215E-07	4,53045E-06	0

Bacteria	*Proteobacteria*	*Gammaproteobacteria*	*Aeromonadales*	*Succinivibrionaceae*		2,6075E-06	0,003687507	0,003683549	0,001393893	0,003306048
Bacteria	*Proteobacteria*	*Gammaproteobacteria*	*Alteromonadales*	*Alteromonadaceae*		0	0	1,80215E-07	6,2012E-07	0
Bacteria	*Proteobacteria*	*Gammaproteobacteria*	*Alteromonadales*	*Shewanellaceae*		0	0	0	8,35554E-07	0
Bacteria	*Proteobacteria*	*Gammaproteobacteria*	*Candidatus Carsonella*	*Candidatus Carsonella*		9,43072E-06	2,60525E-06	1,48739E-06	0	0
Bacteria	*Proteobacteria*	*Gammaproteobacteria*	*Cardiobacteriales*	*Cardiobacteriaceae*		0	0	6,54926E-07	0	1,25726E-06
Bacteria	*Proteobacteria*	*Gammaproteobacteria*	*Chromatiales*	*Ectothiorhodospiraceae*		0	0	2,49322E-07	0	0
Bacteria	*Proteobacteria*	*Gammaproteobacteria*	*Enterobacteriales*	*Enterobacteriaceae*		0,000905198	0,00050358	0,028481597	0,000918235	0,002410403
Bacteria	*Proteobacteria*	*Gammaproteobacteria*	*Legionellales*	*Coxiellaceae*		0	0	1,26676E-06	0,000110221	2,55251E-06
Bacteria	*Proteobacteria*	*Gammaproteobacteria*	*Legionellales*	*Legionellaceae*		0	1,30262E-06	4,31831E-07	0	0
Bacteria	*Proteobacteria*	*Gammaproteobacteria*	*Orbales*	*Orbaceae*		0	0	1,91197E-06	0	0
Bacteria	*Proteobacteria*	*Gammaproteobacteria*	*Pasteurellales*	*Pasteurellaceae*		6,62708E-05	8,77891E-06	0,00024449	3,02386E-05	0,001064204
Bacteria	*Proteobacteria*	*Gammaproteobacteria*	*Pseudomonadales*	*Pseudomonadaceae*		3,8817E-06	4,92833E-06	5,73755E-06	2,28908E-05	2,95197E-05
Bacteria	*Proteobacteria*	*Gammaproteobacteria*	*Vibrionales*	*Vibrionaceae*		0	0	0	0	1,50014E-06
Bacteria	*Proteobacteria*	*Gammaproteobacteria*	*Xanthomonadales*	*Sinobacteraceae*		0	9,61809E-07	3,52987E-07	0	0
Bacteria	*Proteobacteria*	*Gammaproteobacteria*	*Xanthomonadales*	*Xanthomonadaceae*		2,1233E-06	3,40957E-06	1,12561E-05	1,40088E-05	3,22392E-05
Bacteria	*SR1*	*SR1_genera_incertae_sedis*	*SR1_genera_incertae_sedis*	*SR1_genera_incertae_sedis*		0	0	1,41073E-05	0,0004425	0
Bacteria	*Spirochaetes*	*Spirochaetia*	*Spirochaetales*	*Spirochaetaceae*		8,21604E-06	0,024454153	0,030379064	0,026480042	0,041468533
Bacteria	*Synergistetes*	*Synergistia*	*Synergistales*	*Synergistaceae*		6,8467E-07	0,000992028	4,27386E-05	0,000601401	0,001769282
Bacteria	*Tenericutes*	*Mollicutes*	*Acholeplasmatales*	*Acholeplasmataceae*		0,000522859	6,47658E-06	6,06724E-05	0,000126821	0,00804251
Bacteria	*Tenericutes*	*Mollicutes*	*Anaeroplasmatales*	*Anaeroplasmataceae*		0,002411235	0,001194081	0,00091422	0,002240633	0,01191534

Bacteria	*Tenericutes*	*Mollicutes*	*Haloplasmatales*	*Haloplasmataceae*		0	0	0	0	1,25726E-06
Bacteria	*Tenericutes*	*Mollicutes*	*Mycoplasmatales*	*Mycoplasmataceae*		7,38594E-07	0	6,63663E-05	7,30395E-05	0,000460551
Bacteria	*Verrucomicrobia*	*Opitutae*	*Opitutales*	*Opitutaceae*		1,15613E-06	0	5,78172E-07	0	1,00009E-06
Bacteria	*Verrucomicrobia*	*Opitutae*	*Puniceicoccales*	*Puniceicoccaceae*		6,67592E-05	0,000618422	0,000357573	2,21597E-05	0,000389671
Bacteria	*Verrucomicrobia*	*Spartobacteria*	*Spartobacteria_genera_incertae_sedis*	*Spartobacteria_genera_incertae_sedis*		2,31227E-06	0	2,32559E-06	4,0574E-06	1,32809E-05
Bacteria	*Verrucomicrobia*	*Spartobacteria*	*Xiphinematobacter*	*Xiphinematobacter*		0	0	6,18624E-07	0	0
Bacteria	*Verrucomicrobia*	*Subdivision3*	*Subdivision3_genera_incertae_sedis*	*Subdivision3_genera_incertae_sedis*		0	1,14513E-06	7,81412E-07	0	0
Bacteria	*Verrucomicrobia*	*Subdivision5*	*Subdivision5_genera_incertae_sedis*	*Subdivision5_genera_incertae_sedis*		1,38952E-06	0,006002729	0,004541873	0,020351255	0,001349141
Bacteria	*Verrucomicrobia*	*Verrucomicrobiae*	*Verrucomicrobiales*	*Verrucomicrobiaceae*		0,001670242	0,002463152	0,010415019	1,51586E-05	9,9488E-06
Bacteria	*candidate division WPS-1*	*WPS-1_genera_incertae_sedis*	*WPS-1_genera_incertae_sedis*	*WPS-1_genera_incertae_sedis*		1,08774E-06	0	3,5999E-06	0	0
Bacteria	*candidate division WPS-2*	*WPS-2_genera_incertae_sedis*	*WPS-2_genera_incertae_sedis*	*WPS-2_genera_incertae_sedis*		0	0	3,52987E-07	0	0
Archaea	*Euryarchaeota*	*Methanobacteria*	*Methanobacteriales*			0	0,001810226	0,005324131	0,000219484	1,30821E-05
Archaea	*Euryarchaeota*	*Thermoplasmata*	*Thermoplasmatales*			0	0,000532381	3,2605E-05	9,04343E-05	0,000323092
Bacteria	*Acidobacteria*	*Acidobacteria_Gp1*	*Candidatus Koribacter*			0	0	1,92029E-07	6,2012E-07	0
Bacteria	*Acidobacteria*	*Acidobacteria_Gp1*	*Edaphobacter*			0	0	4,65595E-07	0	0
Bacteria	*Acidobacteria*	*Acidobacteria_Gp1*	*Gp1*			0	0	9,37695E-07	0	0
Bacteria	*Acidobacteria*	*Acidobacteria_Gp1*	*Granulicella*			0	0	0	5,78093E-07	0
Bacteria	*Acidobacteria*	*Acidobacteria_Gp1*	*Terriglobus*			0	0	0	1,15619E-06	0
Bacteria	*Acidobacteria*	*Acidobacteria_Gp16*	*Gp16*			0	0	1,10742E-06	0	8,635E-07

Bacteria	*Acidobacteria*	*Acidobacteria_Gp2*	*Gp2*			0	0	8,7086E-07	0	1,55733E-05
Bacteria	*Acidobacteria*	*Acidobacteria_Gp3*	*Gp3*			0	0	9,698E-07	5,56336E-07	0
Bacteria	*Acidobacteria*	*Acidobacteria_Gp4*	*Blastocatella*			0	0	8,82467E-07	6,49642E-07	0
Bacteria	*Acidobacteria*	*Acidobacteria_Gp4*	*Gp4*			6,51926E-06	0	2,17315E-06	8,46107E-07	0
Bacteria	*Acidobacteria*	*Acidobacteria_Gp6*	*Gp6*			2,31227E-06	0	5,80644E-06	0	0
Bacteria	*Acidobacteria*	*Acidobacteria_Gp7*	*Gp7*			0	0	7,53505E-07	4,50315E-07	0
Bacteria	*Actinobacteria*	*Actinobacteria*	*Acidimicrobiales*			6,84232E-06	0	2,68973E-06	5,81193E-07	2,92278E-06
Bacteria	*Actinobacteria*	*Actinobacteria*	*Actinomycetales*			0,000104593	8,40193E-05	0,000481324	0,004471199	0,000535434
Bacteria	*Actinobacteria*	*Actinobacteria*	*Bifidobacteriales*			0	2,201E-05	0,001364881	0	0,00022456
Bacteria	*Actinobacteria*	*Actinobacteria*	*Coriobacteriales*			0,000756844	0,001146768	0,005768236	0,00192916	0,001054867
Bacteria	*Actinobacteria*	*Actinobacteria*	*Euzebyales*			0	0	3,52987E-07	0	0
Bacteria	*Actinobacteria*	*Actinobacteria*	*Gaiellales*			0	0	2,32654E-06	1,74562E-06	2,02595E-06
Bacteria	*Actinobacteria*	*Actinobacteria*	*Rubrobacterales*			1,08774E-06	1,30262E-06	1,61254E-06	3,3313E-06	1,84938E-05
Bacteria	*Actinobacteria*	*Actinobacteria*	*Solirubrobacterales*			1,15613E-06	0	3,44622E-06	2,68182E-06	0
Bacteria	*Actinobacteria*	*Thermoleophilia*	*Thermoleophilales*			0	0	1,36693E-06	0	2,15875E-06
Bacteria	*Armatimonadetes*	*Armatimonadetes_gp4*	*Armatimonadetes_gp4*			0	0	3,85448E-07	0	0
Bacteria	*Armatimonadetes*	*Armatimonadetes_gp5*	*Armatimonadetes_gp5*			0	0	4,56803E-07	2,16547E-07	0
Bacteria	*Armatimonadetes*	*Armatimonadia*	*Armatimonadales*			0	0	5,2948E-07	0	0
Bacteria	*Armatimonadetes*	*Chthonomonadetes*	*Chthonomonadales*			0	0	2,8157E-07	0	0
Bacteria	*BRC1*	*BRC1_genera_incertae_sedis*	*BRC1_genera_incertae_sedis*			0	0	0	2,16547E-07	0

Bacteria	*Bacteroidetes*	*Bacteroidetes"_incertae_sedis*	*Marinifilum*			0	0	0	0	4,3175E-07
Bacteria	*Bacteroidetes*	*Bacteroidetes"_incertae_sedis*	*Ohtaekwangia*			2,31227E-06	0	1,05189E-06	0	0
Bacteria	*Bacteroidetes*	*Bacteroidetes"_incertae_sedis*	*Prolixibacter*			0	1,4956E-05	4,60037E-06	1,47076E-05	0
Bacteria	*Bacteroidetes*	*Bacteroidia*	*Bacteroidales*			0,290746039	0,33988478	0,280910854	0,288149096	0,229883752
Bacteria	*Bacteroidetes*	*Cytophagia*	*Cytophagales*			2,52547E-06	1,26195E-05	1,19124E-05	5,98303E-05	2,41927E-06
Bacteria	*Bacteroidetes*	*Flavobacteriia*	*Flavobacteriales*			1,38899E-05	0,00920804	0,011573752	0,017043473	0,007180883
Bacteria	*Bacteroidetes*	*Sphingobacteriia*	*Sphingobacteriales*			1,38667E-05	0,015260894	0,004552561	0,026804329	5,0705E-05
Bacteria	*Candidatus Saccharibacteria*	*Saccharibacteria_genera_incertae_sedis*	*Saccharibacteria_genera_incertae_sedis*			1,99122E-06	7,69525E-05	0,00053016	0,002204092	0,000102494
Bacteria	*Chlamydiae*	*Chlamydiia*	*Chlamydiales*			0	0,00069837	2,98652E-06	0,000768975	1,25726E-06
Bacteria	*Chloroflexi*	*Anaerolineae*	*Anaerolineales*			0	0	1,76493E-07	0,000191085	4,38417E-06
Bacteria	*Chloroflexi*	*Caldilineae*	*Caldilineales*			5,78067E-06	0	0	0	0
Bacteria	*Chloroflexi*	*Chloroflexia*	*Chloroflexales*			0	0	3,52987E-07	0	4,3175E-07
Bacteria	*Chloroflexi*	*Thermomicrobia*	*Sphaerobacterales*			3,4684E-06	0	1,36342E-05	2,16547E-07	0
Bacteria	*Cyanobacteria/ Chloroplast*	*Chloroplast*	*Chloroplast*			3,94789E-06	1,03565E-05	0,001322938	3,93451E-05	3,27429E-05
Bacteria	*Cyanobacteria/ Chloroplast*	*Cyanobacteria*	*Family II*			0	0	0	8,20773E-07	0
Bacteria	*Cyanobacteria/ Chloroplast*	*Cyanobacteria*	*Family XIII*			0	0	1,56282E-07	4,33095E-07	0
Bacteria	*Deferribacteres*	*Deferribacteres*	*Deferribacterales*			0	5,59831E-05	5,09107E-05	0,000582846	0
Bacteria	*Deinococcus-Thermus*	*Deinococci*	*Deinococcales*			3,4684E-06	0	1,80215E-07	4,70618E-07	8,635E-07
Bacteria	*Deinococcus-Thermus*	*Deinococci*	*Thermales*			0	0	0	2,89771E-07	2,15875E-06

Bacteria	*Elusimicrobia*	*Elusimicrobia*	*Elusimicrobiales*			0,006730554	0,001870505	0,000328789	0	0,008924486
Bacteria	*Fibrobacteres*	*Fibrobacteria*	*Fibrobacterales*			0	0,000746676	0,00226435	0,00128398	0,000893402
Bacteria	*Firmicutes*	*Bacilli*	*Bacillales*			3,17014E-05	0,013580499	0,019719812	0,006967404	0,005469463
Bacteria	*Firmicutes*	*Bacilli*	*Lactobacillales*			0,000918568	0,008939034	0,012976297	0,00103845	0,002042462
Bacteria	*Firmicutes*	*Clostridia*	*Clostridiales*			0,629183636	0,424302199	0,47020051	0,420828574	0,543203645
Bacteria	*Firmicutes*	*Clostridia*	*Thermoanaerobacterales*			0	0	1,56282E-07	1,23561E-06	0
Bacteria	*Firmicutes*	*Erysipelotrichia*	*Erysipelotrichales*			0,012482446	0,005930106	0,011187702	0,021082713	0,01591162
Bacteria	*Firmicutes*	*Negativicutes*	*Selenomonadales*			0,015014763	0,043081154	0,031627344	0,058734824	0,007685603
Bacteria	*Fusobacteria*	*Fusobacteriia*	*Fusobacteriales*			1,7675E-06	6,99645E-06	0,000510223	6,70056E-05	5,53898E-05
Bacteria	*Gemmatimonadetes*	*Gemmatimonadetes*	*Gemmatimonadales*			0	0	6,20688E-06	2,12386E-06	8,22341E-07
Bacteria	*Lentisphaerae*	*Lentisphaeria*	*Victivallales*			4,46225E-05	0,000271246	7,11297E-05	5,70807E-06	9,84705E-05
Bacteria	*Lentisphaerae*	*Oligosphaeria*	*Oligosphaerales*			0	5,69725E-06	2,11151E-06	2,77339E-06	0
Bacteria	*Nitrospirae*	*Nitrospira*	*Nitrospirales*			0	0	2,1459E-06	0	0
Bacteria	*Parcubacteria*	*Parcubacteria_genera_incertae_sedis*	*Parcubacteria_genera_incertae_sedis*			0	0	6,24915E-07	4,02996E-07	5,61184E-06
Bacteria	*Planctomycetes*	*Planctomycetia*	*Planctomycetales*			4,62453E-06	0,007323585	0,01076704	0,009285287	0,001085587
Bacteria	*Proteobacteria*	*Alphaproteobacteria*	*Alphaproteobacteria_incertae_sedis*			0	0	5,83041E-07	0	8,38176E-07
Bacteria	*Proteobacteria*	*Alphaproteobacteria*	*Caulobacterales*			2,56583E-06	0	2,15041E-06	2,23479E-06	1,78427E-05
Bacteria	*Proteobacteria*	*Alphaproteobacteria*	*Kiloniellales*			0	0	0	2,20656E-05	0
Bacteria	*Proteobacteria*	*Alphaproteobacteria*	*Rhizobiales*			1,45823E-05	7,30408E-06	0,000103458	3,68598E-05	5,01784E-05
Bacteria	*Proteobacteria*	*Alphaproteobacteria*	*Rhodobacterales*			6,61382E-06	1,30262E-06	1,61849E-05	8,8341E-07	1,28221E-05

Bacteria	*Proteobacteria*	*Alphaproteobacteria*	*Rhodospirillales*			0,001412539	0,002116782	0,000387044	0,000390195	0,01887948
Bacteria	*Proteobacteria*	*Alphaproteobacteria*	*Rickettsiales*			0	0	1,56282E-07	0	0
Bacteria	*Proteobacteria*	*Alphaproteobacteria*	*Sphingomonadales*			8,41399E-06	2,66326E-06	4,00027E-05	0,000111417	3,73888E-05
Bacteria	*Proteobacteria*	*Betaproteobacteria*	*Burkholderiales*			0,004319317	0,003685703	0,001616321	0,001801367	0,008208058
Bacteria	*Proteobacteria*	*Betaproteobacteria*	*Hydrogenophilales*			0	0	0	2,16547E-07	0
Bacteria	*Proteobacteria*	*Betaproteobacteria*	*Methylophilales*			0	0	7,74293E-06	1,15619E-06	0
Bacteria	*Proteobacteria*	*Betaproteobacteria*	*Neisseriales*			2,80998E-06	3,32911E-06	0,000182356	7,1367E-06	5,1524E-05
Bacteria	*Proteobacteria*	*Betaproteobacteria*	*Rhodocyclales*			0	0	4,56803E-07	2,89334E-06	2,53985E-06
Bacteria	*Proteobacteria*	*Deltaproteobacteria*	*Bdellovibrionales*			0,011590715	0,005746942	0,00491684	0,005840964	0,019366439
Bacteria	*Proteobacteria*	*Deltaproteobacteria*	*Desulfobacterales*			0	4,39721E-06	2,10239E-05	0,000692971	4,19088E-07
Bacteria	*Proteobacteria*	*Deltaproteobacteria*	*Desulfovibrionales*			0,000836348	0,002990103	0,003294146	0,001672549	0,002000912
Bacteria	*Proteobacteria*	*Deltaproteobacteria*	*Desulfuromonadales*			0	0	1,15357E-06	3,23593E-07	0
Bacteria	*Proteobacteria*	*Deltaproteobacteria*	*Myxococcales*			0	0	4,05651E-06	0,000134721	1,00009E-06
Bacteria	*Proteobacteria*	*Epsilonproteobacteria*	*Campylobacterales*			1,4952E-05	0,004146872	0,000752416	0,008829858	5,87048E-05
Bacteria	*Proteobacteria*	*Gammaproteobacteria*	*Aeromonadales*			3,34609E-06	0,003687507	0,003683729	0,001398424	0,003306048
Bacteria	*Proteobacteria*	*Gammaproteobacteria*	*Alteromonadales*			0	0	1,80215E-07	1,45567E-06	0
Bacteria	*Proteobacteria*	*Gammaproteobacteria*	*Candidatus Carsonella*			9,43072E-06	2,60525E-06	1,48739E-06	0	0
Bacteria	*Proteobacteria*	*Gammaproteobacteria*	*Cardiobacteriales*			0	0	6,54926E-07	0	1,25726E-06
Bacteria	*Proteobacteria*	*Gammaproteobacteria*	*Chromatiales*			0	0	2,49322E-07	0	0
Bacteria	*Proteobacteria*	*Gammaproteobacteria*	*Enterobacteriales*			0,000905198	0,00050358	0,028481597	0,000918235	0,002410403

Bacteria	*Proteobacteria*	*Gammaproteobacteria*	*Legionellales*			0	1,30262E-06	1,69859E-06	0,000110221	2,55251E-06
Bacteria	*Proteobacteria*	*Gammaproteobacteria*	*Oceanospirillales*			0	0	0	0	3,02225E-06
Bacteria	*Proteobacteria*	*Gammaproteobacteria*	*Orbales*			0	0	1,91197E-06	0	0
Bacteria	*Proteobacteria*	*Gammaproteobacteria*	*Pasteurellales*			6,62708E-05	8,77891E-06	0,00024449	3,02386E-05	0,001064204
Bacteria	*Proteobacteria*	*Gammaproteobacteria*	*Vibrionales*			0	0	0	0	1,50014E-06
Bacteria	*Proteobacteria*	*Gammaproteobacteria*	*Xanthomonadales*			2,1233E-06	4,37137E-06	1,16091E-05	1,40088E-05	3,22392E-05
Bacteria	*SR1*	*SR1_genera_incertae_sedis*	*SR1_genera_incertae_sedis*			0	0	1,41073E-05	0,0004425	0
Bacteria	*Spirochaetes*	*Spirochaetia*	*Spirochaetales*			8,21604E-06	0,024746571	0,030418188	0,026490124	0,041474055
Bacteria	*Synergistetes*	*Synergistia*	*Synergistales*			6,8467E-07	0,000992028	4,27386E-05	0,000601401	0,001769282
Bacteria	*Tenericutes*	*Mollicutes*	*Acholeplasmatales*			0,000522859	6,47658E-06	6,06724E-05	0,000126821	0,00804251
Bacteria	*Tenericutes*	*Mollicutes*	*Anaeroplasmatales*			0,002411235	0,001194081	0,00091422	0,002240633	0,01191534
Bacteria	*Tenericutes*	*Mollicutes*	*Haloplasmatales*			0	0	0	0	1,25726E-06
Bacteria	*Tenericutes*	*Mollicutes*	*Mycoplasmatales*			7,38594E-07	0	6,63663E-05	7,30395E-05	0,000460551
Bacteria	*Verrucomicrobia*	*Opitutae*	*Opitutales*			1,15613E-06	0	5,78172E-07	0	1,00009E-06
Bacteria	*Verrucomicrobia*	*Opitutae*	*Puniceicoccales*			6,67592E-05	0,000618422	0,000357573	2,21597E-05	0,000389671
Bacteria	*Verrucomicrobia*	*Spartobacteria*	*Spartobacteria_genera_incertae_sedis*			2,31227E-06	0	2,32559E-06	4,0574E-06	1,32809E-05
Bacteria	*Verrucomicrobia*	*Spartobacteria*	*Xiphinematobacter*			0	0	6,18624E-07	0	0
Bacteria	*Verrucomicrobia*	*Subdivision3*	*Subdivision3_genera_incertae_sedis*			0	1,14513E-06	7,81412E-07	0	0
Bacteria	*Verrucomicrobia*	*Subdivision5*	*Subdivision5_genera_incertae_sedis*			1,38952E-06	0,006002729	0,004541873	0,020351255	0,001349141
Bacteria	*Verrucomicrobia*	*Verrucomicrobiae*	*Verrucomicrobiales*			0,001670242	0,002463152	0,010415019	1,51586E-05	9,9488E-06

Bacteria	*candidate division WPS-1*	*WPS-1_genera_incertae_sedis*	*WPS-1_genera_incertae_sedis*			1,08774E-06	0	3,5999E-06	0	0
Bacteria	*candidate division WPS-2*	*WPS-2_genera_incertae_sedis*	*WPS-2_genera_incertae_sedis*			0	0	3,52987E-07	0	0
Archaea	*Crenarchaeota*	*Thermoprotei*				0	0	0	4,70618E-07	0
Archaea	*Euryarchaeota*	*Methanobacteria*				0	0,001810226	0,005324131	0,000219484	1,30821E-05
Archaea	*Euryarchaeota*	*Thermoplasmata*				0	0,000532381	3,2605E-05	9,04343E-05	0,000323092
Bacteria	*Acidobacteria*	*Acidobacteria_Gp1*				0	0	1,59532E-06	2,3544E-06	0
Bacteria	*Acidobacteria*	*Acidobacteria_Gp16*				0	0	1,10742E-06	0	8,635E-07
Bacteria	*Acidobacteria*	*Acidobacteria_Gp2*				0	0	8,7086E-07	0	1,55733E-05
Bacteria	*Acidobacteria*	*Acidobacteria_Gp3*				0	0	9,698E-07	5,56336E-07	0
Bacteria	*Acidobacteria*	*Acidobacteria_Gp4*				6,51926E-06	0	3,05562E-06	1,49575E-06	0
Bacteria	*Acidobacteria*	*Acidobacteria_Gp6*				2,31227E-06	0	5,80644E-06	0	0
Bacteria	*Acidobacteria*	*Acidobacteria_Gp7*				0	0	7,53505E-07	4,50315E-07	0
Bacteria	*Actinobacteria*	*Actinobacteria*				0,000870523	0,0012541	0,007633133	0,006409349	0,001838735
Bacteria	*Actinobacteria*	*Thermoleophilia*				0	0	1,36693E-06	0	2,15875E-06
Bacteria	*Armatimonadetes*	*Armatimonadetes_gp4*				0	0	3,85448E-07	0	0
Bacteria	*Armatimonadetes*	*Armatimonadetes_gp5*				0	0	4,56803E-07	2,16547E-07	0
Bacteria	*Armatimonadetes*	*Armatimonadia*				0	0	5,2948E-07	0	0
Bacteria	*Armatimonadetes*	*Chthonomonadetes*				0	0	2,8157E-07	0	0
Bacteria	*BRC1*	*BRC1_genera_incertae_sedis*				0	0	0	2,16547E-07	0

Bacteria	*Bacteroidetes*	*Bacteroidetes"_incertae_sedis*				2,31227E-06	1,4956E-05	5,65226E-06	1,47076E-05	4,3175E-07
Bacteria	*Bacteroidetes*	*Bacteroidia*				0,290746039	0,33988478	0,280910854	0,288149096	0,229883752
Bacteria	*Bacteroidetes*	*Cytophagia*				2,52547E-06	1,26195E-05	1,19124E-05	5,98303E-05	2,41927E-06
Bacteria	*Bacteroidetes*	*Flavobacteriia*				1,38899E-05	0,00920804	0,011573752	0,017043473	0,007180883
Bacteria	*Bacteroidetes*	*Sphingobacteriia*				1,38667E-05	0,015260894	0,004552561	0,026804329	5,0705E-05
Bacteria	*Candidatus Saccharibacteria*	*Saccharibacteria_genera_incertae_sedis*				1,99122E-06	7,69525E-05	0,00053016	0,002204092	0,000102494
Bacteria	*Chlamydiae*	*Chlamydiia*				0	0,00069837	2,98652E-06	0,000768975	1,25726E-06
Bacteria	*Chloroflexi*	*Anaerolineae*				0	0	1,76493E-07	0,000191085	4,38417E-06
Bacteria	*Chloroflexi*	*Caldilineae*				5,78067E-06	0	0	0	0
Bacteria	*Chloroflexi*	*Chloroflexia*				0	0	3,52987E-07	0	4,3175E-07
Bacteria	*Chloroflexi*	*Thermomicrobia*				3,4684E-06	0	1,36342E-05	2,16547E-07	0
Bacteria	*Cyanobacteria/ Chloroplast*	*Chloroplast*				3,94789E-06	1,03565E-05	0,001322938	3,93451E-05	3,27429E-05
Bacteria	*Cyanobacteria/ Chloroplast*	*Cyanobacteria*				0	0	1,56282E-07	1,25387E-06	0
Bacteria	*Deferribacteres*	*Deferribacteres*				0	5,59831E-05	5,09107E-05	0,000582846	0
Bacteria	*Deinococcus-Thermus*	*Deinococci*				3,4684E-06	0	1,80215E-07	7,60389E-07	3,02225E-06
Bacteria	*Elusimicrobia*	*Elusimicrobia*				0,006730554	0,001870505	0,000328789	0	0,008924486
Bacteria	*Fibrobacteres*	*Fibrobacteria*				0	0,000746676	0,00226435	0,00128398	0,000893402
Bacteria	*Firmicutes*	*Bacilli*				0,000950269	0,022535178	0,032754053	0,008013824	0,007518291
Bacteria	*Firmicutes*	*Clostridia*				0,630208217	0,426567052	0,471265358	0,427587309	0,545333861

Bacteria	*Firmicutes*	*Erysipelotrichia*				0,012482446	0,005930106	0,011187702	0,021082713	0,01591162
Bacteria	*Firmicutes*	*Negativicutes*				0,015014763	0,043081154	0,031627344	0,058734824	0,007685603
Bacteria	*Fusobacteria*	*Fusobacteriia*				1,7675E-06	6,99645E-06	0,000510223	6,70056E-05	5,53898E-05
Bacteria	*Gemmatimonadetes*	*Gemmatimonadetes*				0	0	6,20688E-06	2,12386E-06	8,22341E-07
Bacteria	*Lentisphaerae*	*Lentisphaeria*				4,46225E-05	0,000272391	7,13217E-05	5,70807E-06	9,84705E-05
Bacteria	*Lentisphaerae*	*Oligosphaeria*				0	5,69725E-06	2,11151E-06	2,77339E-06	0
Bacteria	*Nitrospirae*	*Nitrospira*				0	0	2,1459E-06	0	0
Bacteria	*Parcubacteria*	*Parcubacteria_genera_incertae_sedis*				0	0	6,24915E-07	4,02996E-07	5,61184E-06
Bacteria	*Planctomycetes*	*Planctomycetia*				4,62453E-06	0,007323585	0,01076704	0,009285983	0,001085587
Bacteria	*Proteobacteria*	*Alphaproteobacteria*				0,001454969	0,003871628	0,00140705	0,000617295	0,029638273
Bacteria	*Proteobacteria*	*Betaproteobacteria*				0,004334266	0,004373673	0,001883433	0,001823811	0,008281712
Bacteria	*Proteobacteria*	*Deltaproteobacteria*				0,017184354	0,014597826	0,011548267	0,012092951	0,024241712
Bacteria	*Proteobacteria*	*Epsilonproteobacteria*				1,4952E-05	0,004147834	0,000752878	0,008832211	5,87048E-05
Bacteria	*Proteobacteria*	*Gammaproteobacteria*				0,001019516	0,004261377	0,032850724	0,0083072	0,007059411
Bacteria	*SR1*	*SR1_genera_incertae_sedis*				0	0	1,41073E-05	0,0004425	0
Bacteria	*Spirochaetes*	*Spirochaetia*				8,21604E-06	0,024746571	0,030418188	0,026490124	0,041474055
Bacteria	*Synergistetes*	*Synergistia*				6,8467E-07	0,000992028	4,27386E-05	0,000601401	0,001769282
Bacteria	*Tenericutes*	*Mollicutes*				0,003734463	0,001290828	0,00118606	0,002679968	0,022091917
Bacteria	*Verrucomicrobia*	*Opitutae*				6,79153E-05	0,000618422	0,000360838	2,26304E-05	0,000566955
Bacteria	*Verrucomicrobia*	*Spartobacteria*				2,31227E-06	0	3,56284E-06	4,0574E-06	1,32809E-05

Bacteria	*Verrucomicrobia*	*Subdivision3*				0	1,14513E-06	7,81412E-07	0	0
Bacteria	*Verrucomicrobia*	*Subdivision5*				1,38952E-06	0,006002729	0,004541873	0,020351255	0,001349141
Bacteria	*Verrucomicrobia*	*Verrucomicrobiae*				0,001670242	0,002463152	0,010415019	1,51586E-05	9,9488E-06
Bacteria	*candidate division WPS-1*	*WPS-1_genera_incertae_sedis*				1,08774E-06	0	3,5999E-06	0	0
Bacteria	*candidate division WPS-2*	*WPS-2_genera_incertae_sedis*				0	0	3,52987E-07	0	0
Archaea	*Crenarchaeota*					0	0	0	4,70618E-07	0
Archaea	*Euryarchaeota*					2,54565E-06	0,002394293	0,005357289	0,000365841	0,000337428
Bacteria	*Acidobacteria*					8,83153E-06	0	1,4159E-05	4,8568E-06	1,64368E-05
Bacteria	*Actinobacteria*					0,000870523	0,0012541	0,00763532	0,006409565	0,001840894
Bacteria	*Armatimonadetes*					0	0	1,6533E-06	2,16547E-07	0
Bacteria	*BRC1*					0	0	0	2,16547E-07	0
Bacteria	*Bacteroidetes*					0,291083445	0,401004064	0,311448285	0,368506551	0,239693449
Bacteria	*Candidatus Saccharibacteria*					1,99122E-06	7,69525E-05	0,00053016	0,002204092	0,000102494
Bacteria	*Chlamydiae*					0	0,0006983 7	2,98652E-06	0,000768975	1,25726E-06
Bacteria	*Chloroflexi*					1,15613E-05	0	1,43402E-05	0,000191922	7,74953E-06
Bacteria	*Cyanobacteria/Chloroplast*					3,94789E-06	1,03565E-05	0,001323095	4,05989E-05	3,27429E-05
Bacteria	*Deferribacteres*					0	5,59831E-05	5,09107E-05	0,000582846	0
Bacteria	*Deinococcus-Thermus*					3,4684E-06	0	1,80215E-07	7,60389E-07	3,02225E-06

Bacteria	*Elusimicrobia*					0,006730554	0,001870505	0,000328789	0	0,008924486
Bacteria	*Fibrobacteres*					0	0,000746676	0,00226435	0,00128398	0,000893402
Bacteria	*Firmicutes*					0,668907321	0,505983391	0,556126004	0,518970629	0,599803485
Bacteria	*Fusobacteria*					1,7675E-06	6,99645E-06	0,000510223	6,70056E-05	5,53898E-05
Bacteria	*Gemmatimonadetes*					0	0	6,20688E-06	2,12386E-06	8,22341E-07
Bacteria	*Lentisphaerae*					4,78326E-05	0,0004671 8	0,000101259	0,000102985	0,000146835
Bacteria	*Nitrospirae*					0	0	2,1459E-06	0	0
Bacteria	*Parcubacteria*					0	0	6,24915E-07	4,02996E-07	5,61184E-06
Bacteria	*Planctomycetes*					4,62453E-06	0,007323585	0,010768298	0,009285983	0,001085587
Bacteria	*Proteobacteria*					0,024020771	0,031361222	0,048607827	0,031837887	0,069344776
Bacteria	*SR1*					0	0	1,41073E-05	0,0004425	0
Bacteria	*Spirochaetes*					8,21604E-06	0,024746571	0,030418188	0,026490124	0,041474055
Bacteria	*Synergistetes*					6,8467E-07	0,000992028	4,27386E-05	0,000601401	0,001769282
Bacteria	*Tenericutes*					0,003734463	0,001290828	0,00118606	0,002679968	0,022091917
Bacteria	*Verrucomicrobia*					0,001741859	0,009094064	0,015353211	0,020466149	0,001939325
Bacteria	*candidate division WPS-1*					1,08774E-06	0	3,5999E-06	0	0
Bacteria	*candidate division WPS-2*					0	0	3,52987E-07	0	0

Supplementary table S6. KEGG pathways predicted using PiCRUST to be significantly changed between different groups analyzed. Statistical tests were done in STAMP.

L1	L2	L3	*P values*	*P values* (corrected)	Warthogs				Pigs					
					AFR		SPA		COM		AFR		SPF	
					Mean rel. freq (%)	Std. dev (%)	Mean rel. freq (%)	Std. dev (%)	Mean rel. freq (%)	Std. dev (%)	Mean rel. freq (%)	Std. dev (%)	Mean rel. freq (%)	Std. dev (%)
CELLULAR PROCESSEs	Transport and Catabolism	Endocytosis	4.09781082157e-07	0.000123344105729	0.000342789461967	0.000523651413533	6.04685955554e-06	2.18777049229e-06	6.51869572722e-05	0.000147709537854	4.66858713314e-06	9.98578150365e-06	1.91291688442e-07	6.04917432935e-07
	Cell Growth and Death	Cell cycle	9.61485971729e-07	0.00028940727749	2.22273986045e-05	3.22010283245e-05	2.23500258085e-05	1.37561417117e-05	0.00018536723879	0.000225305045482	0.000517703042453	0.000544590600554	0.0	0.0
	Transport and Catabolism	Phagosome	9.61485971729e-07	0.00028940727749	2.22273986045e-05	3.22010283245e-05	2.23500258085e-05	1.37561417117e-05	0.00018536723879	0.000225305045482	0.000517728975253	0.000544598539299	0.0	0.0
	Cell Growth and Death	Meiosis - yeast	5.16394942911e-05	0.0155434877816	0.0065393545565	0.00212902614428	0.00934182286674	0.00272145561536	0.0048612292952	0.00157432936671	0.00694170701491	0.00377924513005	0.00185424875086	0.0019699332037
	Cell Motility	Cytoskeleton proteins	5.19452485522e-05	0.0156355198142	0.386854868843	0.0204445587024	0.3942697703	0.0101090862044	0.379892140406	0.0183170541873	0.365663928783	0.026101486572	0.415661994219	0.0116650795632
	Cell Growth and Death	Apoptosis	0.000138179726666	0.0415920977265	0.000666903462801	0.000974200541604	0.00155965780573	0.000786179263304	0.000763197903572	0.000497984541407	0.000722737959361	0.00210674299203	0.00160092740152	0.000968976639336
ENVIRONMEN	Signal Transduction	mTOR signaling pathway	9.61485971729e-07	0.00028940727749	2.22273986045e-05	3.22010283245e-05	2.23500258085e-05	1.37561417117e-05	0.00018536723879	0.000225305045482	0.000517703042453	0.000544590600554	0.0	0.0
	Signal Transduction	Calcium signaling pathway	1.5373760758e-06	0.000462750198815	6.6479833139e-06	3.85146764023e-06	3.60123132175e-06	2.3001698728e-06	8.12669355932e-07	1.24610254997e-06	0.000123055167355	0.000215798793605	3.17083042996e-07	1.00270462329e-06
	Translation	mRNA surveillance pathway	1.5063661551e-06	0.000453416212685	4.49101491967e-05	6.44436380551e-05	4.48706180361e-05	2.78067220451e-05	0.00037073447758	0.000450610090964	0.00103887376991	0.00108968600916	5.73875065325e-07	1.8147522988e-06

**TAL INFO. PROCES.**	Transcription	Basal transcription factors	8.65339616404e-06	0.00260467224538	0.00216437483679	0.00108239629429	0.0027585577531	0.000910552459223	0.00184690604077	0.000685147240267	0.00427936375103	0.00295762300631	0.000272619302742	0.000135553271426
	Translation	Aminoacyl-tRNA biosynthesis	5.6517193613e-05	0.0170116752775	1.22767871563	0.0305247168651	1.29947752261	0.0237456575655	1.27625821222	0.0291433353907	1.2188459441	0.0487726058102	1.27940861399	0.0240738426979
	Replication and Repair	Mismatch repair	9.08257872495e-05	0.0273385619621	0.848769211142	0.0276914553038	0.891645658913	0.0245284061325	0.883446989728	0.031503635406	0.847570557358	0.0330492250846	0.896031377282	0.0114063311112
	Replication and Repair	DNA replication	0.000107524104324	0.0323647554015	0.668660814322	0.0248691897252	0.710302756677	0.0242832269427	0.707569520396	0.0342659834255	0.671966973623	0.0288503936423	0.710352244698	0.0101620072232
**HUMAN DISEASES**	Infectious Diseases	Vibrio cholerae infection	8.79348623188e-07	0.000264683935579	2.35290129049e-05	3.23966942504e-05	2.6324279407e-05	1.56838062678e-05	0.000186567075269	0.000225434585571	0.000522716705692	0.000541544374191	0.0	0.0
	Infectious Diseases	Measles	9.61485971729e-07	0.00028940727749	2.22273986045e-05	3.22010283245e-05	2.23500258085e-05	1.37561417117e-05	0.00018536723879	0.000225305045482	0.000517703042453	0.000544590600554	0.0	0.0
	Infectious Diseases	Hepatitis C	9.61485971729e-07	0.00028940727749	2.22273986045e-05	3.22010283245e-05	2.23500258085e-05	1.37561417117e-05	0.00018536723879	0.000225305045482	0.000517703042453	0.000544590600554	0.0	0.0
	Infectious Diseases	Bacterial invasion of epithelial cells	9.83252094004e-07	0.000295958880295	0.00112251641394	0.00081190792401	0.000213808110815	8.75434155005e-05	0.000153269525336	8.61209892503e-05	0.00121520392184	0.00124478120419	2.87190229117e-05	4.3282297284e-05
	Infectious Diseases	Chagas disease (American trypanosomiasis)	1.64024166587e-06	0.000493712741428	0.00186731347514	0.00164199777596	0.000170360218407	0.000100718922444	2.77424775429e-05	2.41984421175e-05	0.00121265556663	0.00136051011194	0.000179262778424	0.00014063524857
	Infectious Diseases	Pathogenic Escherichia coli infection	2.22247661777e-06	0.00066896546195	5.22007092926e-07	1.80828561372e-06	8.32709259379e-07	1.66541851876e-06	0.0	0.0	1.70806652366e-05	2.06417749538e-05	0.0	0.0

	Infectious Diseases	Shigellosis	3.6348101223e-06	0.00109407784681	4.0764152518e-06	1.33569890352e-05	2.33158592626e-06	4.66317185252e-06	0.0	0.0	2.30442725544e-05	4.30083682978e-05	0.0	0.0
	Immune System Diseases	Primary immunodeficiency	4.71143579229e-05	0.0141814217348	0.0445713744246	0.00244369229449	0.0371204369425	0.00413895277996	0.0403655228009	0.00249074415874	0.0395873779546	0.00379578916119	0.0346503710396	0.00496613781403
	Cancers	Bladder cancer	6.02103444733e-05	0.0181233136865	0.000455363353351	0.00110015392413	0.000230304208905	9.27939501339e-05	5.4709843648e-05	2.15514585198e-05	0.00128604831989	0.00145313753016	3.09085414723e-05	4.09959089383e-05
METABOLISM	Energy Metabolism	Photosynthesis - antenna proteins	9.91665112067e-07	0.000298491198732	0.000111663628709	5.64537251771e-05	6.22296602526e-05	3.96001621162e-05	1.67276425361e-05	2.24067789615e-05	0.00208984115659	0.00366771301718	5.23805905862e-06	1.49651903465e-05
	Carbohydrate Metabolism	Glyoxylate and dicarboxylate metabolism	2.20587516423e-06	0.000663968424434	0.504270007114	0.012676943859	0.479442140927	0.010142197321	0.500599097573	0.0141642612882	0.514570114208	0.024500026767	0.44842750135	0.015801360711
	Glycan Biosynthesis and Metabolism	Glycosphingolipid biosynthesis - lacto and neolacto series	2.62318573932e-06	0.000789578907536	1.33115627077e-06	3.61263663415e-06	1.58202514758e-05	5.5510996201e-06	4.07178972273e-06	3.80007934163e-06	1.83290627341e-05	2.44051310188e-05	6.15199857736e-05	5.90743266251e-05
METABOLISM	Biosynthesis of Other Secondary Metabolites	Isoflavonoid biosynthesis	2.84833348092e-06	0.000857348377757	0.000662379197305	0.000571167433547	8.17632426154e-05	5.01046288092e-05	0.000422441225396	0.000294587887292	0.000906386892966	0.00115414719023	5.73875065325e-07	1.8147522988e-06
	Lipid Metabolism	Steroid biosynthesis	1.11887024793e-05	0.00336779944627	0.00389132282006	0.00266310216384	0.000655401730609	8.4313534851e-05	0.00440987501425	0.00552393428755	0.00671185419019	0.00430665047823	0.000450090765211	0.000419967606825
	Biosynthesis of Other Secondary Metabolites	Indole alkaloid biosynthesis	1.12729592823e-05	0.00339316074398	2.65758941192e-05	2.18148385948e-05	0.000102286034689	7.51075025417e-05	1.19236516435e-05	1.0545746143e-05	4.36774455771e-06	2.70678939124e-06	1.39037898096e-05	1.20483290448e-05

	Biosynthesis of Other Secondary Metabolites	Betalain biosynthesis	1.3107899566e-05	0.00394547776937	2.77682710345e-05	2.15021453025e-05	0.0001081629383753	7.36141428172e-05	1.21804090754e-05	1.03918459395e-05	4.7851132443e-06	3.17264567354e-06	1.41920099257e-05	1.20148447353e-05
	Metabolism of Cofactors and Vitamins	Thiamine metabolism	1.77679119572e-05	0.00534814149912	0.520059981429	0.0163509370157	0.502639997791	0.00779542099725	0.497573596091	0.0190072808866	0.492587738997	0.0195382498992	0.535738470061	0.0125150897152
	Glycan Biosynthesis and Metabolism	Various types of N-glycan biosynthesis	2.2125229313e-05	0.0066596940232	0.000847397851202	0.00101659406246	0.000138427116447	6.6988840598e-05	0.000604296738004	0.000347865632443	0.000708101730544	0.000562215577825	4.49180164645e-05	6.19569354132e-05
	Carbohydrate Metabolism	Ascorbate and aldarate metabolism	3.21036509008e-05	0.00966319892113	0.0938887942902	0.0149357203976	0.0875686048813	0.00720927471785	0.0967317688375	0.00960564748208	0.107099999872	0.00816983001043	0.0789469670585	0.00945416102925
	Enzyme Families	Cytochrome P450	3.2334590559e-05	0.00973271175826	8.2317664058e-07	1.44280537811e-06	5.93968830523e-06	3.95614384366e-06	0.0	0.0	4.42330464191e-06	6.74766840181e-06	8.89066033201e-07	2.22886935409e-06
	Amino Acid Metabolism	Tryptophan metabolism	6.40129399534e-05	0.019267894926	0.158736409183	0.0445127395087	0.13748536757	0.00672017966174	0.138638300515	0.0222328508945	0.161902344292	0.0279926121283	0.116954098858	0.01215207806
	Xenobiotics Biodegradation and Metabolism	Caprolactam degradation	0.000111163581496	0.0334602380304	0.0267789543816	0.0189501408953	0.0166959249387	0.00318572994875	0.0178938966622	0.00715410173103	0.0283914104892	0.0112523050861	0.0119489092874	0.00487277962568
	Energy Metabolism	Carbon fixation pathways in prokaryotes	0.000146803103163	0.044187734052	0.977241042032	0.0542353185094	1.03670178548	0.0383416680114	1.06532643057	0.0457424852137	1.01475518498	0.0298934180376	1.06909139864	0.0268037146514

**ORGANISMAL SYSTEMS**	Immune System	Fc gamma R-mediated phagocytosis	4.09781082157e-07	0.000123344105729	0.000342789461967	0.000523651413533	6.04685955554e-06	2.18777049229e-06	6.51869572722e-05	0.000147709537854	4.66858713314e-06	9.98578150365e-06	1.91291688442e-07	6.04917432935e-07
	Endocrine System	GnRH signaling pathway	4.09781082157e-07	0.000123344105729	0.000342789461967	0.000523651413533	6.04685955554e-06	2.18777049229e-06	6.51869572722e-05	0.000147709537854	4.66858713314e-06	9.98578150365e-06	1.91291688442e-07	6.04917432935e-07
	Digestive System	Bile secretion	4.64191398876e-07	0.000139721611062	0.000920887914386	0.000806030453882	0.00231139167065	0.0009707450345	0.000441791850539	0.000191964695549	0.00152896770611	0.001147629675	1.98712483789e-06	3.48784587731e-06
	Nervous System	Glutamatergic synapse	1.45564846773e-06	0.000438150188786	0.0984749862613	0.00275767243797	0.104479002346	0.00470898883099	0.101341572865	0.00300343070117	0.0970547357937	0.0030590763592	0.108339812062	0.0020428690882
	Endocrine System	Adipocytokine signaling pathway	3.70308047192e-05	0.0111462722205	0.0698923227173	0.00536551295609	0.0796454379488	0.00257528661938	0.0778338209433	0.0101125369081	0.0676475279967	0.00805251605304	0.0844790629791	0.00461817045052
	Endocrine System	PPAR signaling pathway	0.000122022753208	0.0367288487156	0.108924955447	0.00995287393643	0.119899280353	0.00236974997165	0.113038325144	0.0070209516711	0.107197164684	0.0068511341699	0.122791903937	0.0058796060649
